# Supplementary material for: HPLC-MS/MS Oxylipin Analysis of Plasma from Amyotrophic Lateral Sclerosis Patients
Source: Biomedicines. 2022 Mar 15;10(3):674. doi: 10.3390/biomedicines10030674 (PMC8945419; doi:10.3390/biomedicines10030674)
Supplement: Supplementary file 1 [file biomedicines-10-00674-s001.zip › Figure S1 - Fragmentation spectra.pdf]

Figure S1 – Fragmentation spectra of oxylipin's standard solutions

4-HDoHE

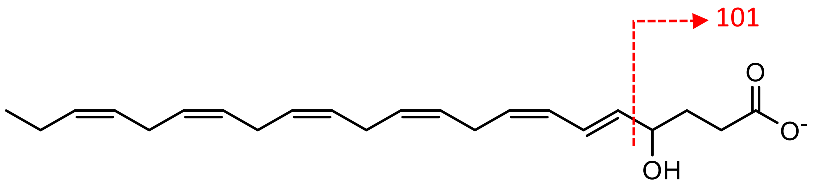
101
O
O-
OH

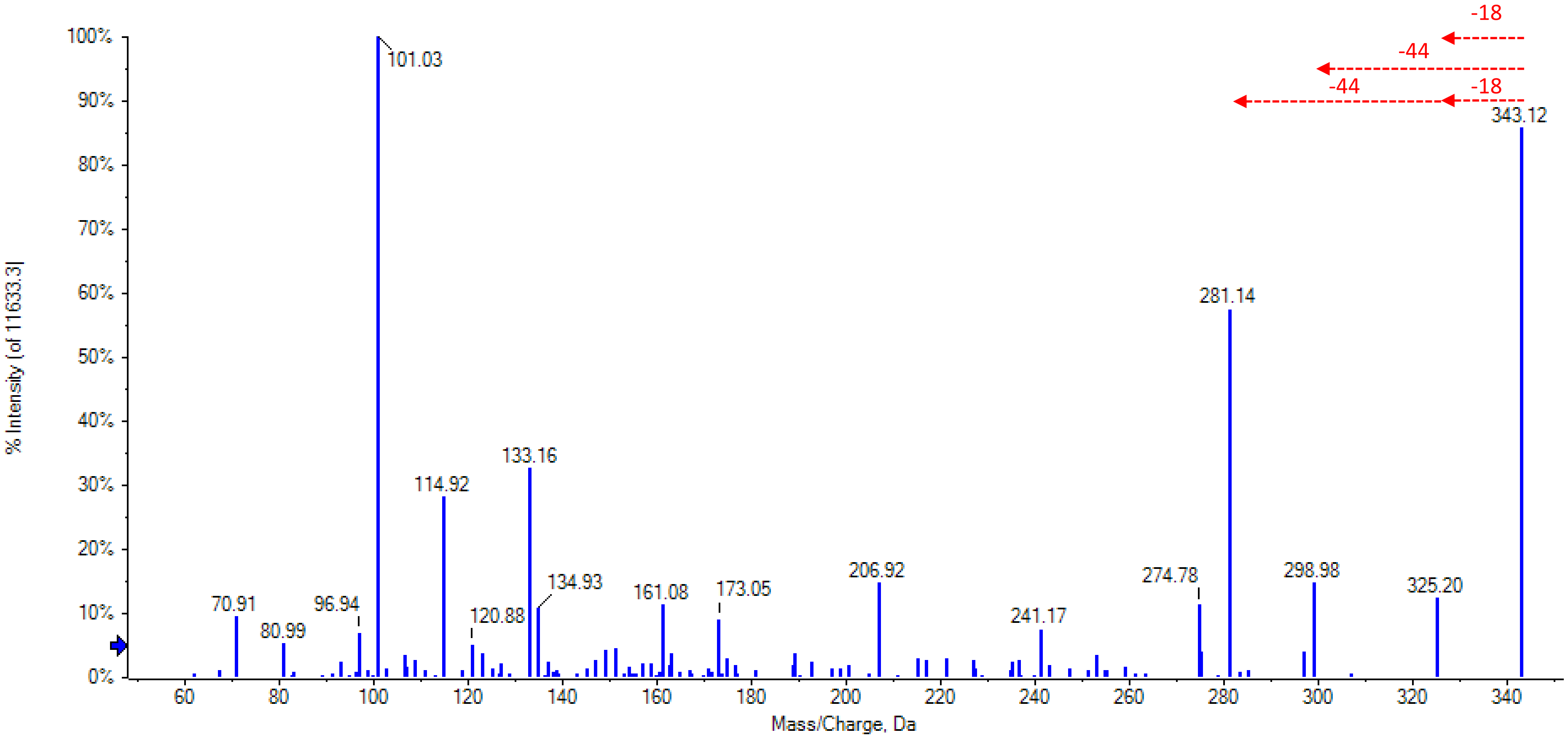
-18
-44
-44
-18
100%
90%
80%
70%
60%
50%
40%
30%
20%
10%
0%
% Intensity (of 11633.3)
101.03
343.12
281.14
133.16
114.92
206.92
298.98
325.20
274.78
173.05
161.08
134.93
120.88
241.17
70.91
96.94
80.99
60
80
100
120
140
160
180
200
220
240
260
280
300
320
340
Mass/Charge, Da

7-HDoHE

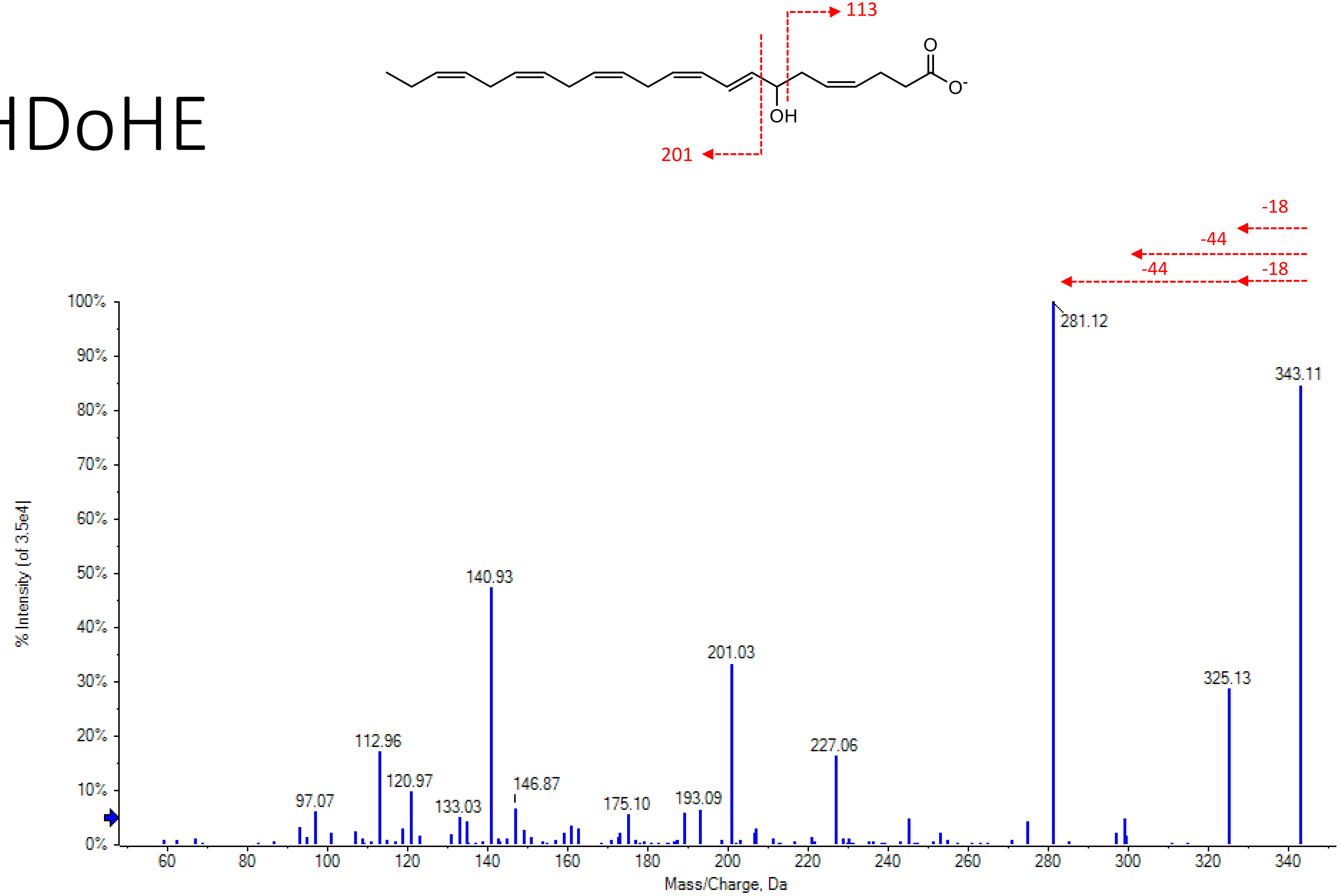

113
O
O-
OH
201
-18
-44
-44
-18
100%
90%
80%
70%
60%
50%
40%
30%
20%
10%
0%
% Intensity (of 3.5e4)
281.12
343.11
140.93
201.03
325.13
227.06
112.96
120.97
146.87
97.07
133.03
175.10
193.09
60
80
100
120
140
160
180
200
220
240
260
280
300
320
340
Mass/Charge, Da

13-HDoHE

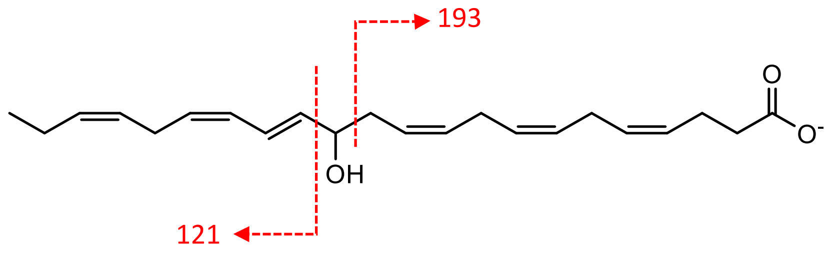

193
OH
121

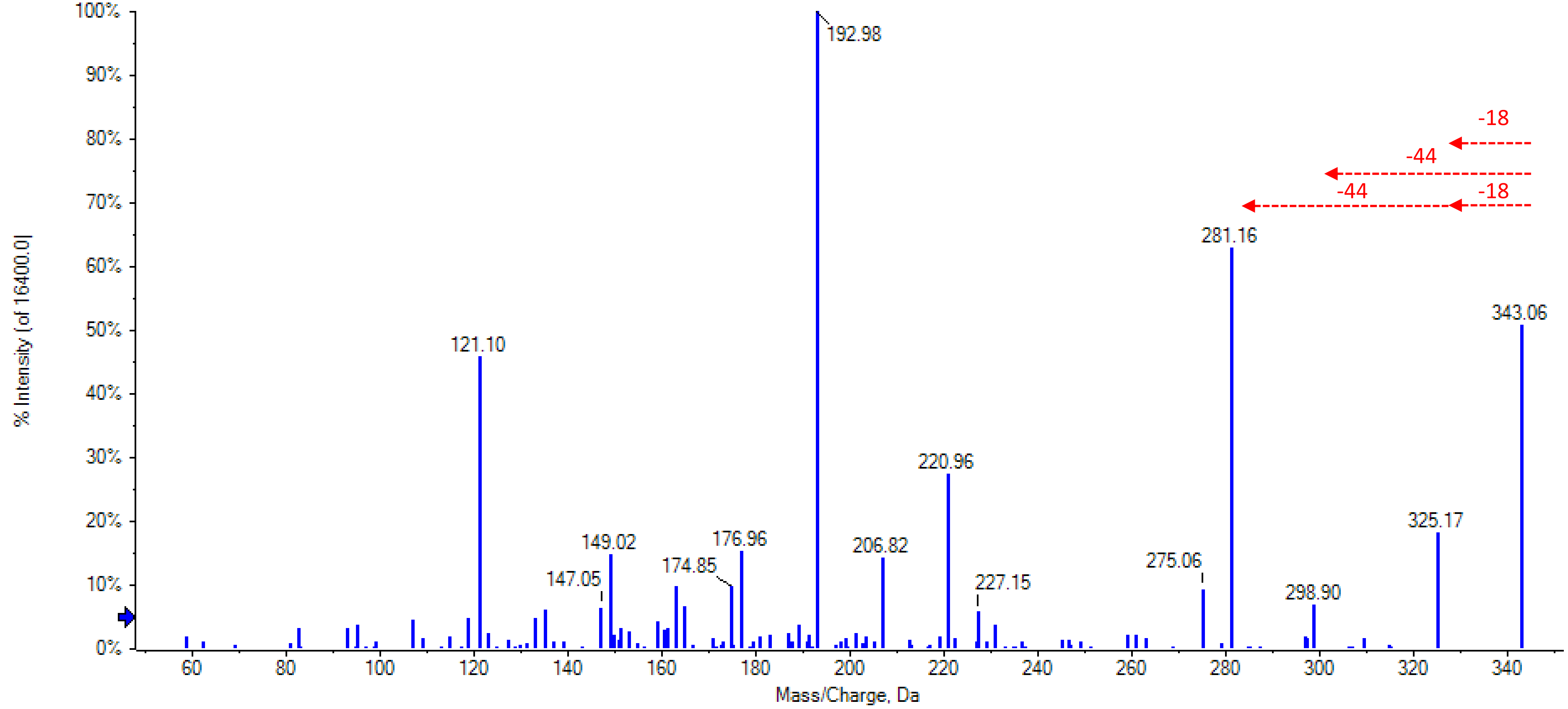

-18
-44
-44
-18
192.98
281.16
343.06
121.10
220.96
325.17
149.02
176.96
174.85
147.05
206.82
227.15
275.06
298.90
% Intensity [of 16400.0]
Mass/Charge, Da

16-HDoHE

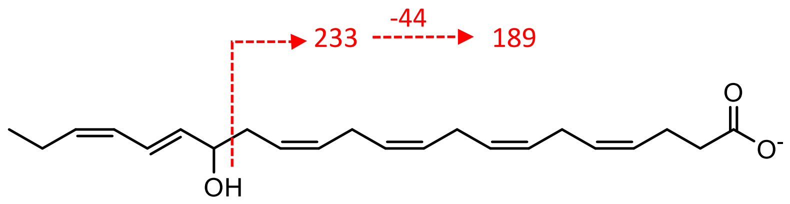
233
-44
189
OH
O
O-

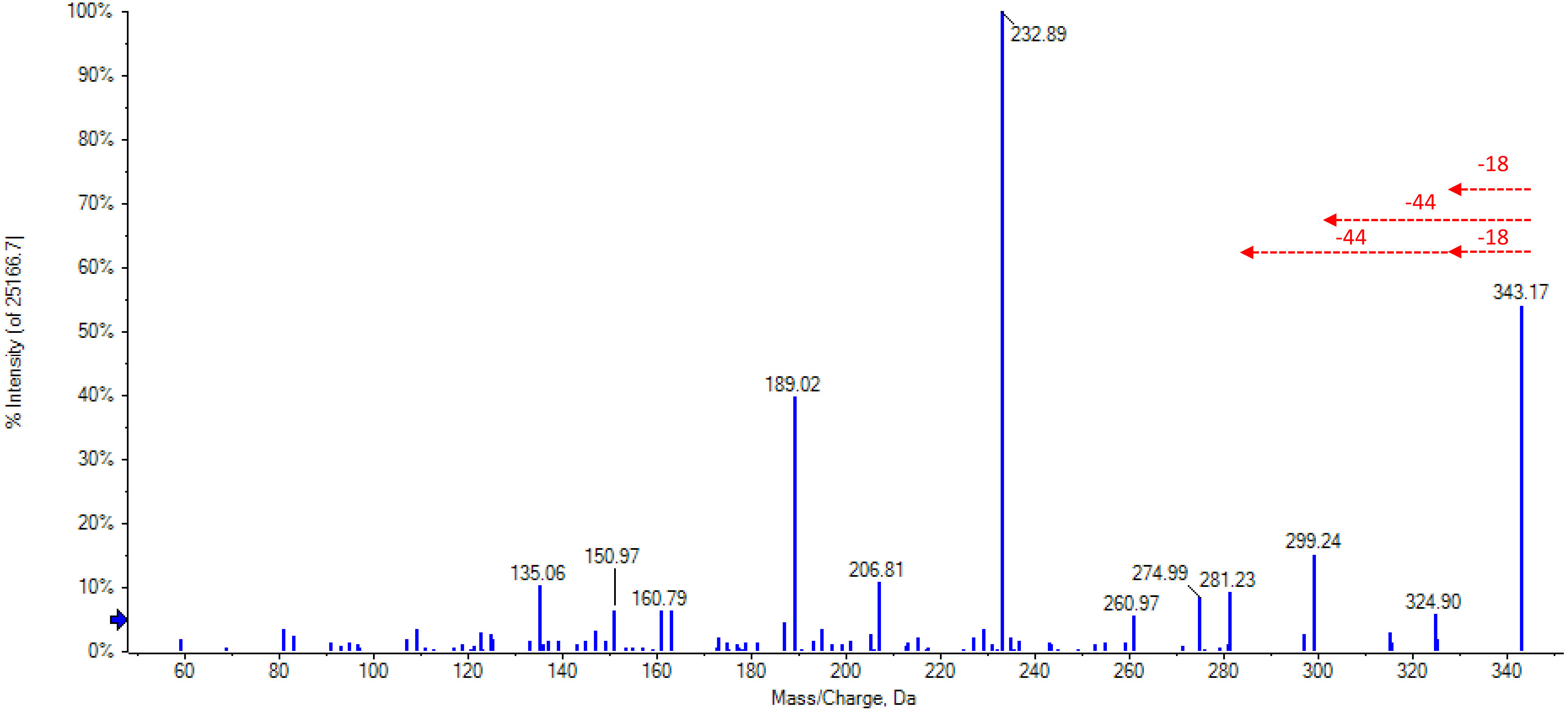
232.89
343.17
189.02
299.24
206.81
135.06
150.97
160.79
274.99
281.23
260.97
324.90
-18
-44
-44
-18
% Intensity [of 25166.7]
Mass/Charge, Da

14-HDoHE

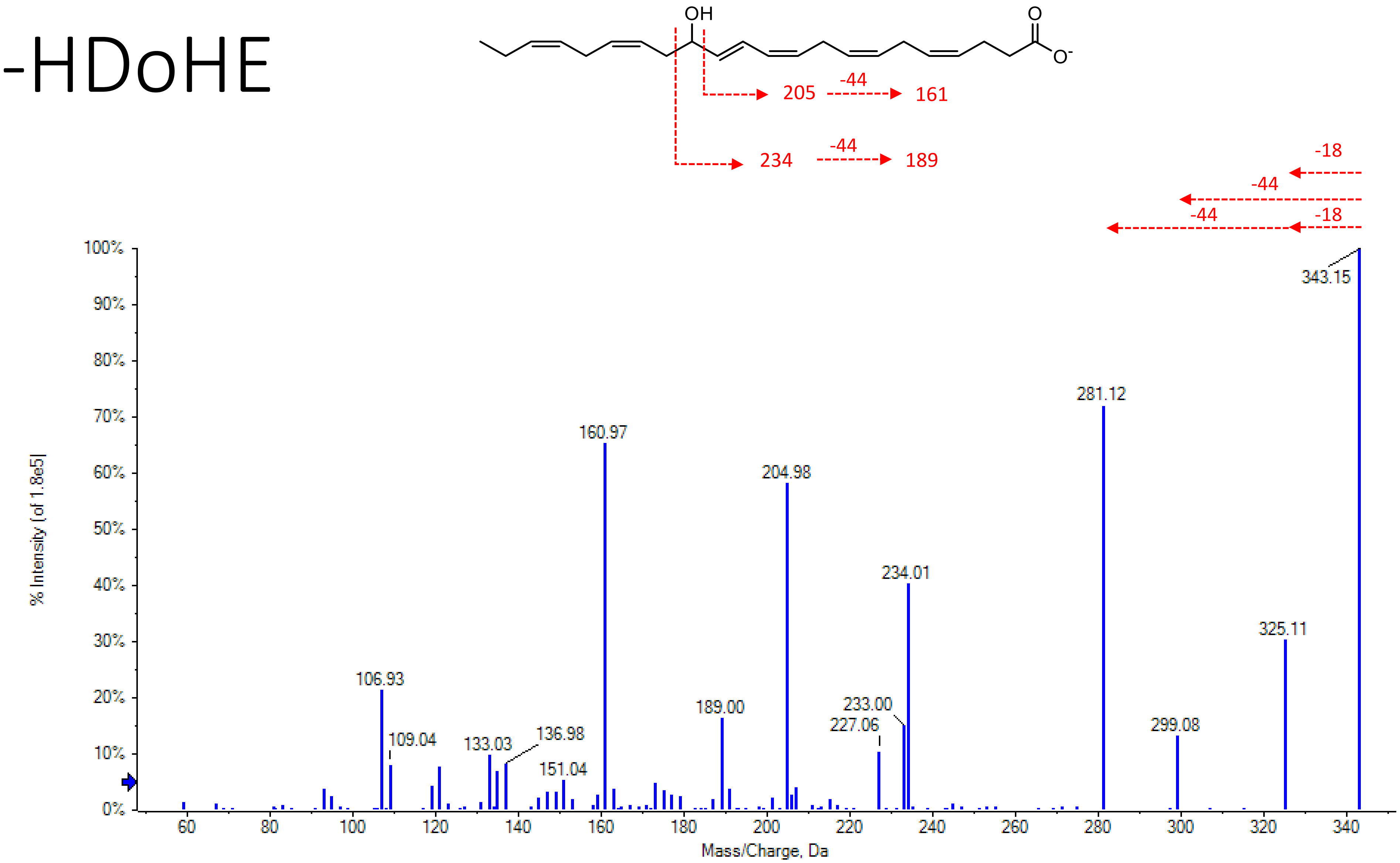

OH
O
O-
205
-44
161
234
-44
189
-18
-44
-44
-18
343.15
281.12
160.97
204.98
234.01
325.11
106.93
109.04
133.03
136.98
151.04
189.00
233.00
227.06
299.08
100%
90%
80%
70%
60%
50%
40%
30%
20%
10%
0%
% Intensity (of 1.8e5)
60
80
100
120
140
160
180
200
220
240
260
280
300
320
340
Mass/Charge, Da

PD1

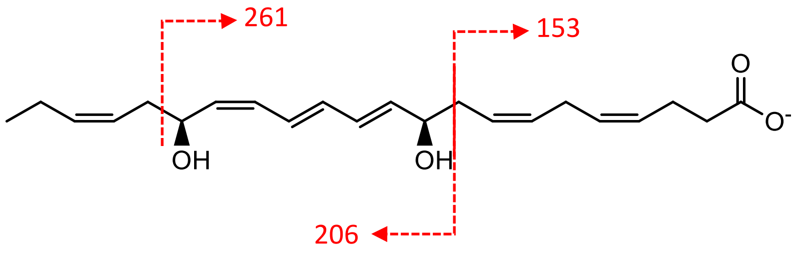
261
153
206
OH
OH
O
O-

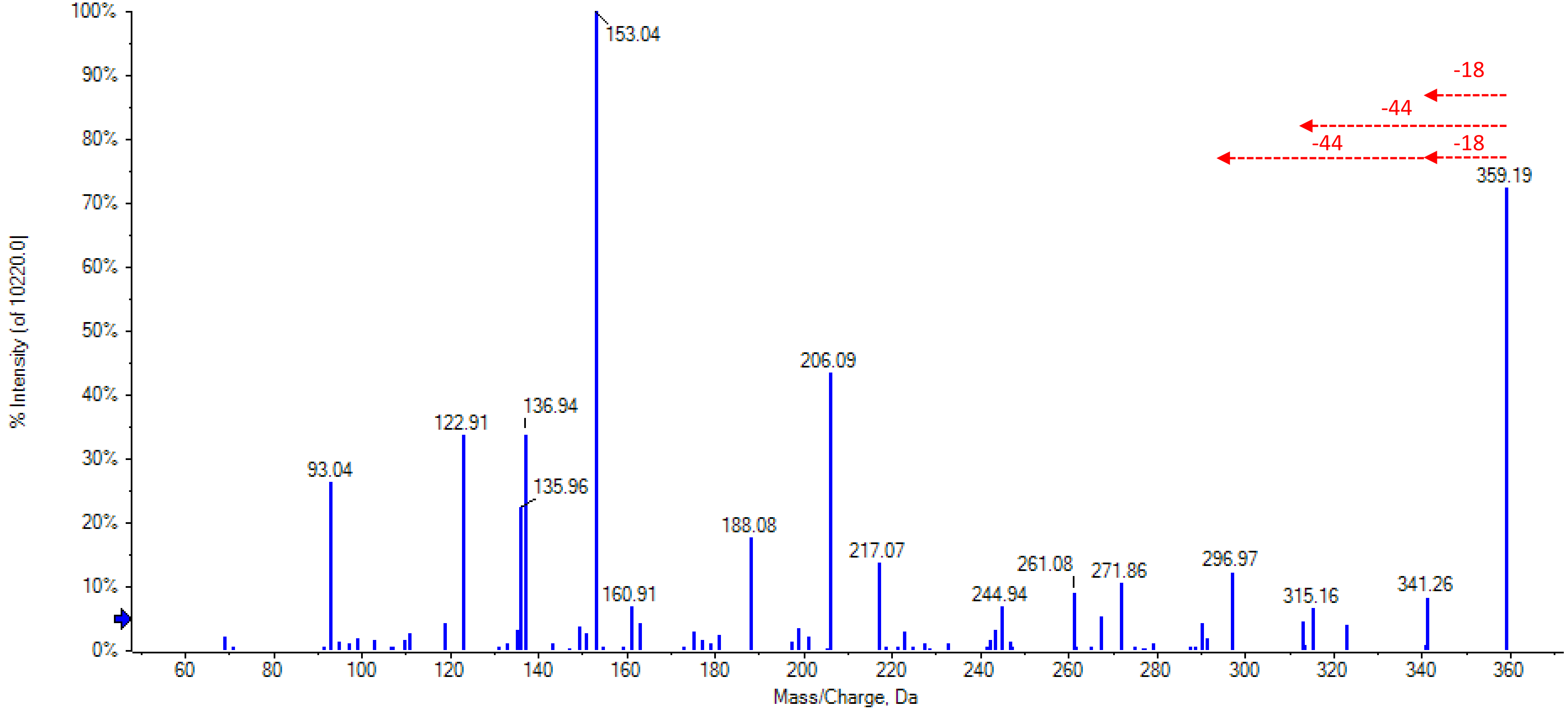
-18
-44
-44
-18
359.19
153.04
206.09
136.94
122.91
135.96
93.04
188.08
217.07
160.91
261.08
271.86
296.97
244.94
315.16
341.26
% Intensity [of 10220.0]
Mass/Charge, Da

MaR1

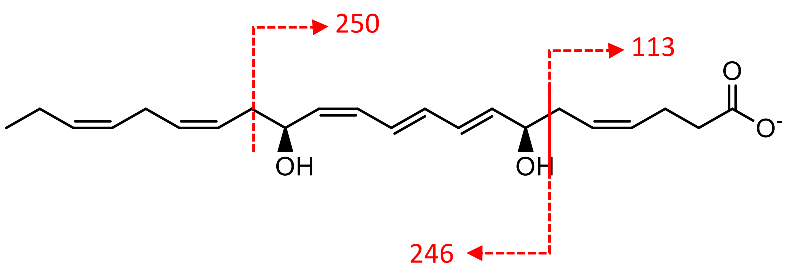

250
113
246
OH
OH
O
O-

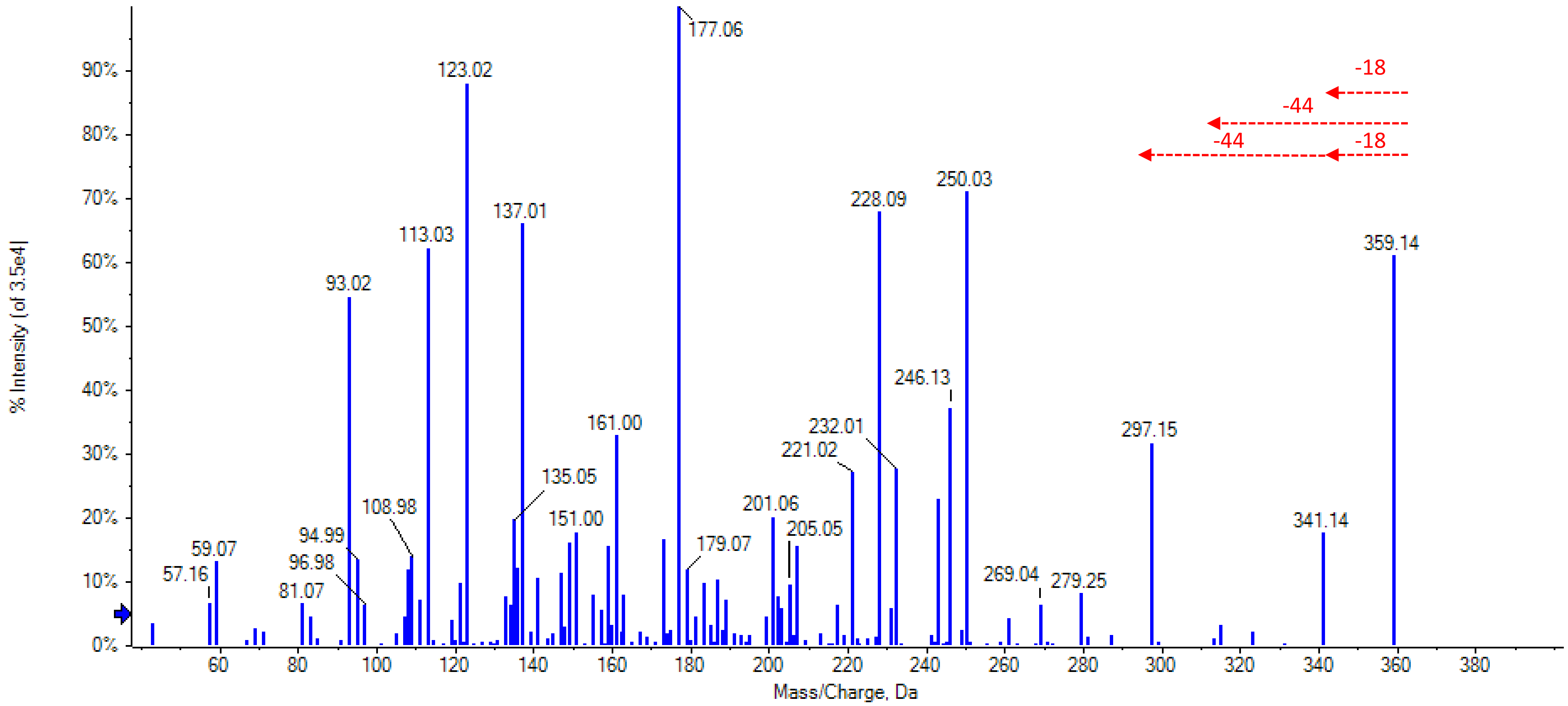

% Intensity (of 3.5e4)
Mass/Charge, Da
177.06
123.02
137.01
113.03
93.02
228.09
250.03
359.14
246.13
161.00
232.01
221.02
297.15
135.05
151.00
108.98
201.06
205.05
179.07
94.99
96.98
81.07
59.07
57.16
269.04
279.25
341.14
-18
-44
-44
-18

RvD mix

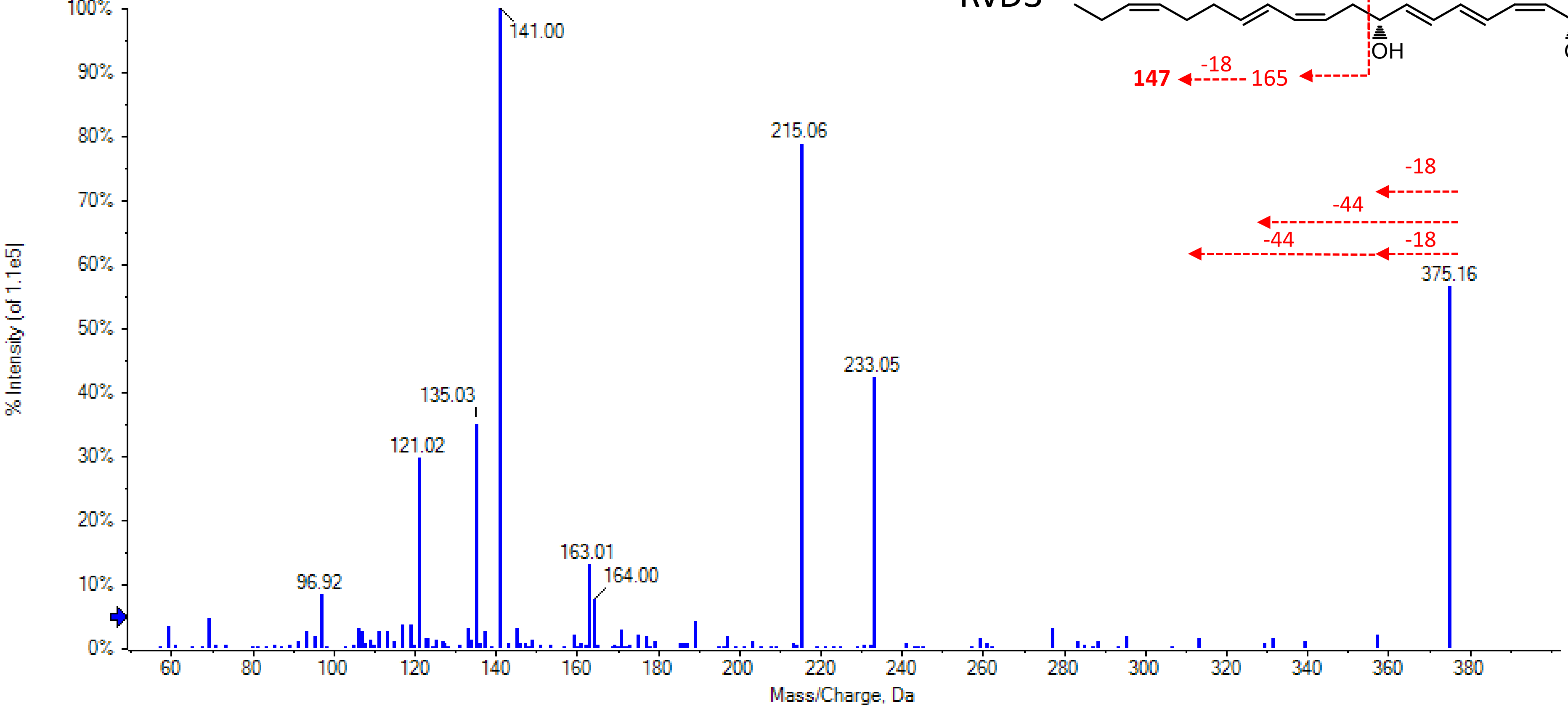

% Intensity [of 1.1e5]
Mass/Charge, Da
141.00
215.06
233.05
135.03
121.02
163.01
164.00
96.92
375.16
-18
-44
-44
-18

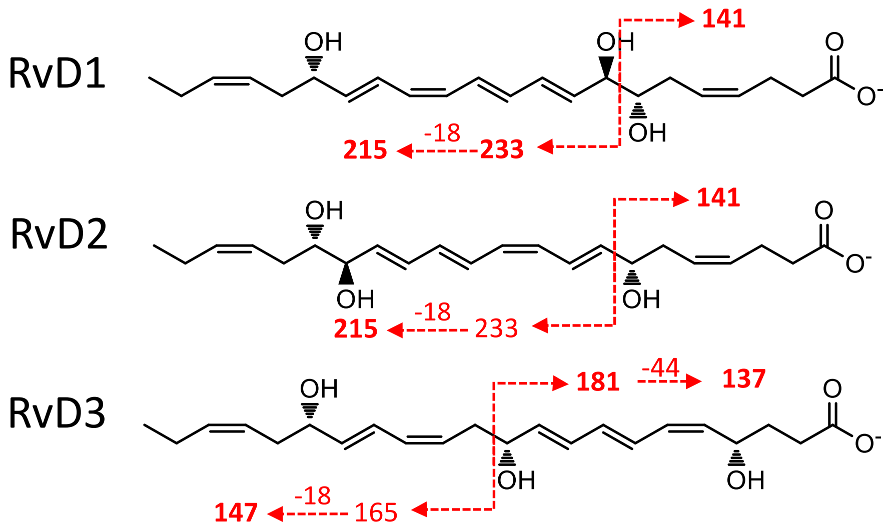

RvD1
OH
OH
OH
141
215
-18
233
RvD2
OH
OH
OH
141
215
-18
233
RvD3
OH
OH
OH
181
-44
137
147
-18
165

RvD5

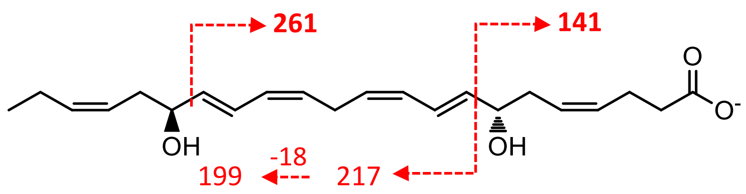
261
141
OH
OH
O
O-
199
-18
217

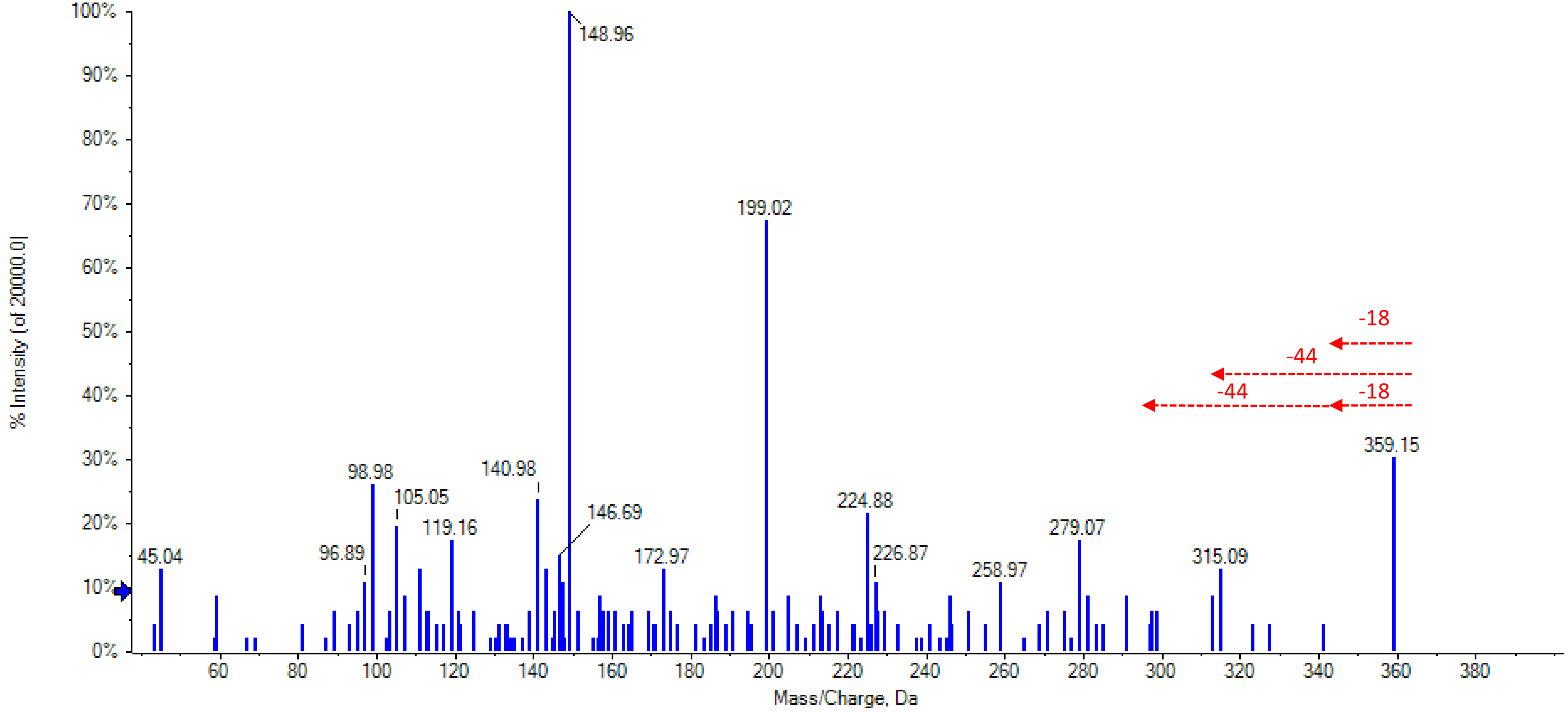
100%
90%
80%
70%
60%
50%
40%
30%
20%
10%
0%
% Intensity [of 20000.0]
148.96
199.02
359.15
98.98
140.98
105.05
146.69
224.88
119.16
279.07
45.04
96.89
172.97
226.87
258.97
315.09
-18
-44
-44
-18
60
80
100
120
140
160
180
200
220
240
260
280
300
320
340
360
380
Mass/Charge, Da

12-HEPE

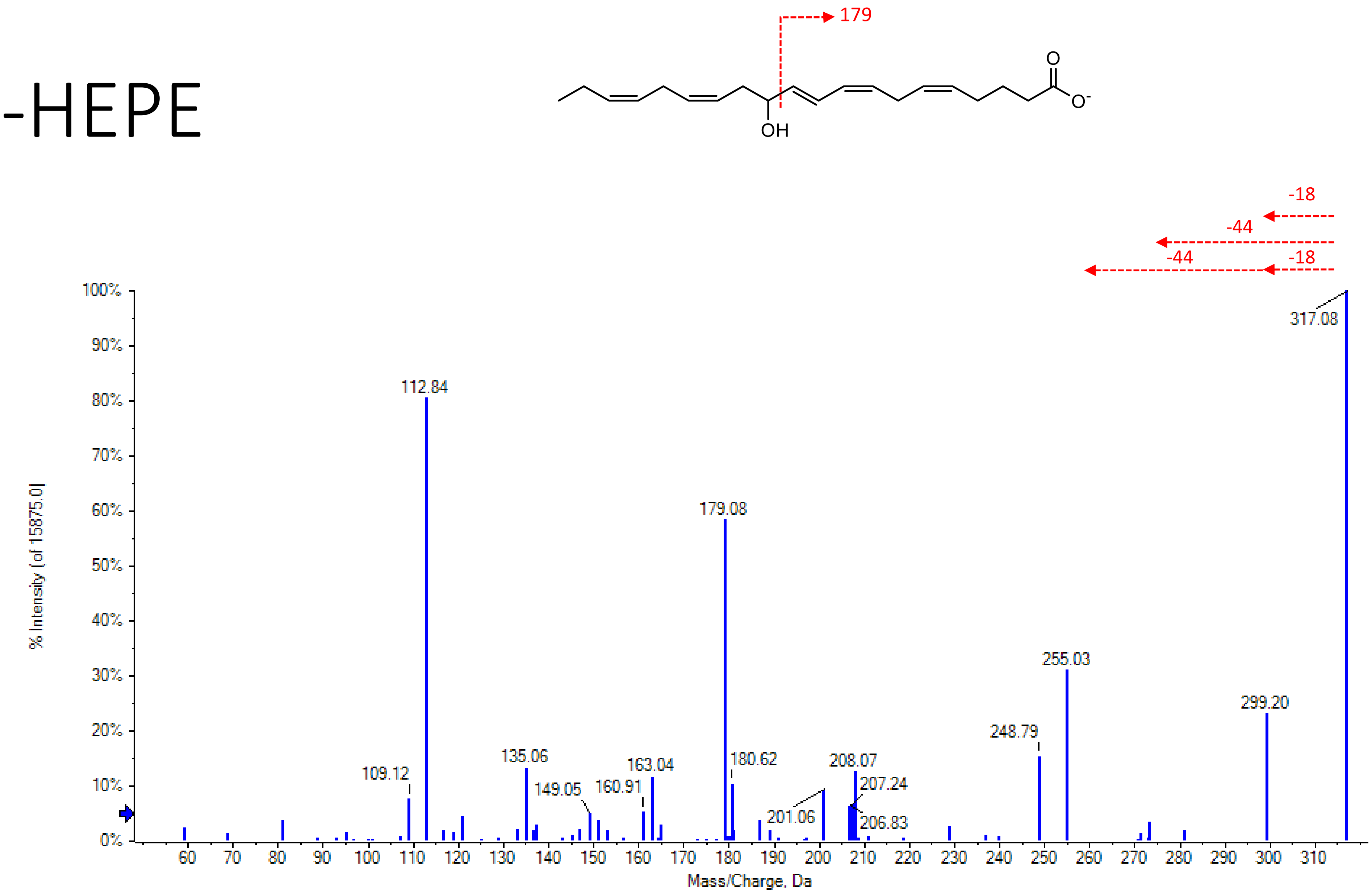
179
O
O-
OH
-18
-44
-44
-18
317.08
112.84
179.08
255.03
299.20
248.79
135.06
109.12
149.05
160.91
163.04
180.62
201.06
208.07
207.24
206.83
100%
90%
80%
70%
60%
50%
40%
30%
20%
10%
0%
% Intensity (of 15875.0)
60 70 80 90 100 110 120 130 140 150 160 170 180 190 200 210 220 230 240 250 260 270 280 290 300 310
Mass/Charge, Da

18-HEPE

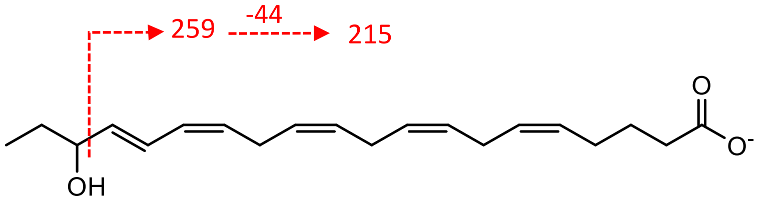

259
-44
215
O
O-
OH

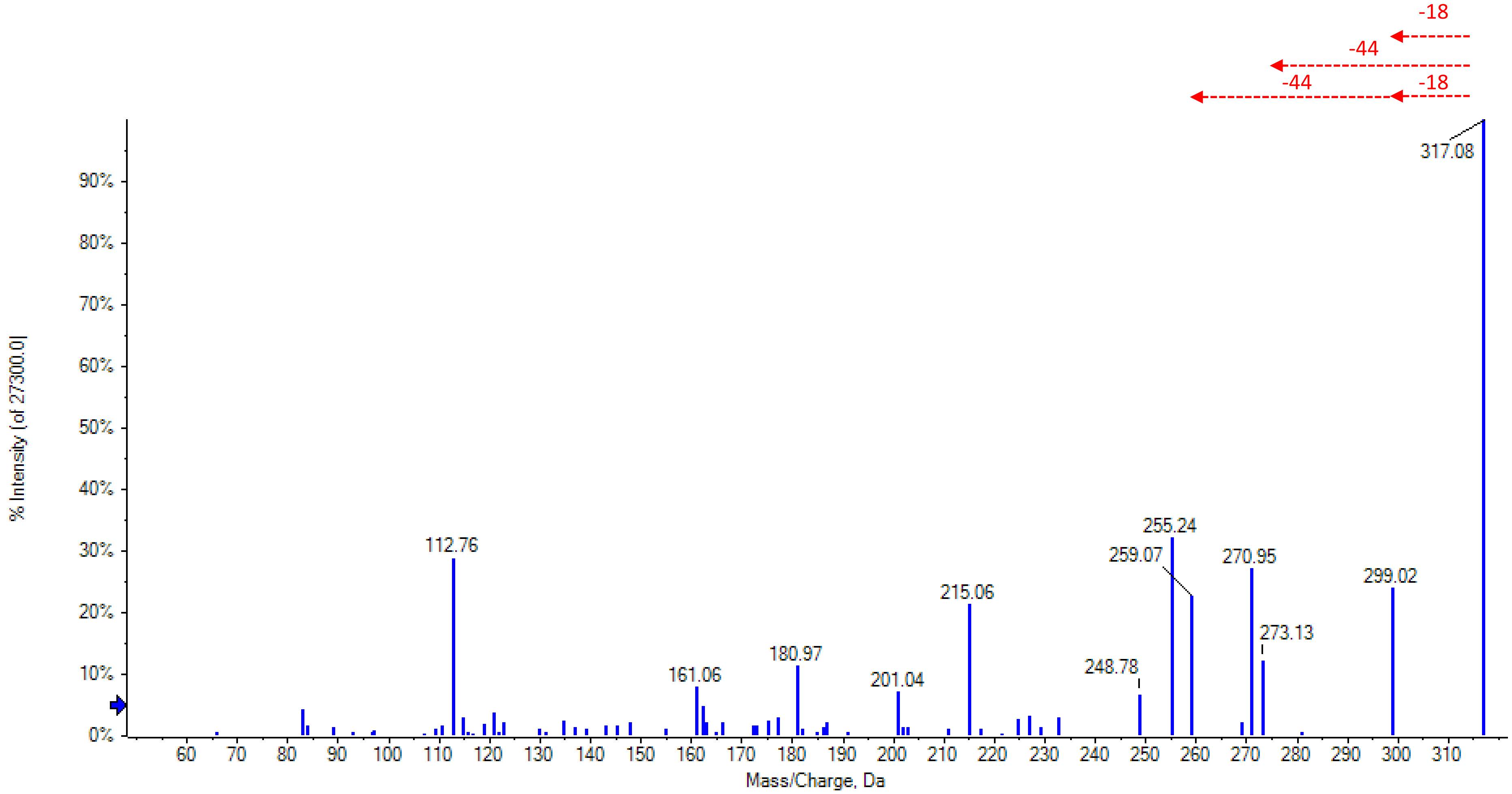

-18
-44
-44
-18
317.08
112.76
161.06
180.97
201.04
215.06
248.78
255.24
259.07
270.95
273.13
299.02
90%
80%
70%
60%
50%
40%
30%
20%
10%
0%
% Intensity (of 27300.0)
60 70 80 90 100 110 120 130 140 150 160 170 180 190 200 210 220 230 240 250 260 270 280 290 300 310
Mass/Charge, Da

RvE1

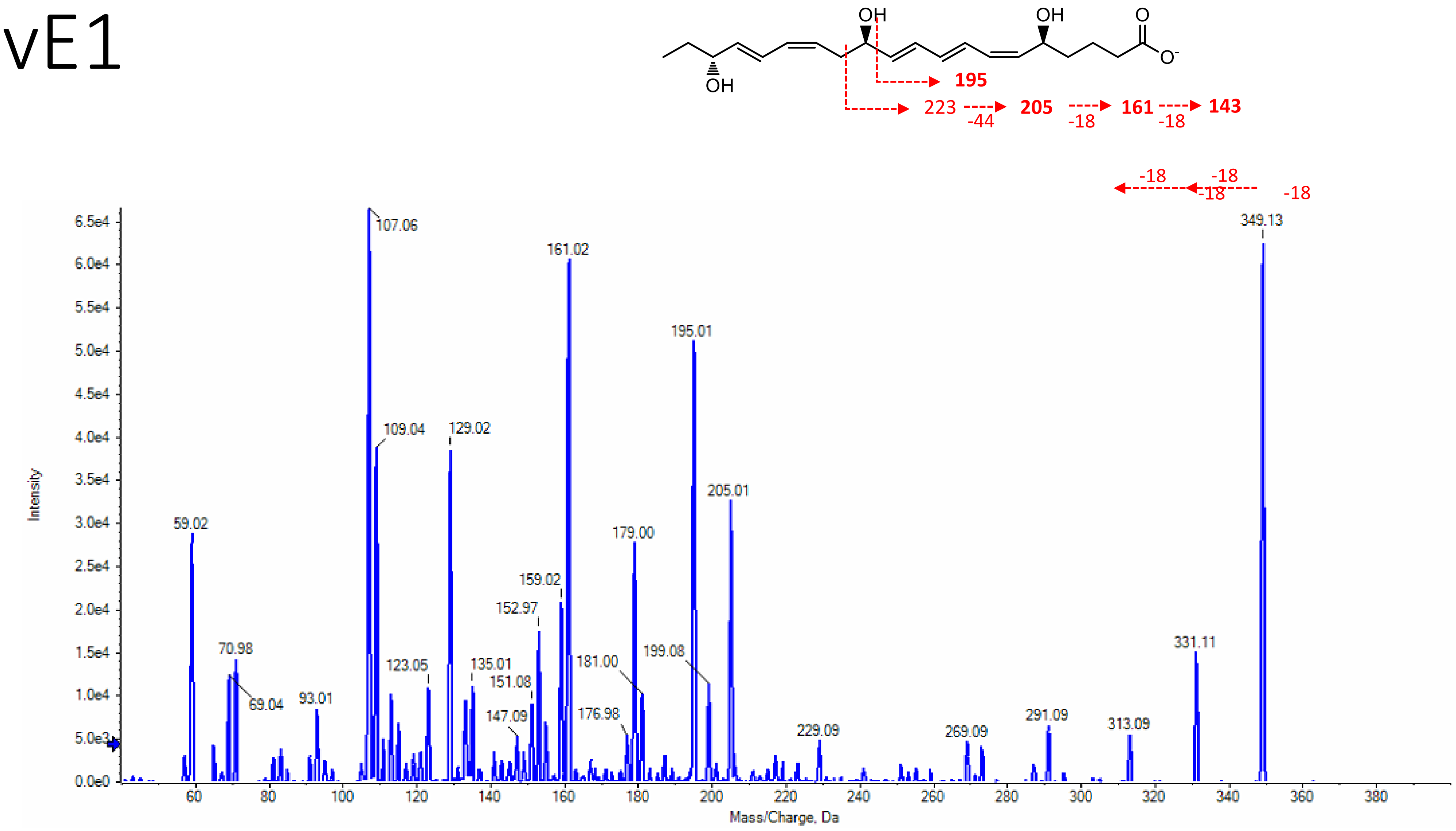
OH
OH
O
OH
O-
195
223
-44
205
-18
161
-18
143
-18
-18
-18
-18
349.13
331.11
313.09
291.09
269.09
229.09
205.01
199.08
195.01
181.00
179.00
176.98
161.02
159.02
152.97
151.08
147.09
135.01
129.02
123.05
109.04
107.06
93.01
70.98
69.04
59.02
Intensity
Mass/Charge, Da

LTB4

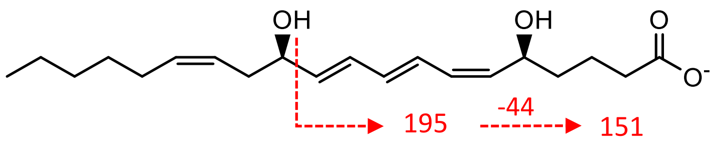
OH
OH
O
O-
195
-44
151

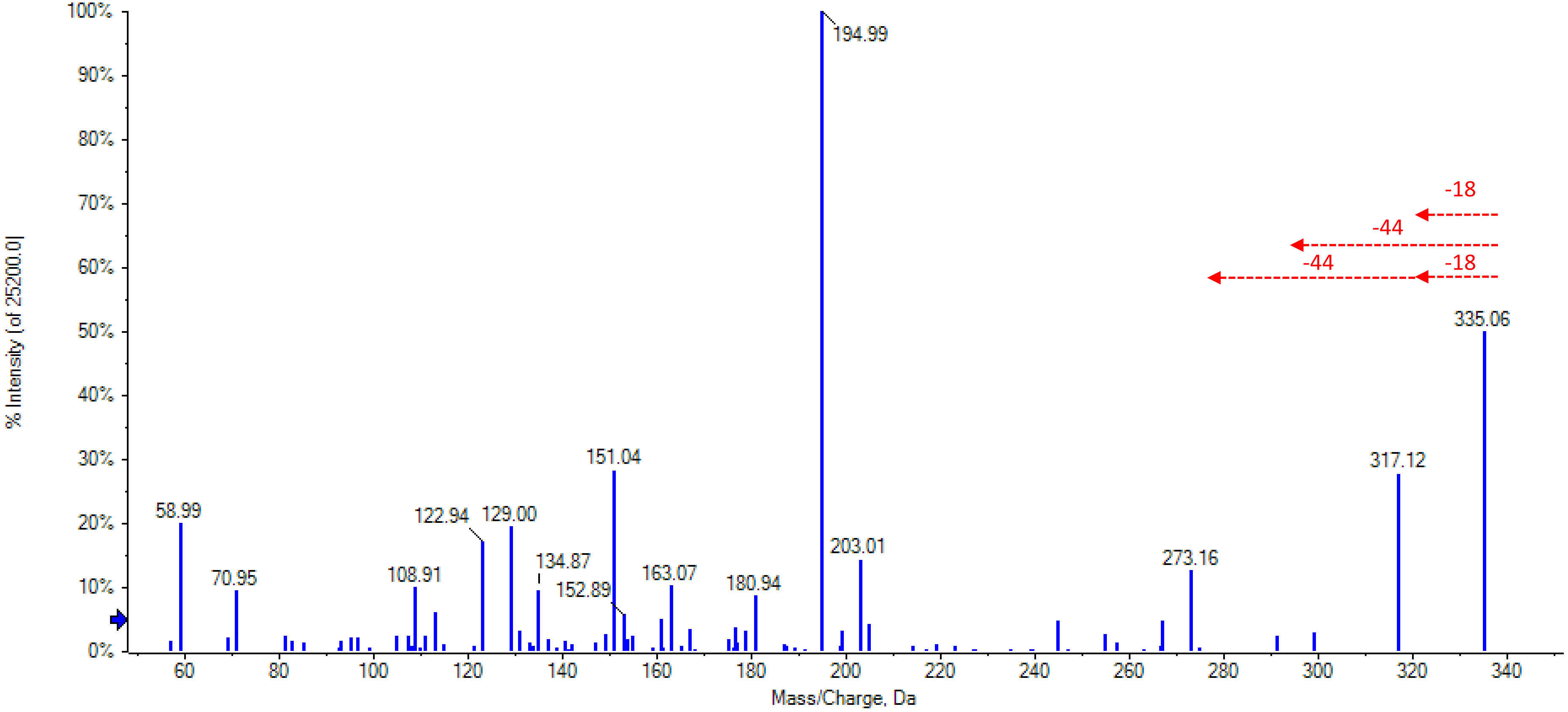
-18
-44
-44
-18
194.99
335.06
317.12
151.04
58.99
122.94
129.00
134.87
152.89
163.07
180.94
203.01
273.16
70.95
108.91
% Intensity [of 25200.0]
Mass/Charge, Da

EpOME mix

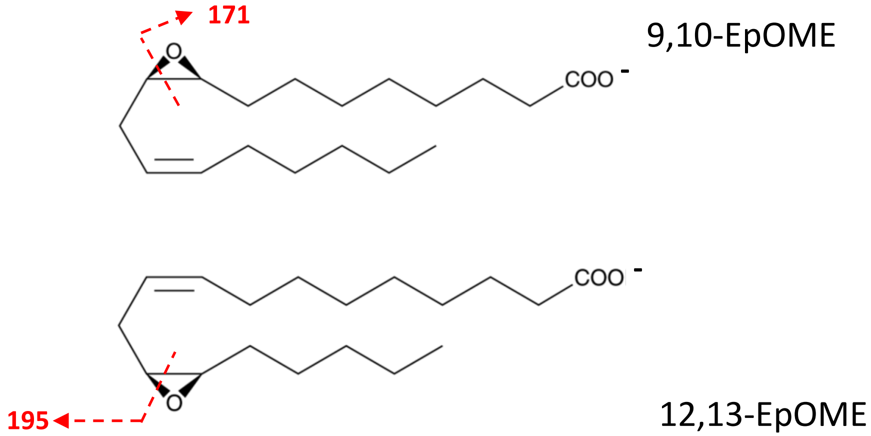

171
COO -
9,10-EpOME
COO -
195
12,13-EpOME

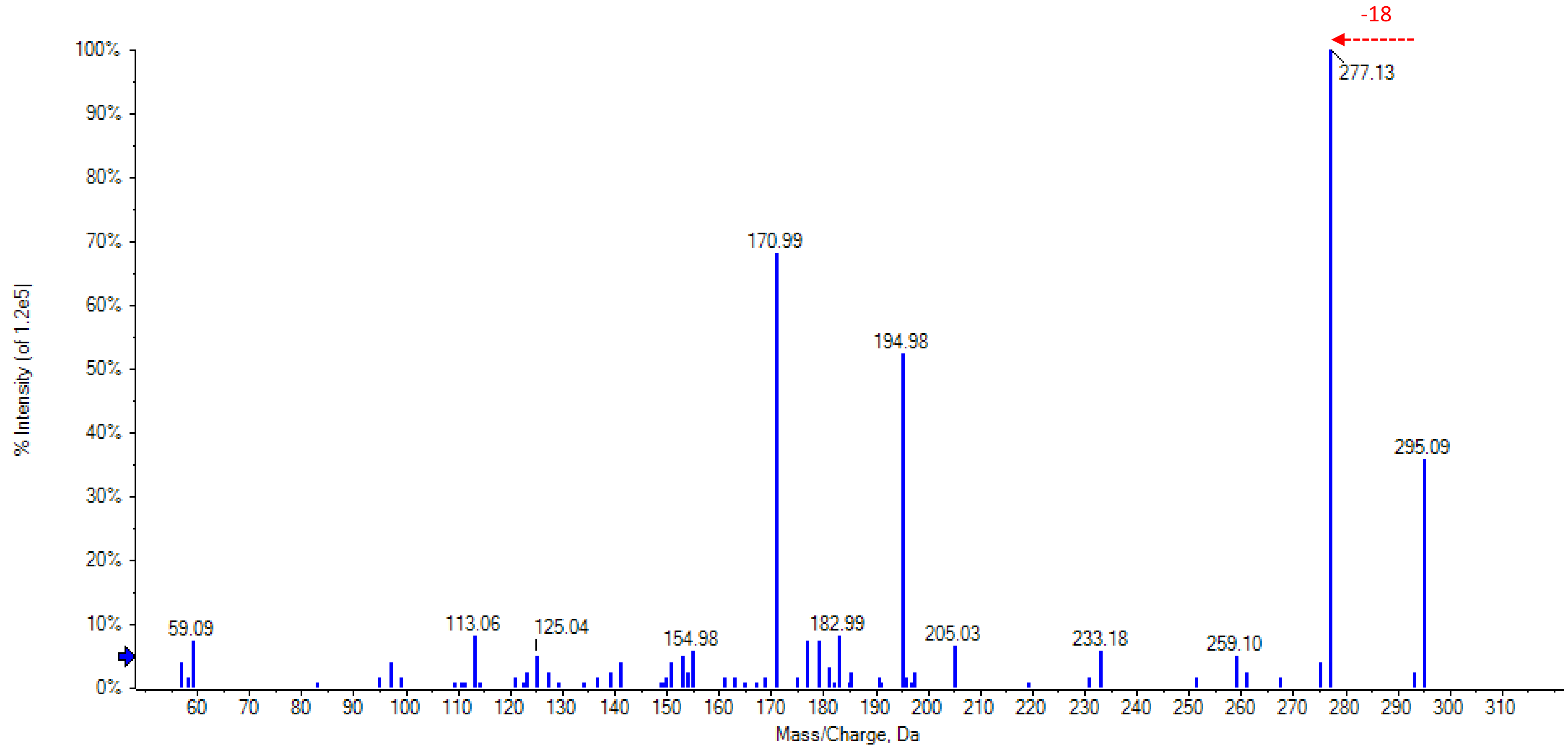

-18
277.13
170.99
194.98
295.09
59.09
113.06
125.04
154.98
182.99
205.03
233.18
259.10
% Intensity (of 1.2e5)
Mass/Charge, Da

oxoODE mix

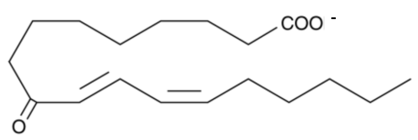

9-oxoODE

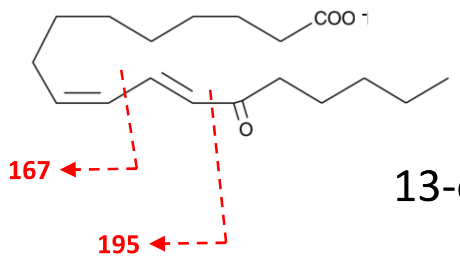

13-oxoODE

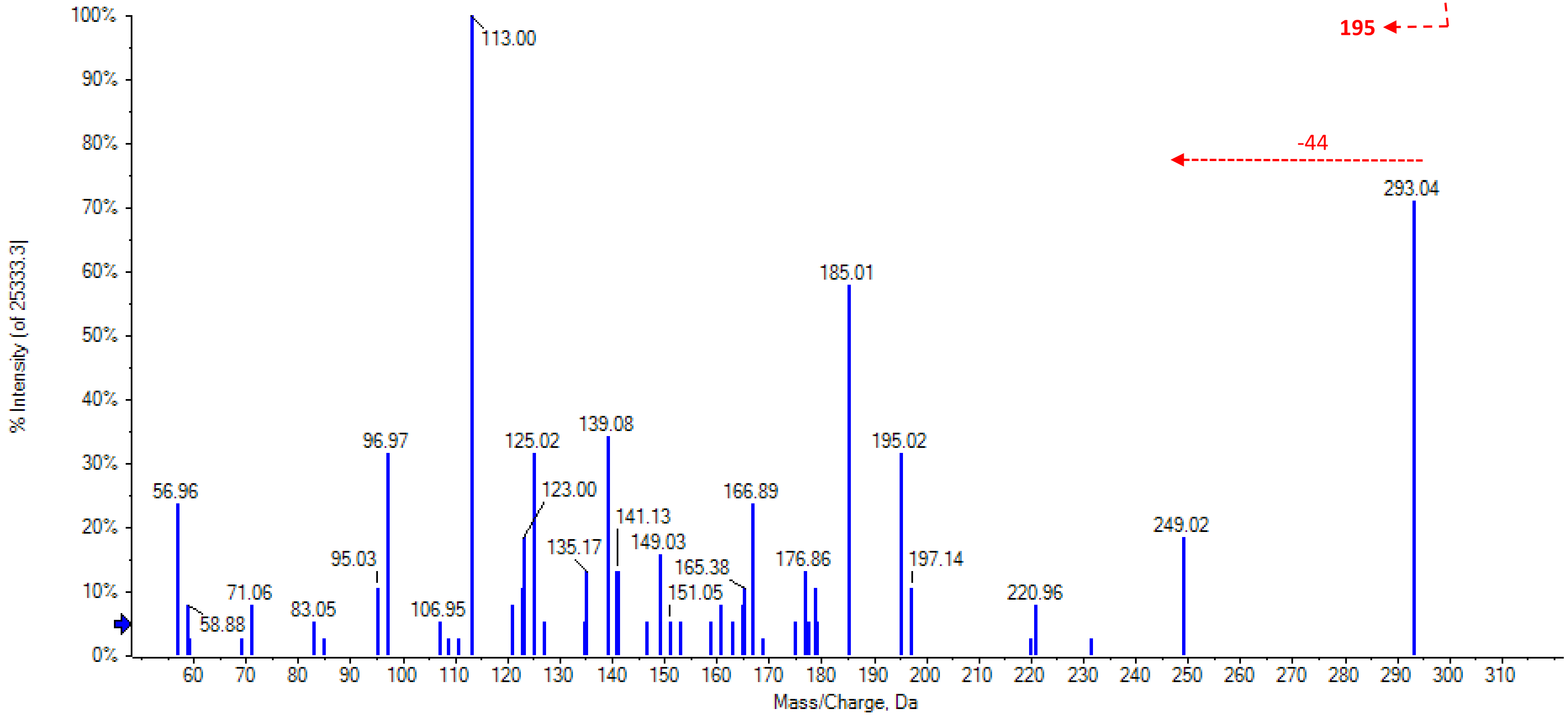

DiHOME mix

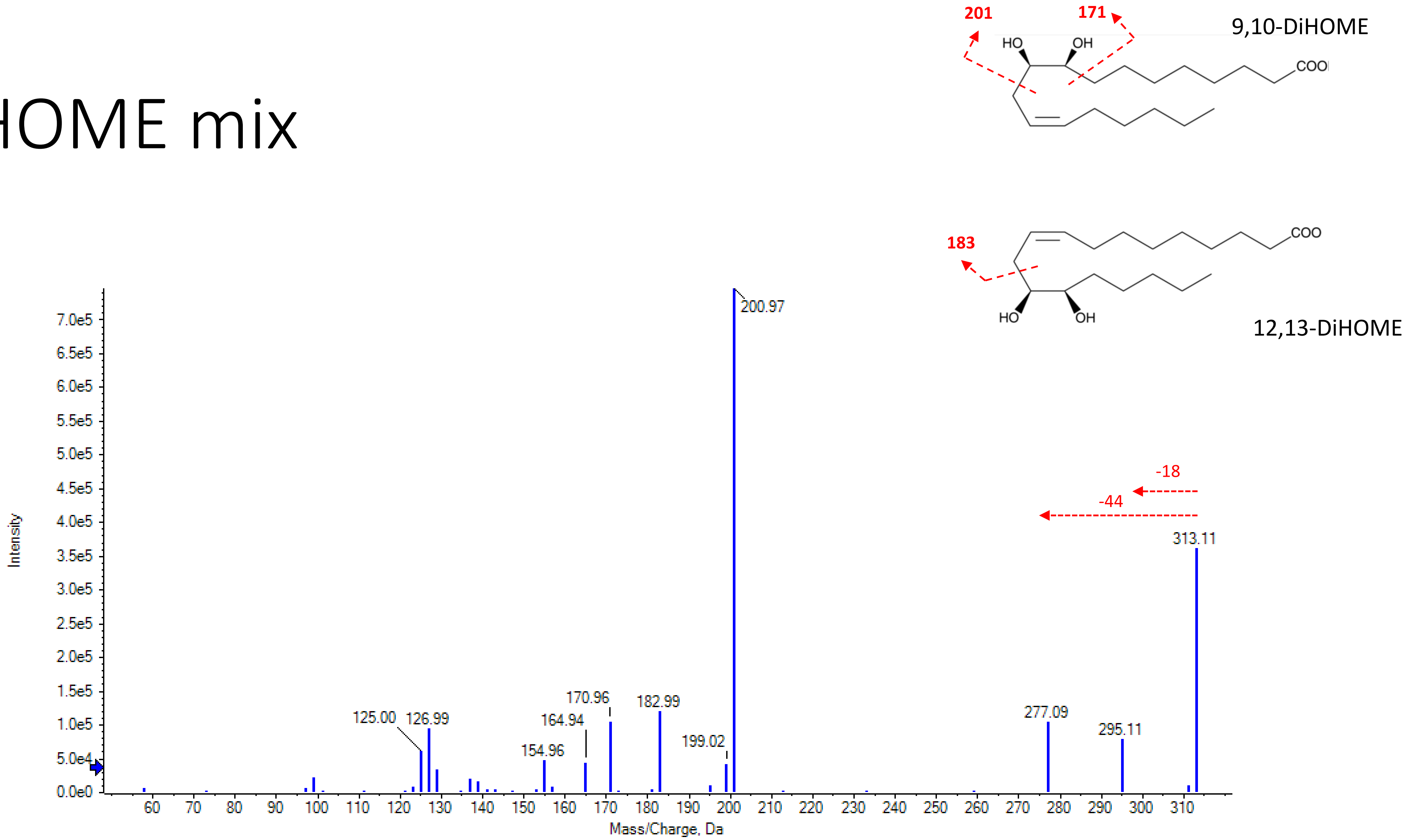

201
171
9,10-DiHOME
HO
OH
COO
183
COO
HO
OH
12,13-DiHOME
-18
-44
200.97
313.11
277.09
295.11
170.96
182.99
125.00
126.99
164.94
154.96
199.02
Intensity
7.0e5
6.5e5
6.0e5
5.5e5
5.0e5
4.5e5
4.0e5
3.5e5
3.0e5
2.5e5
2.0e5
1.5e5
1.0e5
5.0e4
0.0e0
60 70 80 90 100 110 120 130 140 150 160 170 180 190 200 210 220 230 240 250 260 270 280 290 300 310
Mass/Charge, Da

13-HODE-d4

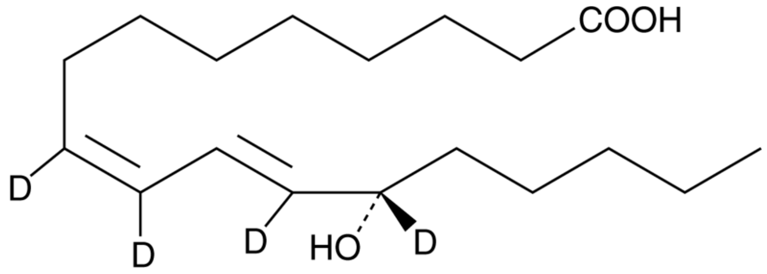

COOH
D
D
D
HO
D

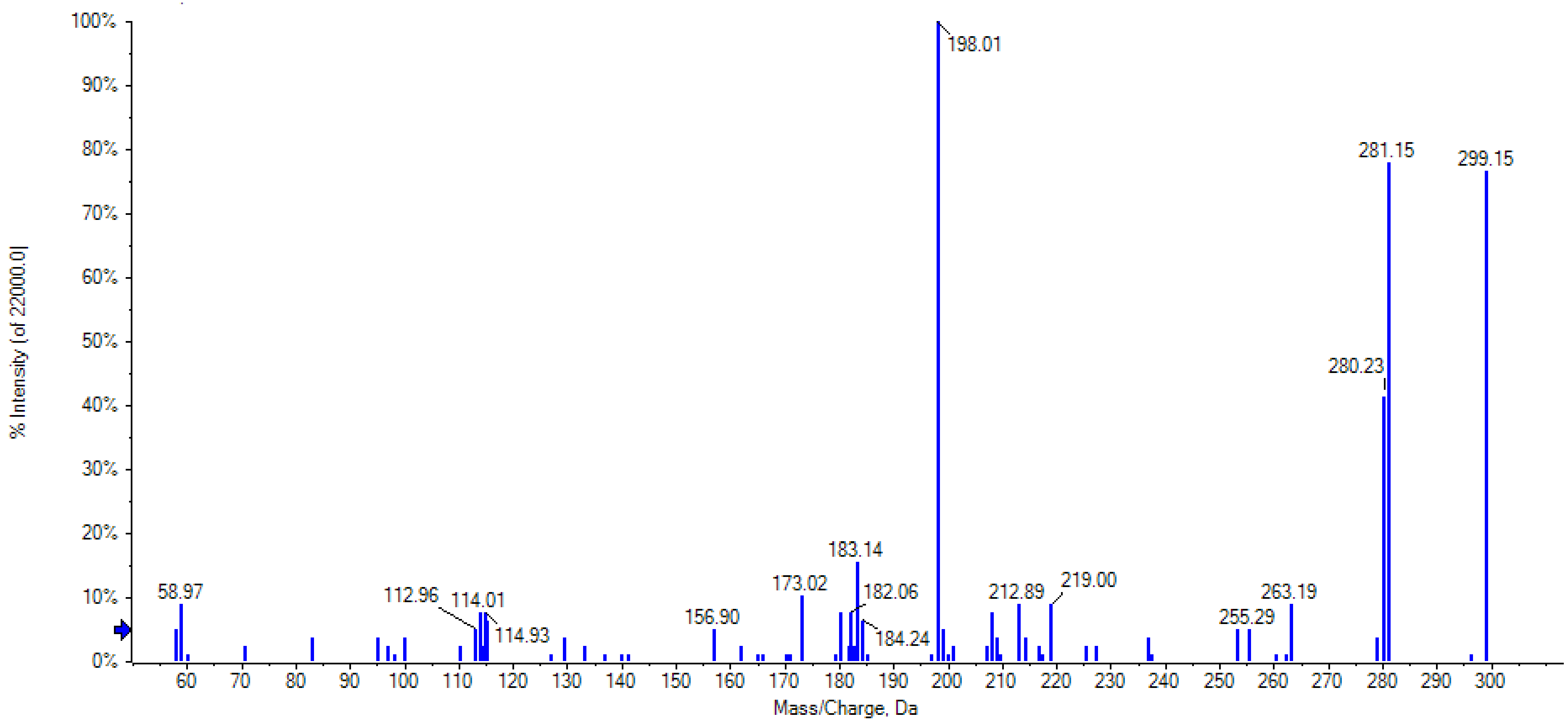

100%
90%
80%
70%
60%
50%
40%
30%
20%
10%
0%
% Intensity (of 22000.0)
198.01
281.15
299.15
280.23
183.14
173.02
182.06
219.00
212.89
263.19
58.97
112.96
114.01
114.93
156.90
184.24
255.29
60
70
80
90
100
110
120
130
140
150
160
170
180
190
200
210
220
230
240
250
260
270
280
290
300
Mass/Charge, Da

9-HODe-d4

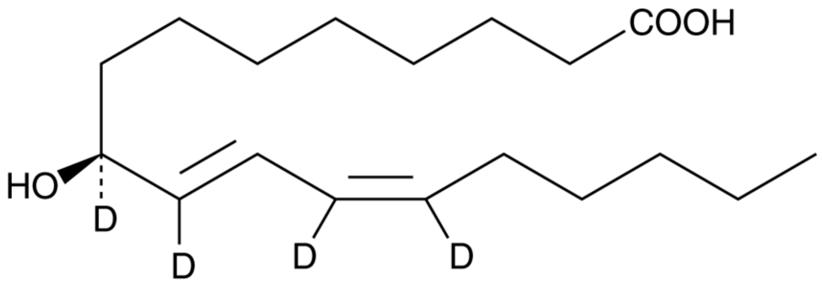
COOH
HO
D
D
D
D

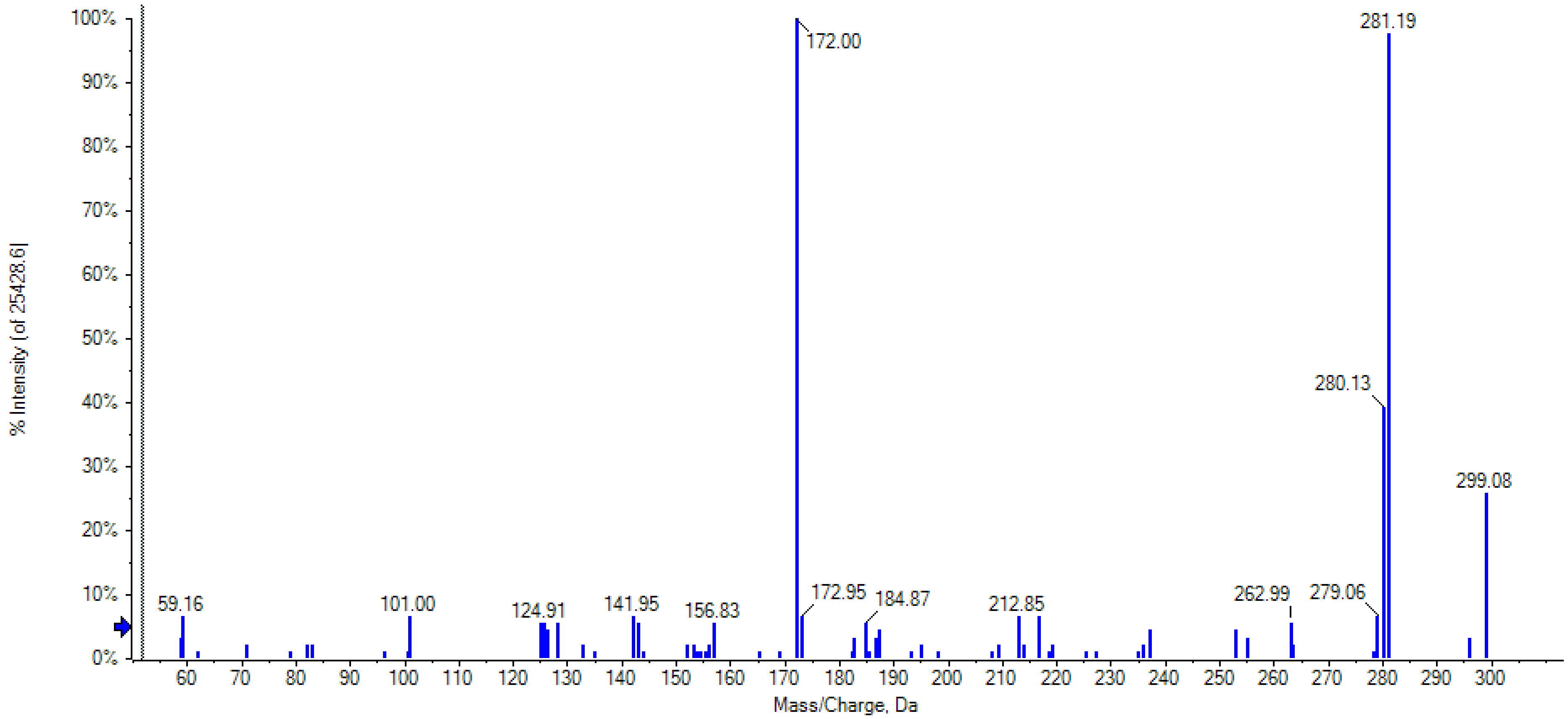
% Intensity (of 25428.6)
100%
90%
80%
70%
60%
50%
40%
30%
20%
10%
0%
172.00
281.19
280.13
299.08
59.16
101.00
124.91
141.95
156.83
172.95
184.87
212.85
262.99
279.06
60
70
80
90
100
110
120
130
140
150
160
170
180
190
200
210
220
230
240
250
260
270
280
290
300
Mass/Charge, Da

5-iso PGF2a (d11)

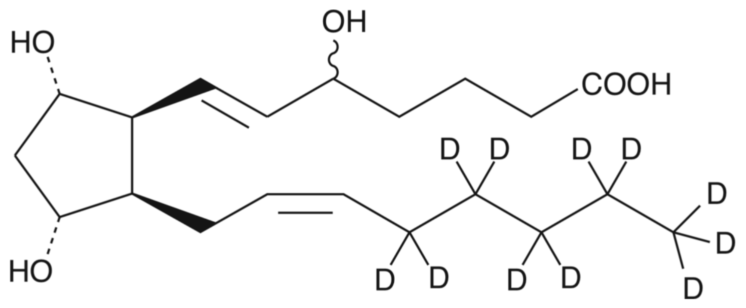

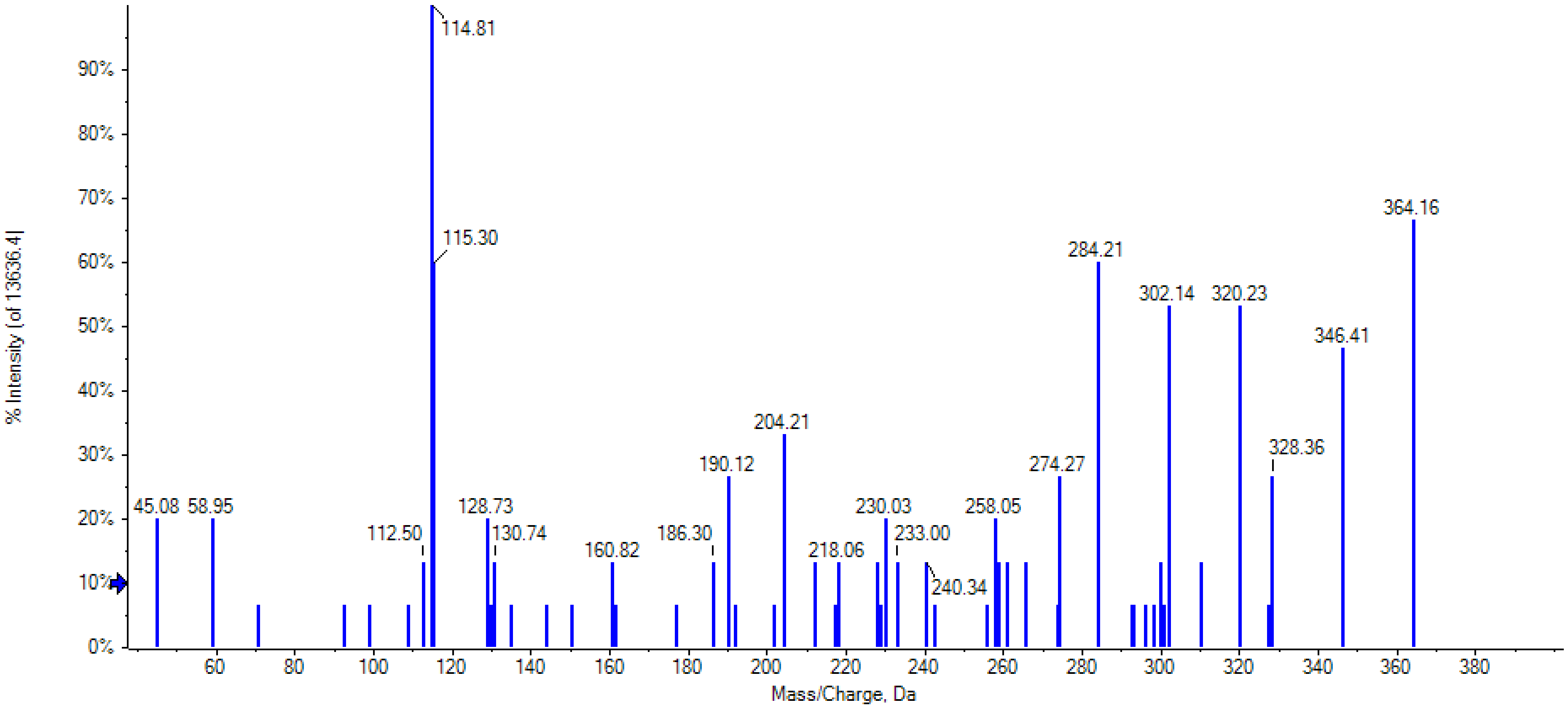

TxB2-d4

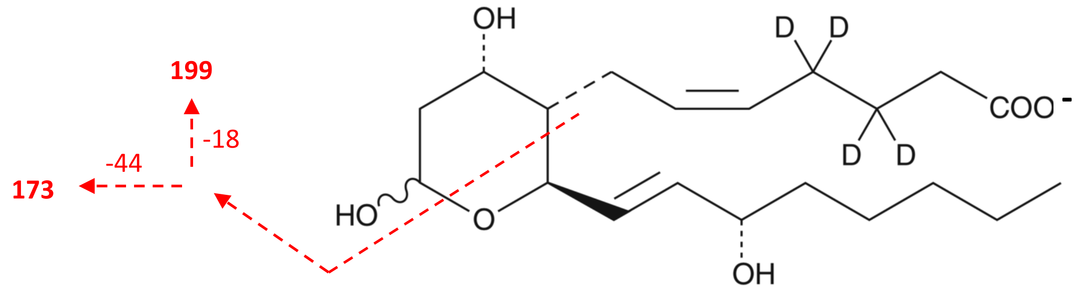
OH
D
D
COO-
D
D
199
-18
-44
173
HO
O
OH

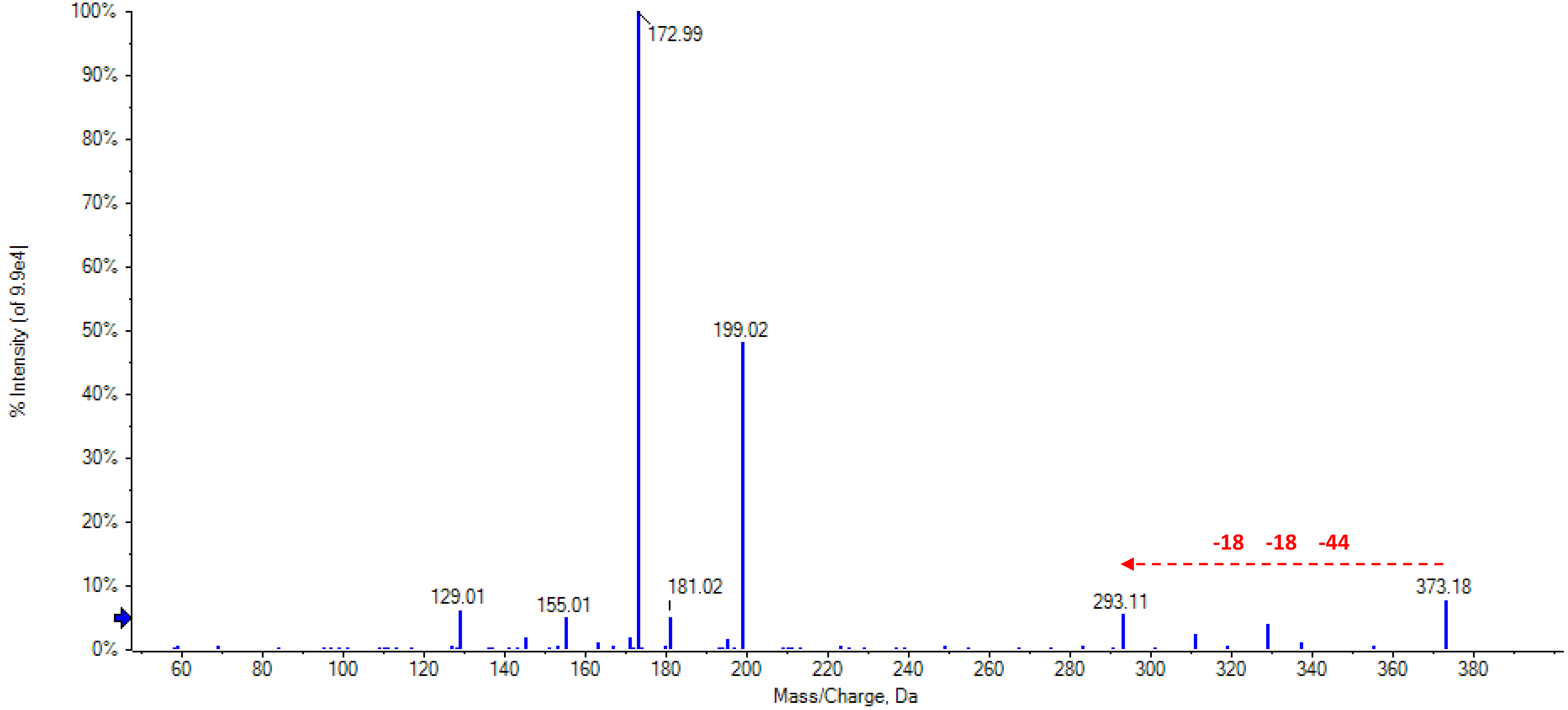
100%
90%
80%
70%
60%
50%
40%
30%
20%
10%
0%
% Intensity (of 9.9e4)
172.99
199.02
129.01
155.01
181.02
293.11
373.18
-18 -18 -44
60
80
100
120
140
160
180
200
220
240
260
280
300
320
340
360
380
Mass/Charge, Da

PGE2

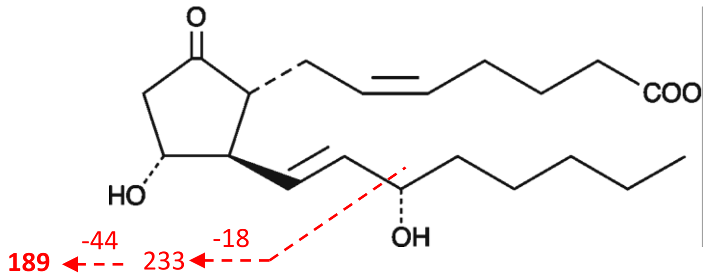

O
COO
HO
OH
189
-44
233
-18

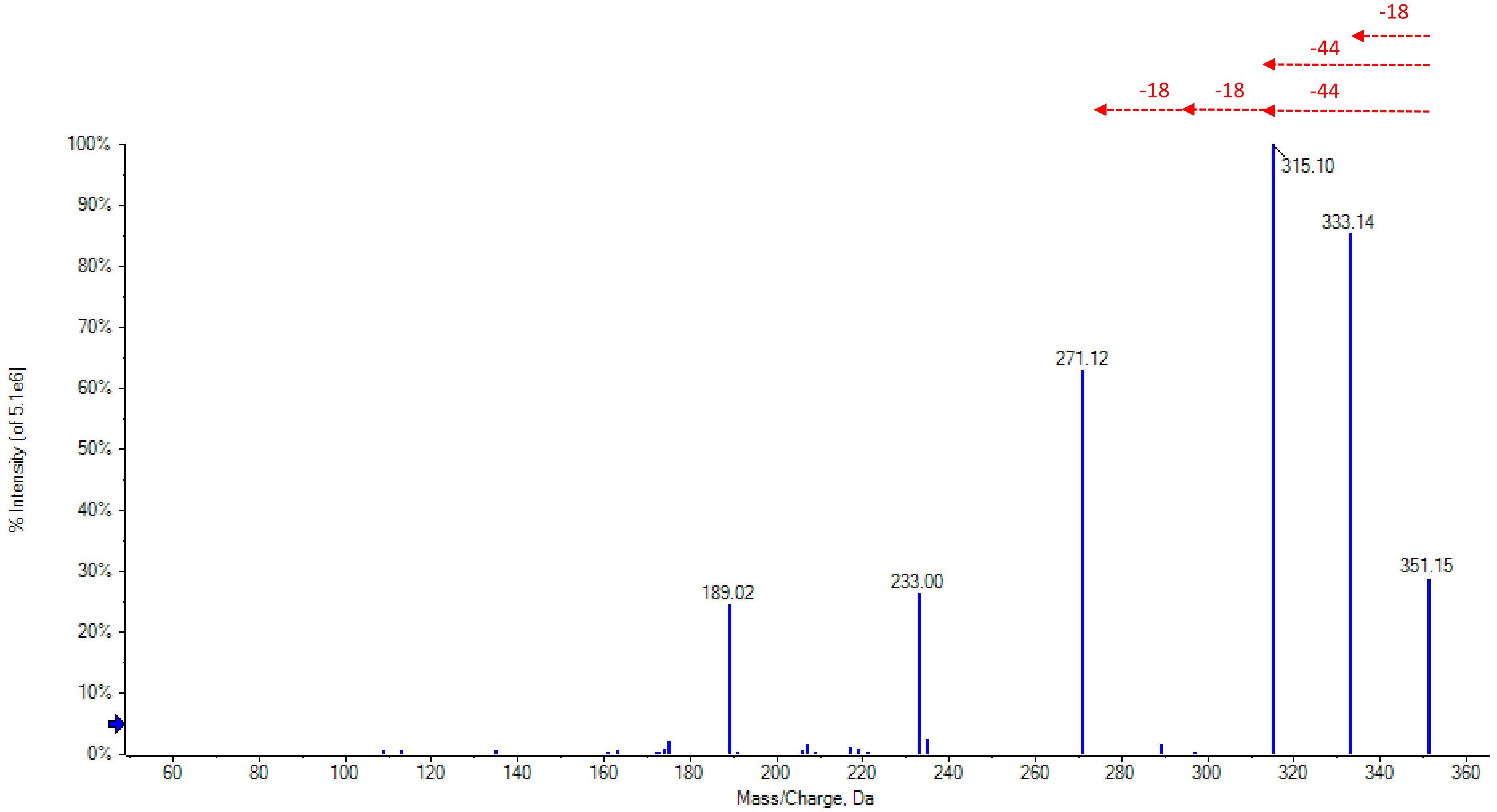

-18
-44
-18
-18
-44
315.10
333.14
271.12
351.15
233.00
189.02
% Intensity [of 5.1e6]
Mass/Charge, Da

PGE2-d4

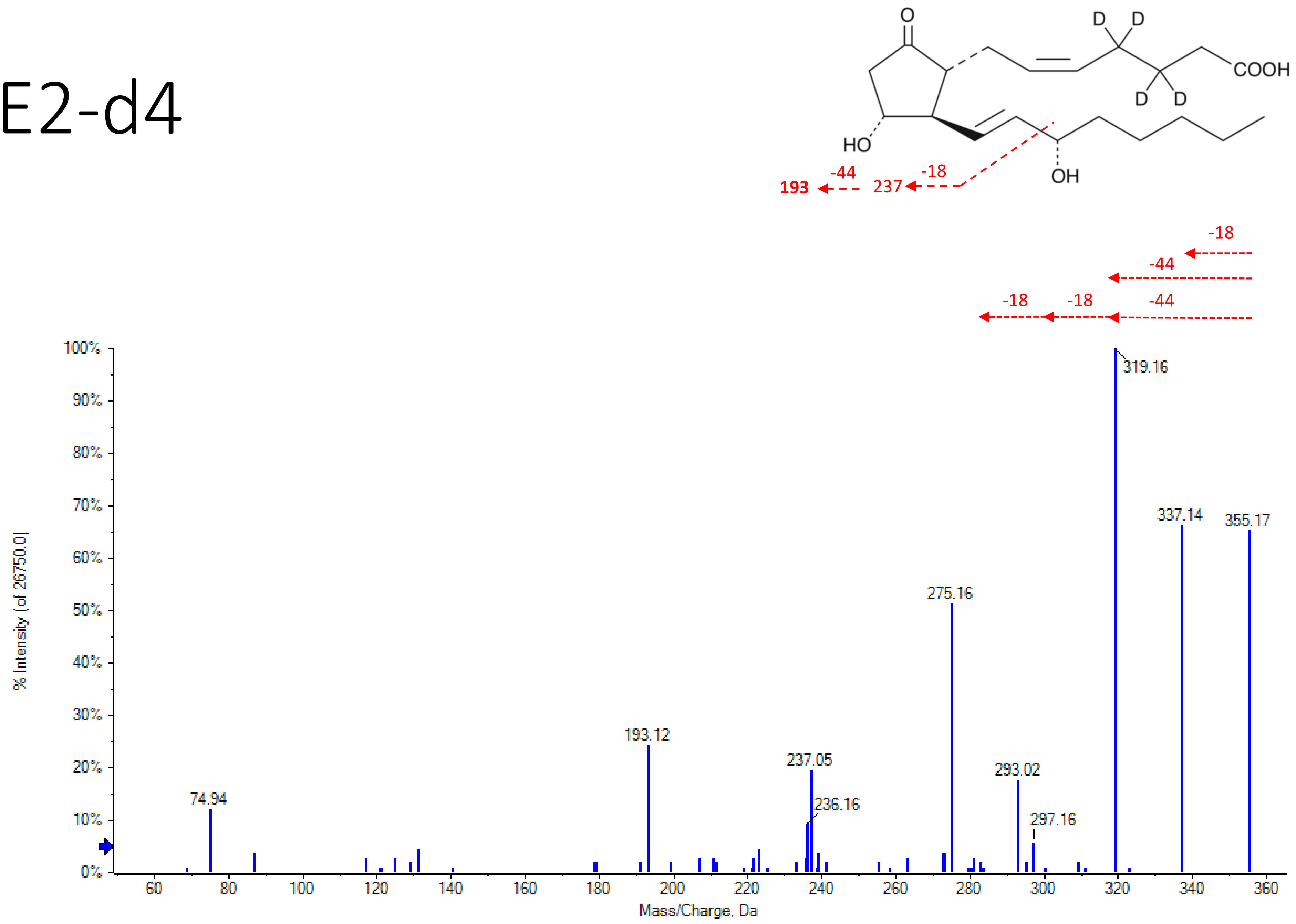
O
D D
COOH
D D
HO
OH
193
-44
237
-18
-18
-44
-18 -18 -44
100%
90%
80%
70%
60%
50%
40%
30%
20%
10%
0%
% Intensity (of 26750.0)
319.16
337.14
355.17
275.16
193.12
237.05
236.16
293.02
297.16
74.94
60 80 100 120 140 160 180 200 220 240 260 280 300 320 340 360
Mass/Charge, Da

PGD2-d4

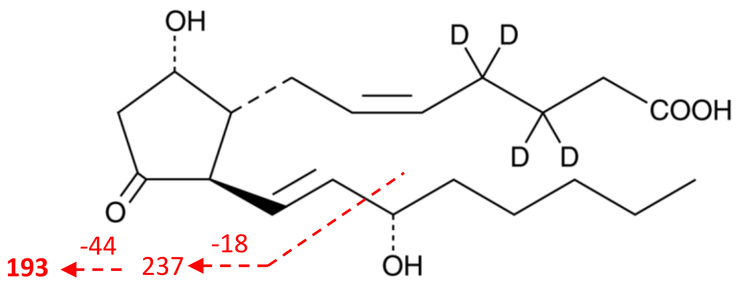
OH
D D
COOH
D D
O
OH
193 -44 237 -18

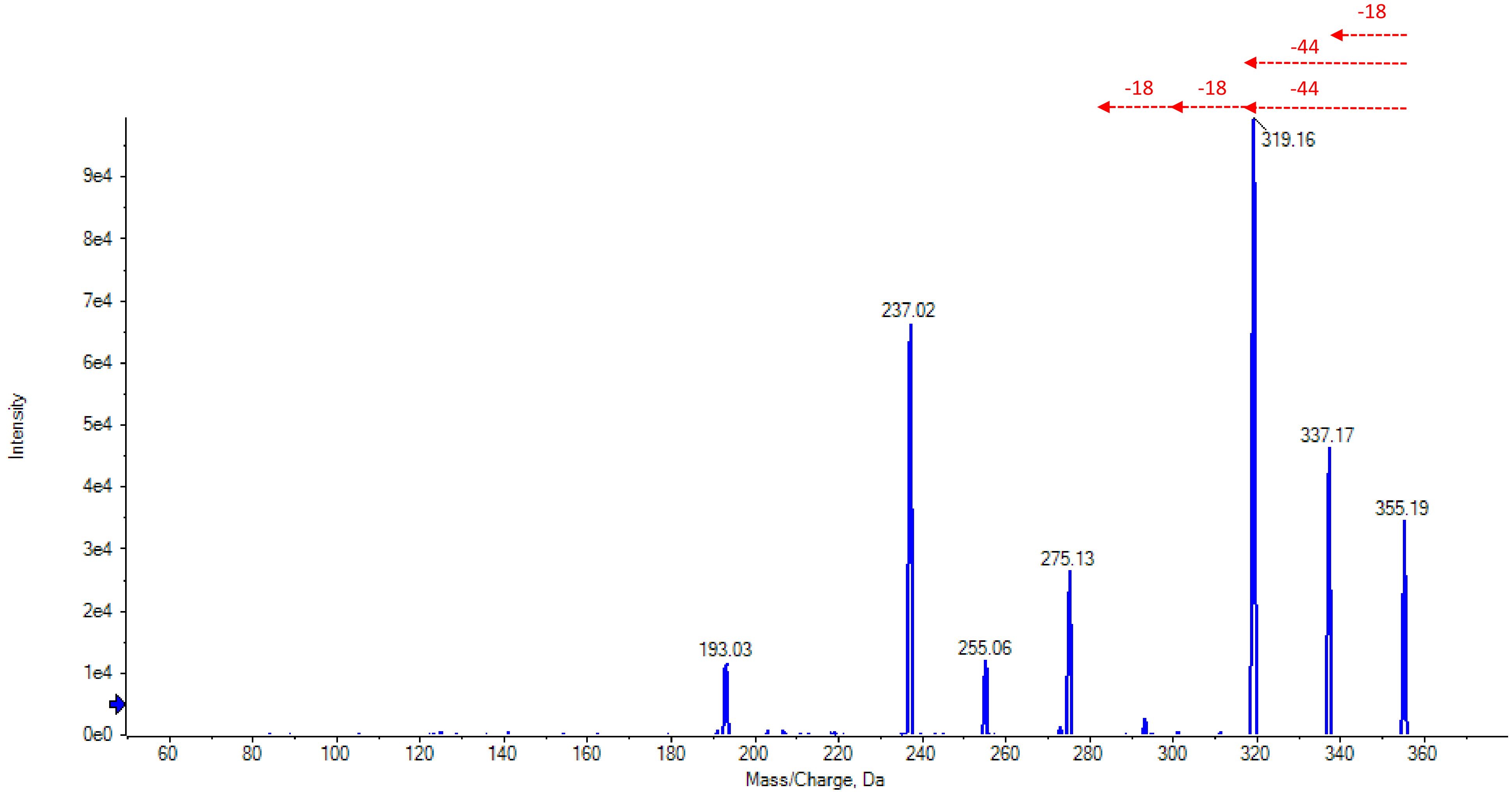
-18
-44
-18 -18 -44
319.16
237.02
337.17
355.19
275.13
193.03
255.06
Intensity
Mass/Charge, Da

PGF2α-d4

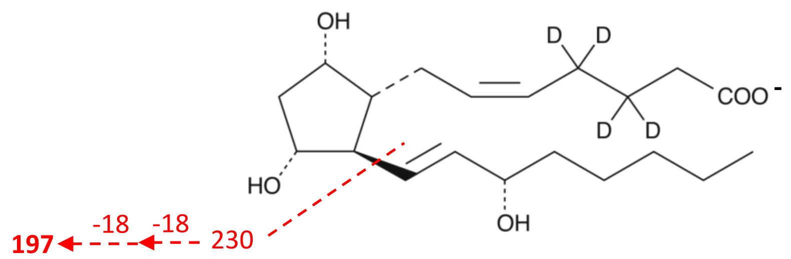

OH
D
D
COO-
D
D
HO
OH
197
-18
-18
230

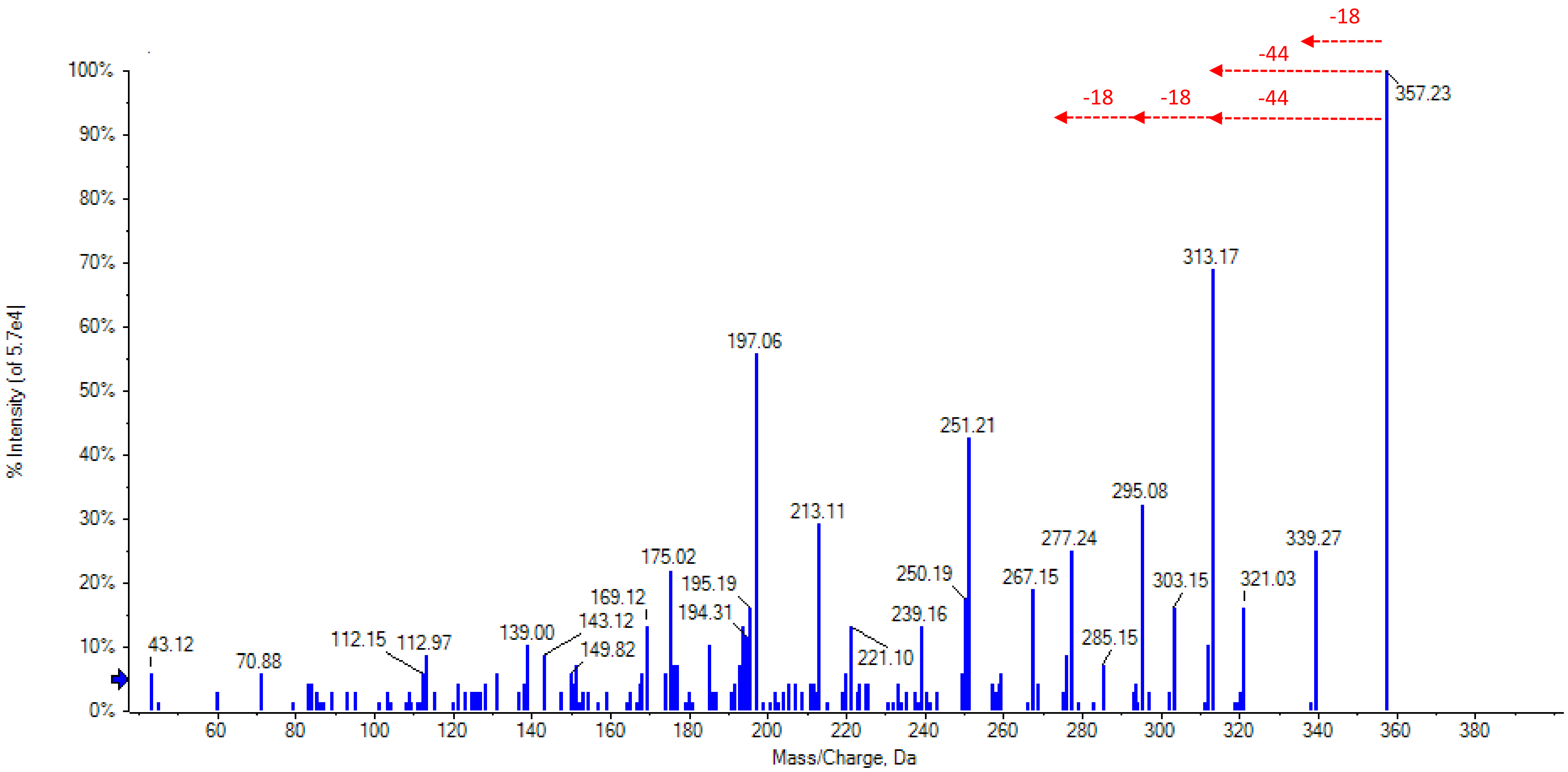

-18
-44
-18
-18
-44
357.23
313.17
197.06
251.21
295.08
213.11
277.24
339.27
175.02
195.19
250.19
267.15
303.15
321.03
169.12
194.31
239.16
143.12
285.15
112.15
112.97
139.00
149.82
221.10
43.12
70.88
100%
90%
80%
70%
60%
50%
40%
30%
20%
10%
0%
% Intensity (of 5.7e4)
60
80
100
120
140
160
180
200
220
240
260
280
300
320
340
360
380
Mass/Charge, Da

15-HETE

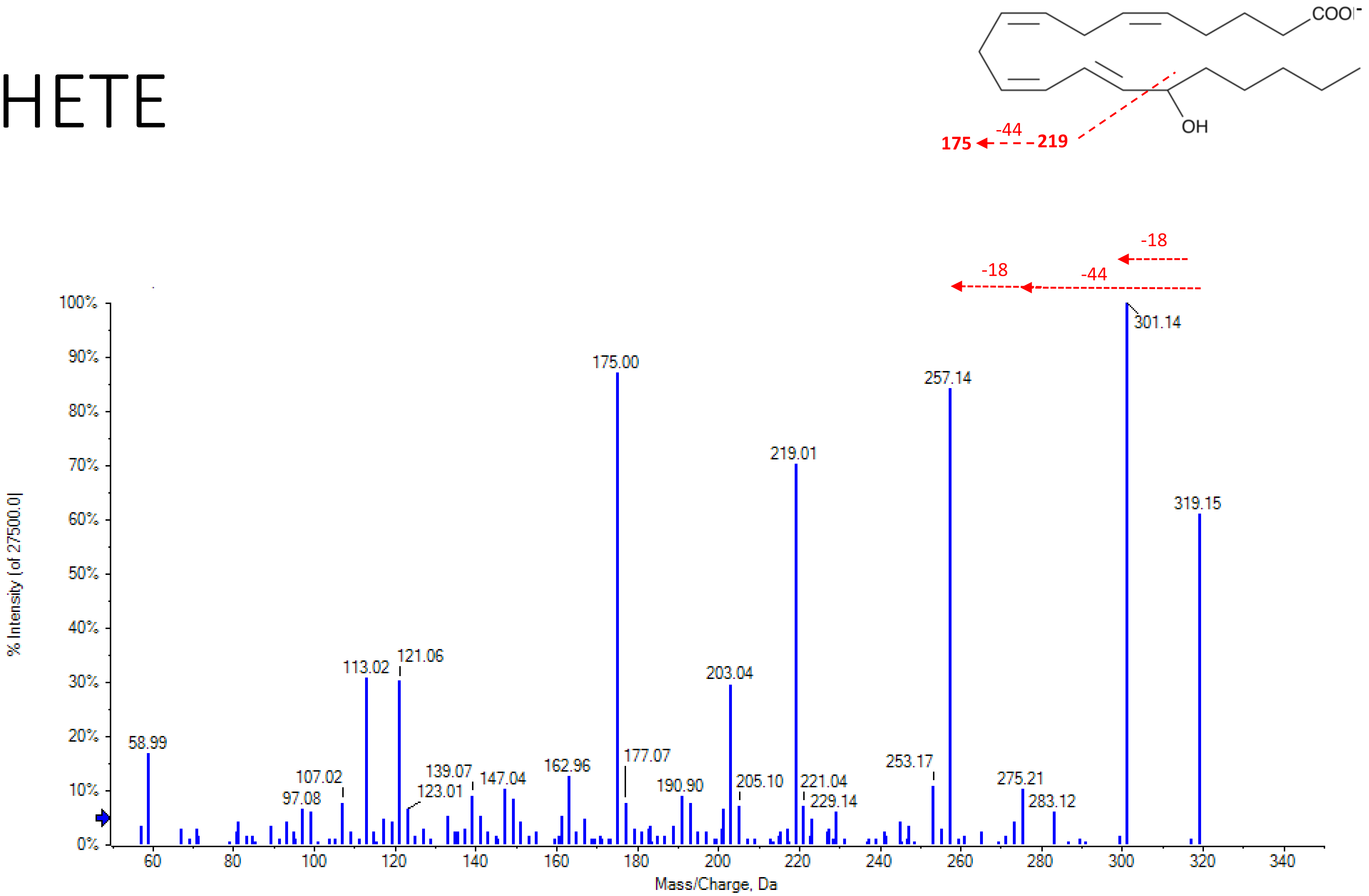
COO⁻
OH
175
-44
219
-18
-44
-18
100%
90%
80%
70%
60%
50%
40%
30%
20%
10%
0%
% Intensity (of 27500.0)
301.14
175.00
257.14
219.01
319.15
113.02
121.06
203.04
58.99
177.07
253.17
107.02
139.07
147.04
162.96
97.08
123.01
190.90
205.10
221.04
229.14
275.21
283.12
60
80
100
120
140
160
180
200
220
240
260
280
300
320
340
Mass/Charge, Da

12-HETE

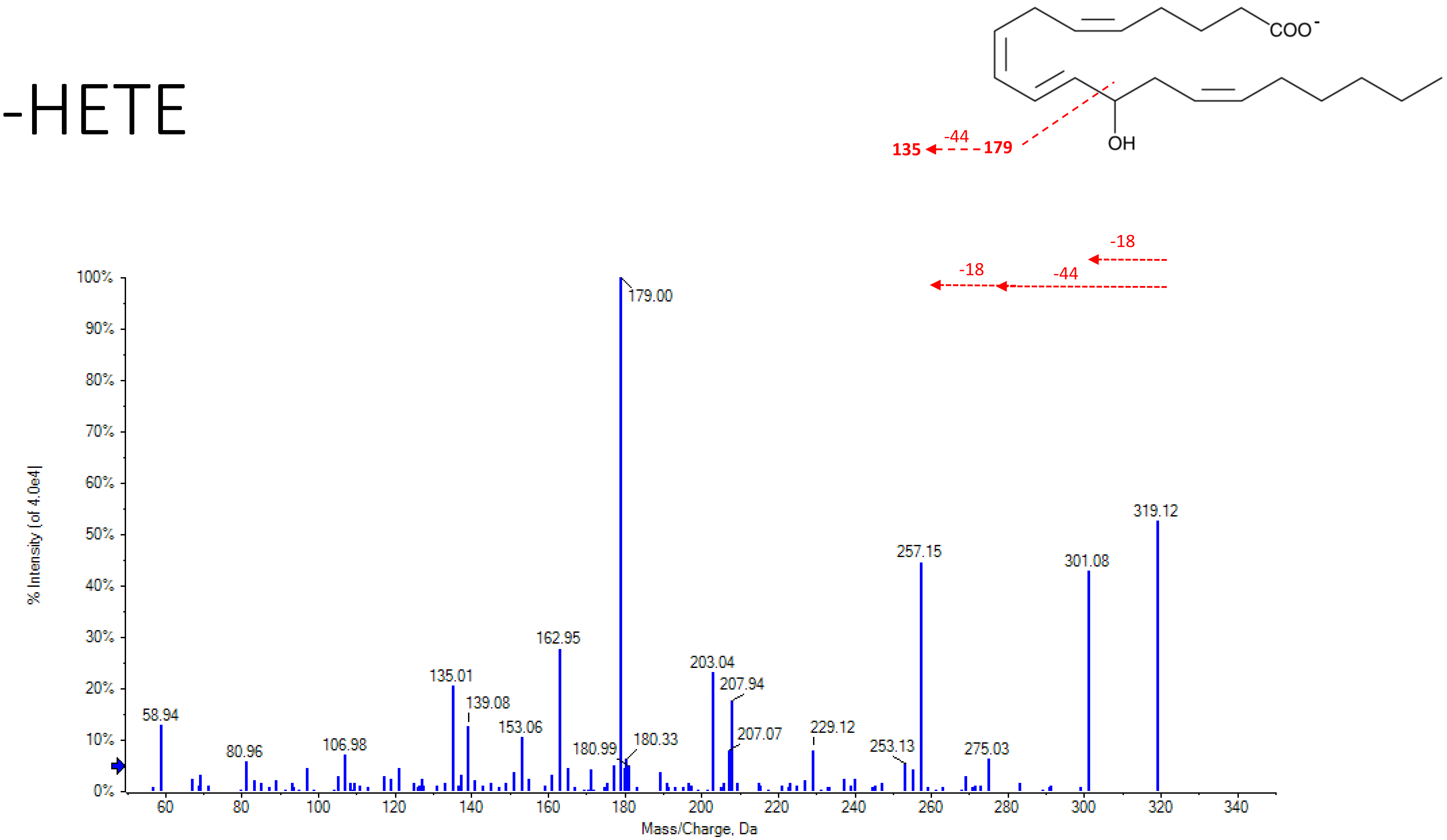

COO-
OH
135
-44
179
-18
-18
-44
100%
90%
80%
70%
60%
50%
40%
30%
20%
10%
0%
% Intensity (of 4.0e4)
179.00
319.12
257.15
301.08
162.95
203.04
207.94
135.01
139.08
153.06
58.94
207.07
229.12
180.33
180.99
106.98
80.96
253.13
275.03
60
80
100
120
140
160
180
200
220
240
260
280
300
320
340
Mass/Charge, Da

5-HETE

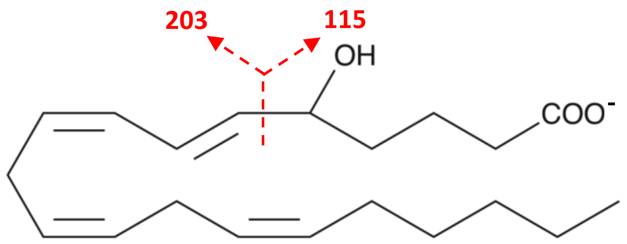
203
115
OH
COO-

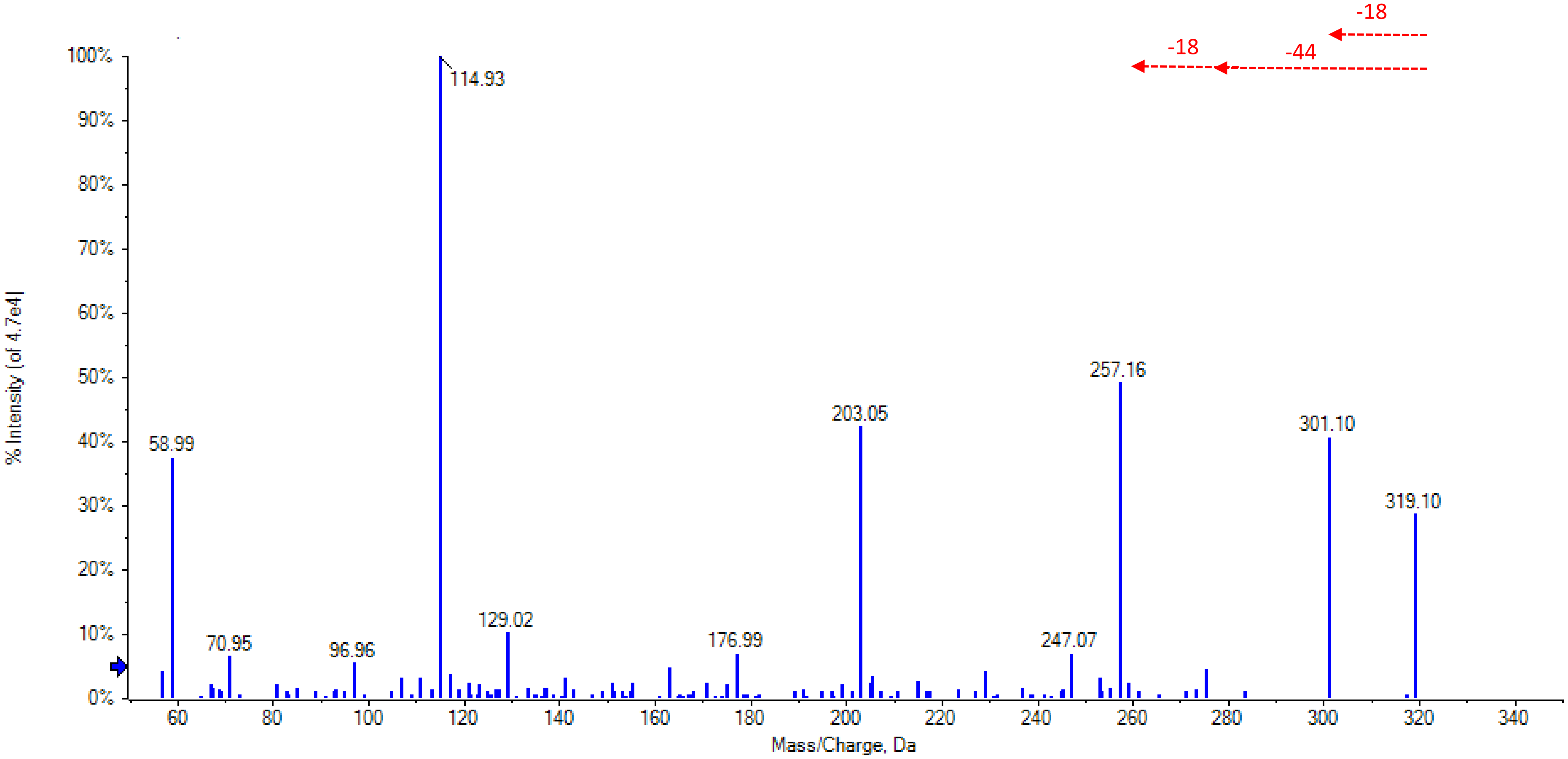
-18
-18
-44
114.93
58.99
70.95
96.96
129.02
176.99
203.05
247.07
257.16
301.10
319.10
% Intensity (of 4.7e4)
Mass/Charge, Da

Lipoxin mix

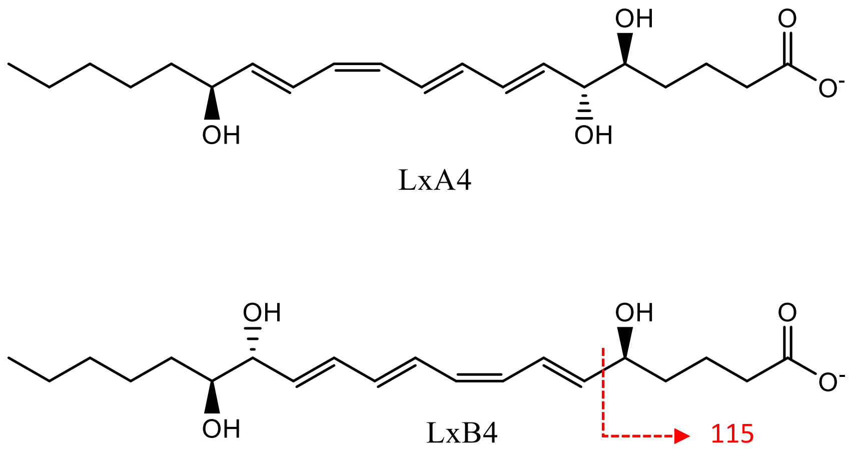
OH
O
O-
OH
OH
LxA4
OH
OH
O
O-
OH
LxB4
115

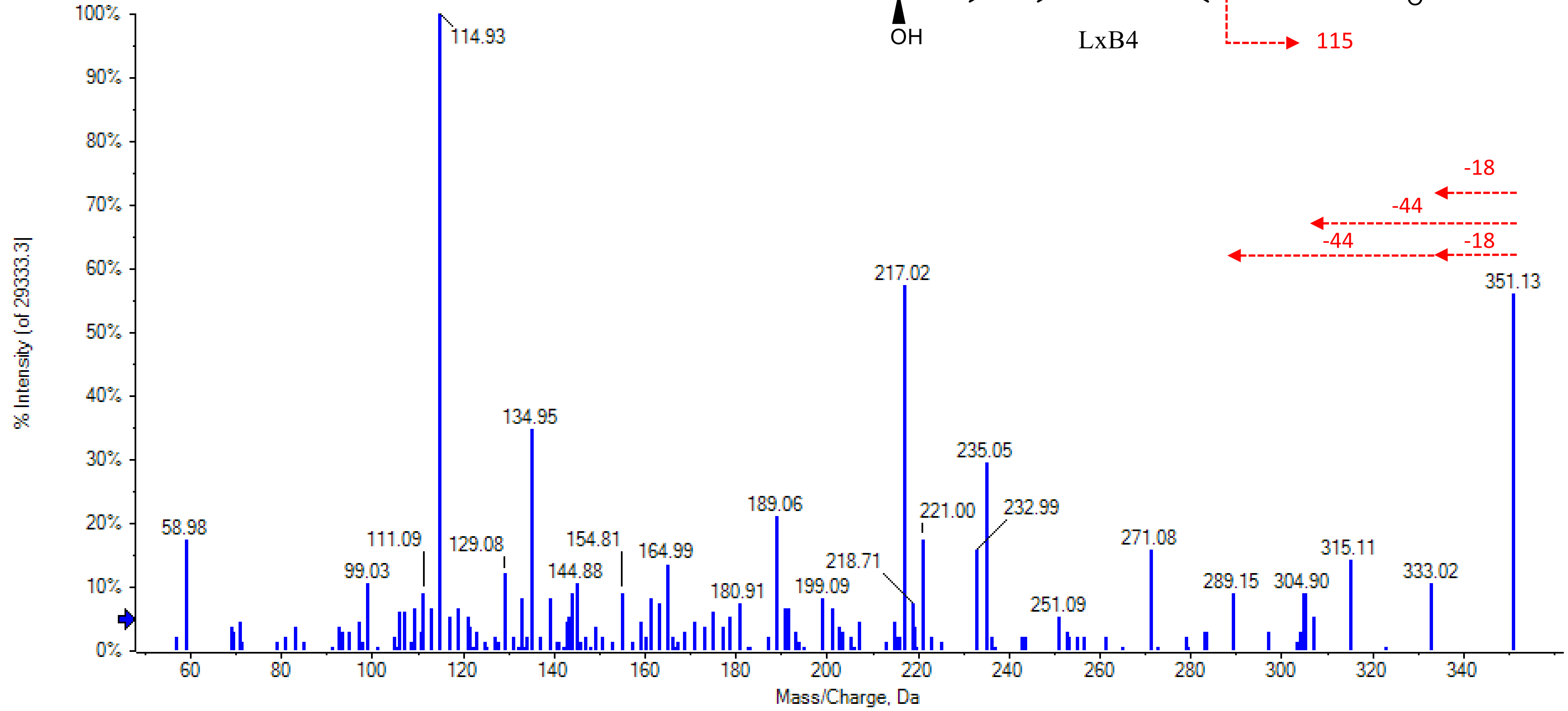
-18
-44
-44
-18
100%
90%
80%
70%
60%
50%
40%
30%
20%
10%
0%
% Intensity [of 29333.3]
114.93
134.95
217.02
351.13
235.05
232.99
221.00
189.06
58.98
111.09
129.08
154.81
164.99
99.03
144.88
218.71
180.91
199.09
271.08
315.11
333.02
289.15
304.90
251.09
60
80
100
120
140
160
180
200
220
240
260
280
300
320
340
Mass/Charge, Da
