# Supplementary material for: HPLC-MS/MS Oxylipin Analysis of Plasma from Amyotrophic Lateral Sclerosis Patients
Source: Biomedicines. 2022 Mar 15;10(3):674. doi: 10.3390/biomedicines10030674 (PMC8945419; doi:10.3390/biomedicines10030674)

Figure S2. Chromatographic separation of linoleic (LA), arachidonic (AA), eicosapentenoic (EPA) and docosahexenoic (DHA) acids derived oxylipins. Separation of the standard mix was performed as described in the *Material and Methods* section. Chromatograms shown in figure correspond to the most intense transition for each standard.

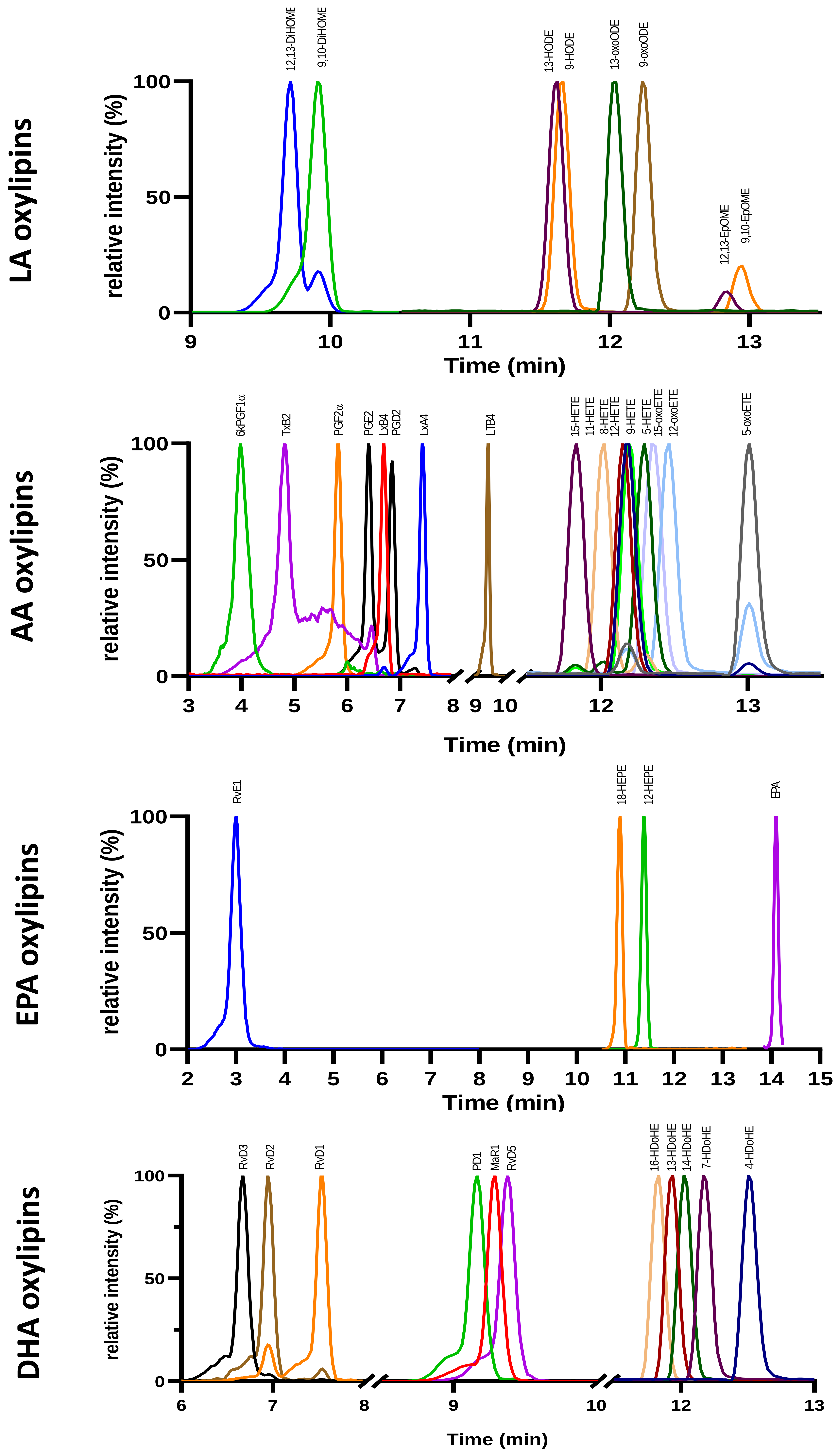

Supplement: Supplementary file 1 [file biomedicines-10-00674-s001.zip › Figure S2- Chormatographic separation.pdf]
