# Supplementary material for: HPLC-MS/MS Oxylipin Analysis of Plasma from Amyotrophic Lateral Sclerosis Patients
Source: Biomedicines. 2022 Mar 15;10(3):674. doi: 10.3390/biomedicines10030674 (PMC8945419; doi:10.3390/biomedicines10030674)
Supplement: Supplementary file 1 [file biomedicines-10-00674-s001.zip › Figure S3 - LOD & LOQ.pdf]

Figure S3. Limit of detection and limit of quantitation for SPM and related metabolites. A standard mix containing all SPM available in our lab at a low concentration was diluted with methanol several times before internal standard mix addition and HPLC-MS/MS analysis. Signal finder algorithm (Multiquant 2.1, ABSciex) was employed for peak area integration of the most intense transition for each SPM. Then, LOD and LOQ were defined as the mass (pg) at which the signal-to-ratio (S/N) was equal or greater than 3 or 5, respectively. Each figure show chromatograms obtained for the most intense transition for each analyte at different concentration (expressed as pg of analyte injected on column).

RvE1

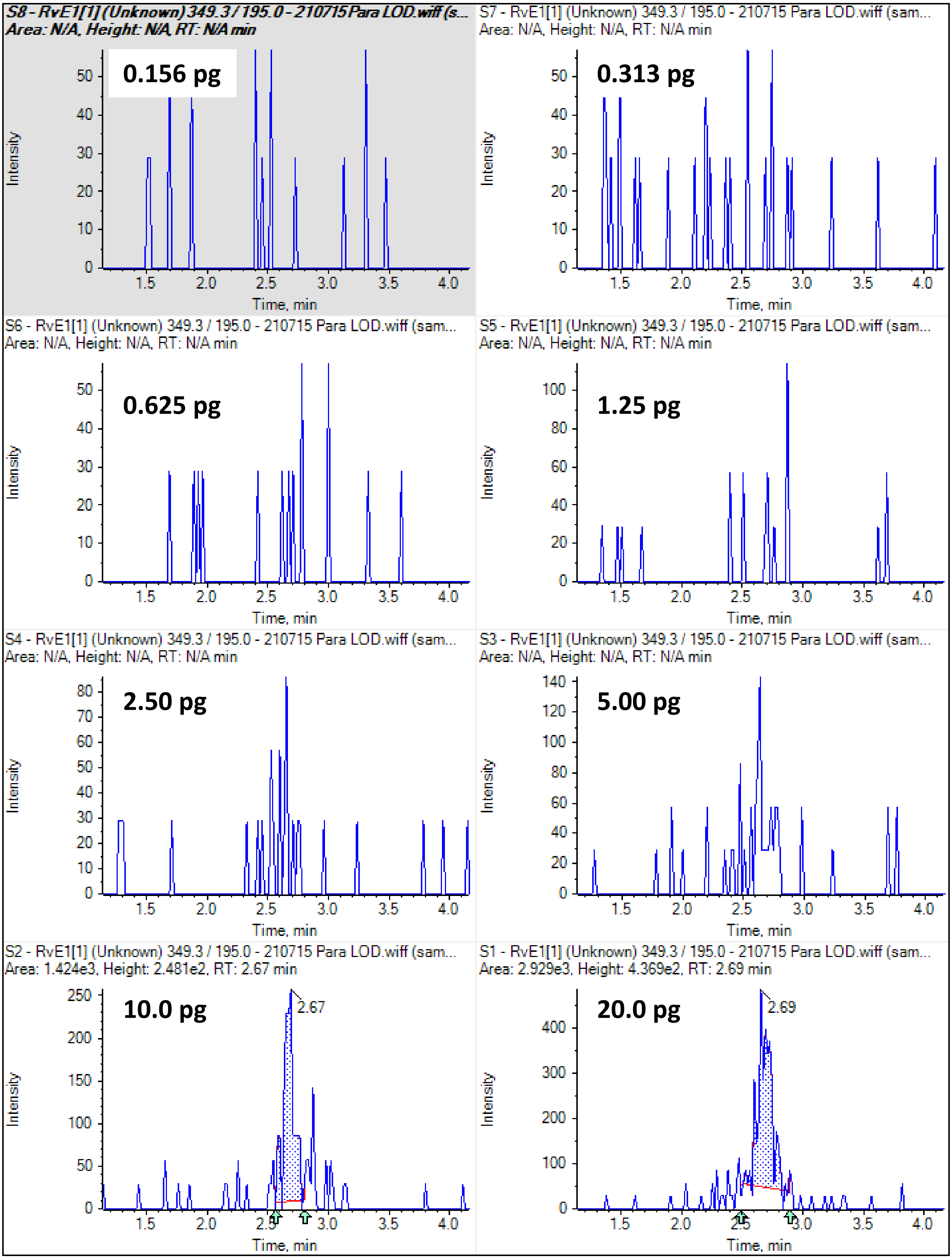
S8 - RvE1[1] (Unknown) 349.3 / 195.0 - 210715 Para LOD.wiff (s...
Area: N/A, Height: N/A, RT: N/A min
0.156 pg
S7 - RvE1[1] (Unknown) 349.3 / 195.0 - 210715 Para LOD.wiff (sam...
Area: N/A, Height: N/A, RT: N/A min
0.313 pg
S6 - RvE1[1] (Unknown) 349.3 / 195.0 - 210715 Para LOD.wiff (sam...
Area: N/A, Height: N/A, RT: N/A min
0.625 pg
S5 - RvE1[1] (Unknown) 349.3 / 195.0 - 210715 Para LOD.wiff (sam...
Area: N/A, Height: N/A, RT: N/A min
1.25 pg
S4 - RvE1[1] (Unknown) 349.3 / 195.0 - 210715 Para LOD.wiff (sam...
Area: N/A, Height: N/A, RT: N/A min
2.50 pg
S3 - RvE1[1] (Unknown) 349.3 / 195.0 - 210715 Para LOD.wiff (sam...
Area: N/A, Height: N/A, RT: N/A min
5.00 pg
S2 - RvE1[1] (Unknown) 349.3 / 195.0 - 210715 Para LOD.wiff (sam...
Area: 1.424e3, Height: 2.481e2, RT: 2.67 min
10.0 pg
2.67
S1 - RvE1[1] (Unknown) 349.3 / 195.0 - 210715 Para LOD.wiff (sam...
Area: 2.929e3, Height: 4.369e2, RT: 2.69 min
20.0 pg
2.69
Intensity
Time, min

TxB2

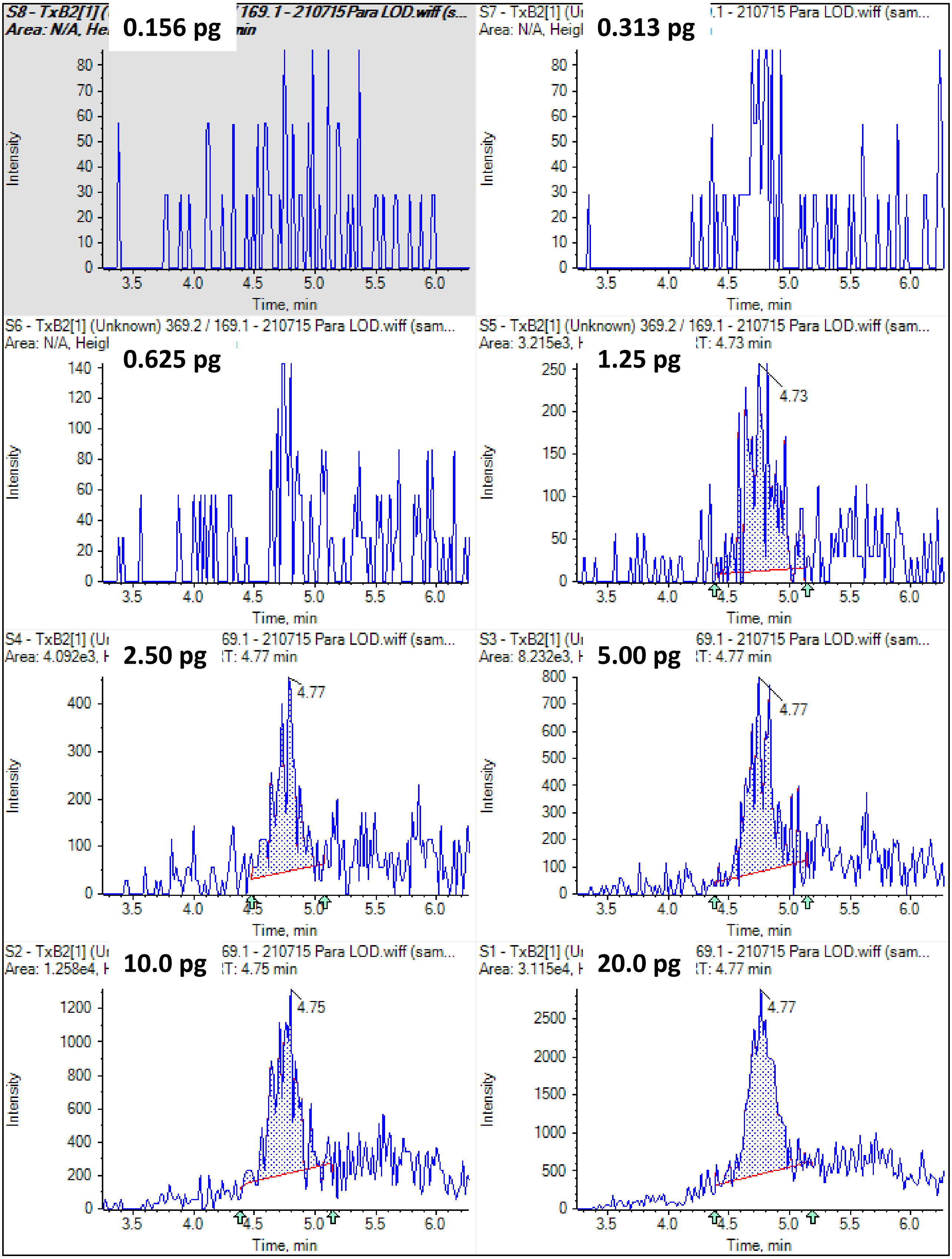

S8 - TxB2[1] (
Area: N/A, He
0.156 pg
169.1 - 210715 Para LOD.wiff (s...
S7 - TxB2[1] (U
Area: N/A, Heigl
0.313 pg
.1 - 210715 Para LOD.wiff (sam...
S6 - TxB2[1] (Unknown) 369.2 / 169.1 - 210715 Para LOD.wiff (sam...
Area: N/A, Heigl
0.625 pg
S5 - TxB2[1] (Unknown) 369.2 / 169.1 - 210715 Para LOD.wiff (sam...
Area: 3.215e3, I
1.25 pg
T: 4.73 min
4.73
S4 - TxB2[1] (U
Area: 4.092e3, I
2.50 pg
69.1 - 210715 Para LOD.wiff (sam...
T: 4.77 min
4.77
S3 - TxB2[1] (U
Area: 8.232e3, I
5.00 pg
69.1 - 210715 Para LOD.wiff (sam...
T: 4.77 min
4.77
S2 - TxB2[1] (U
Area: 1.258e4, I
10.0 pg
69.1 - 210715 Para LOD.wiff (sam...
T: 4.75 min
4.75
S1 - TxB2[1] (U
Area: 3.115e4, I
20.0 pg
69.1 - 210715 Para LOD.wiff (sam...
T: 4.77 min
4.77
Intensity
Time, min

PGF2α

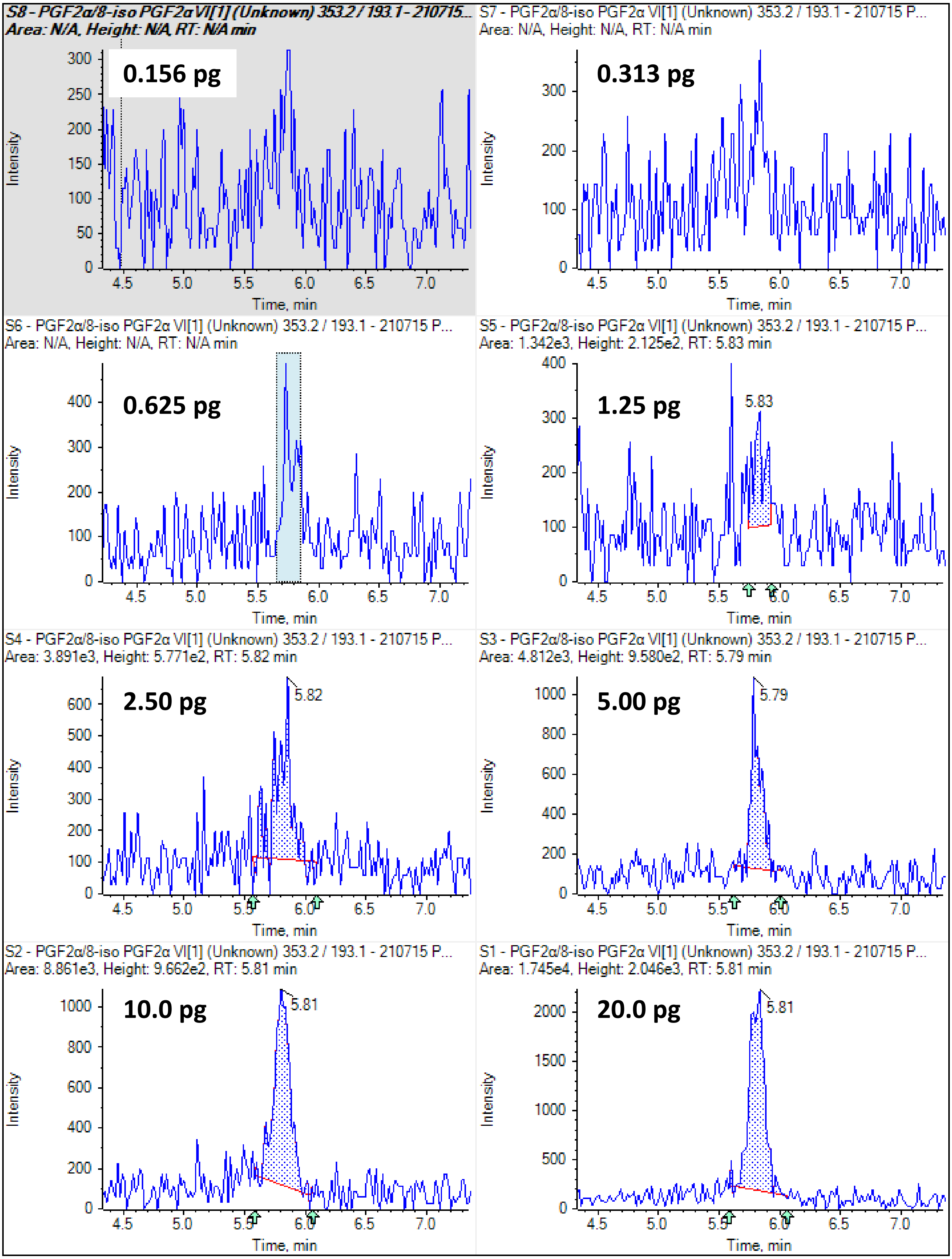

S8 - PGF2α/8-iso PGF2α VI[1] (Unknown) 353.2 / 193.1 - 210715...
Area: N/A, Height: N/A, RT: N/A min
0.156 pg
S7 - PGF2α/8-iso PGF2α VI[1] (Unknown) 353.2 / 193.1 - 210715 P...
Area: N/A, Height: N/A, RT: N/A min
0.313 pg
S6 - PGF2α/8-iso PGF2α VI[1] (Unknown) 353.2 / 193.1 - 210715 P...
Area: N/A, Height: N/A, RT: N/A min
0.625 pg
S5 - PGF2α/8-iso PGF2α VI[1] (Unknown) 353.2 / 193.1 - 210715 P...
Area: 1.342e3, Height: 2.125e2, RT: 5.83 min
1.25 pg
5.83
S4 - PGF2α/8-iso PGF2α VI[1] (Unknown) 353.2 / 193.1 - 210715 P...
Area: 3.891e3, Height: 5.771e2, RT: 5.82 min
2.50 pg
5.82
S3 - PGF2α/8-iso PGF2α VI[1] (Unknown) 353.2 / 193.1 - 210715 P...
Area: 4.812e3, Height: 9.580e2, RT: 5.79 min
5.00 pg
5.79
S2 - PGF2α/8-iso PGF2α VI[1] (Unknown) 353.2 / 193.1 - 210715 P...
Area: 8.861e3, Height: 9.662e2, RT: 5.81 min
10.0 pg
5.81
S1 - PGF2α/8-iso PGF2α VI[1] (Unknown) 353.2 / 193.1 - 210715 P...
Area: 1.745e4, Height: 2.046e3, RT: 5.81 min
20.0 pg
5.81
Intensity
Time, min

PGE2

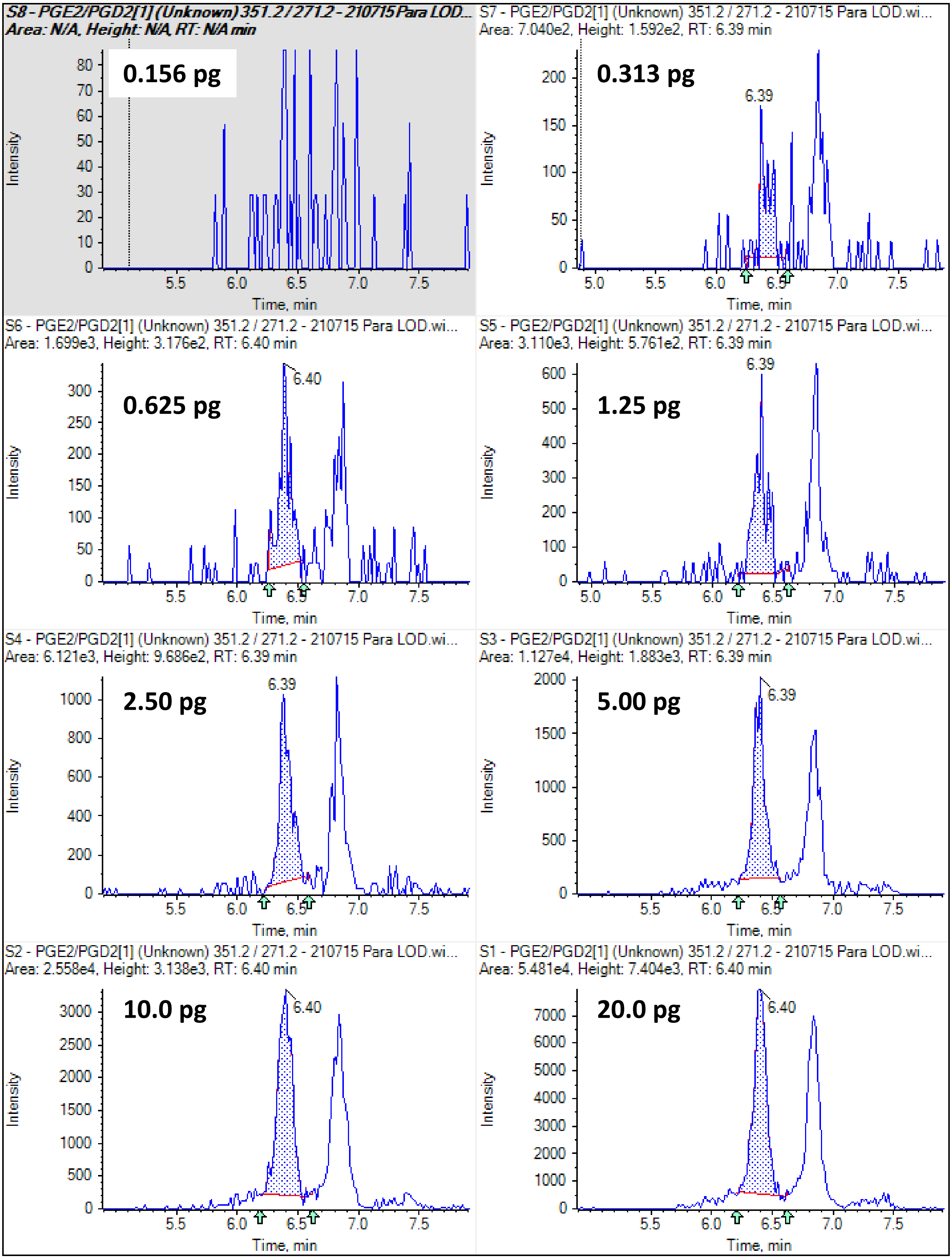

S8 - PGE2/PGD2[1] (Unknown) 351.2 / 271.2 - 210715 Para LOD...
Area: N/A, Height: N/A, RT: N/A min
0.156 pg
S7 - PGE2/PGD2[1] (Unknown) 351.2 / 271.2 - 210715 Para LOD.wi...
Area: 7.040e2, Height: 1.592e2, RT: 6.39 min
0.313 pg
S6 - PGE2/PGD2[1] (Unknown) 351.2 / 271.2 - 210715 Para LOD.wi...
Area: 1.699e3, Height: 3.176e2, RT: 6.40 min
0.625 pg
S5 - PGE2/PGD2[1] (Unknown) 351.2 / 271.2 - 210715 Para LOD.wi...
Area: 3.110e3, Height: 5.761e2, RT: 6.39 min
1.25 pg
S4 - PGE2/PGD2[1] (Unknown) 351.2 / 271.2 - 210715 Para LOD.wi...
Area: 6.121e3, Height: 9.686e2, RT: 6.39 min
2.50 pg
S3 - PGE2/PGD2[1] (Unknown) 351.2 / 271.2 - 210715 Para LOD.wi...
Area: 1.127e4, Height: 1.883e3, RT: 6.39 min
5.00 pg
S2 - PGE2/PGD2[1] (Unknown) 351.2 / 271.2 - 210715 Para LOD.wi...
Area: 2.558e4, Height: 3.138e3, RT: 6.40 min
10.0 pg
S1 - PGE2/PGD2[1] (Unknown) 351.2 / 271.2 - 210715 Para LOD.wi...
Area: 5.481e4, Height: 7.404e3, RT: 6.40 min
20.0 pg
Intensity
Time, min

PGD2

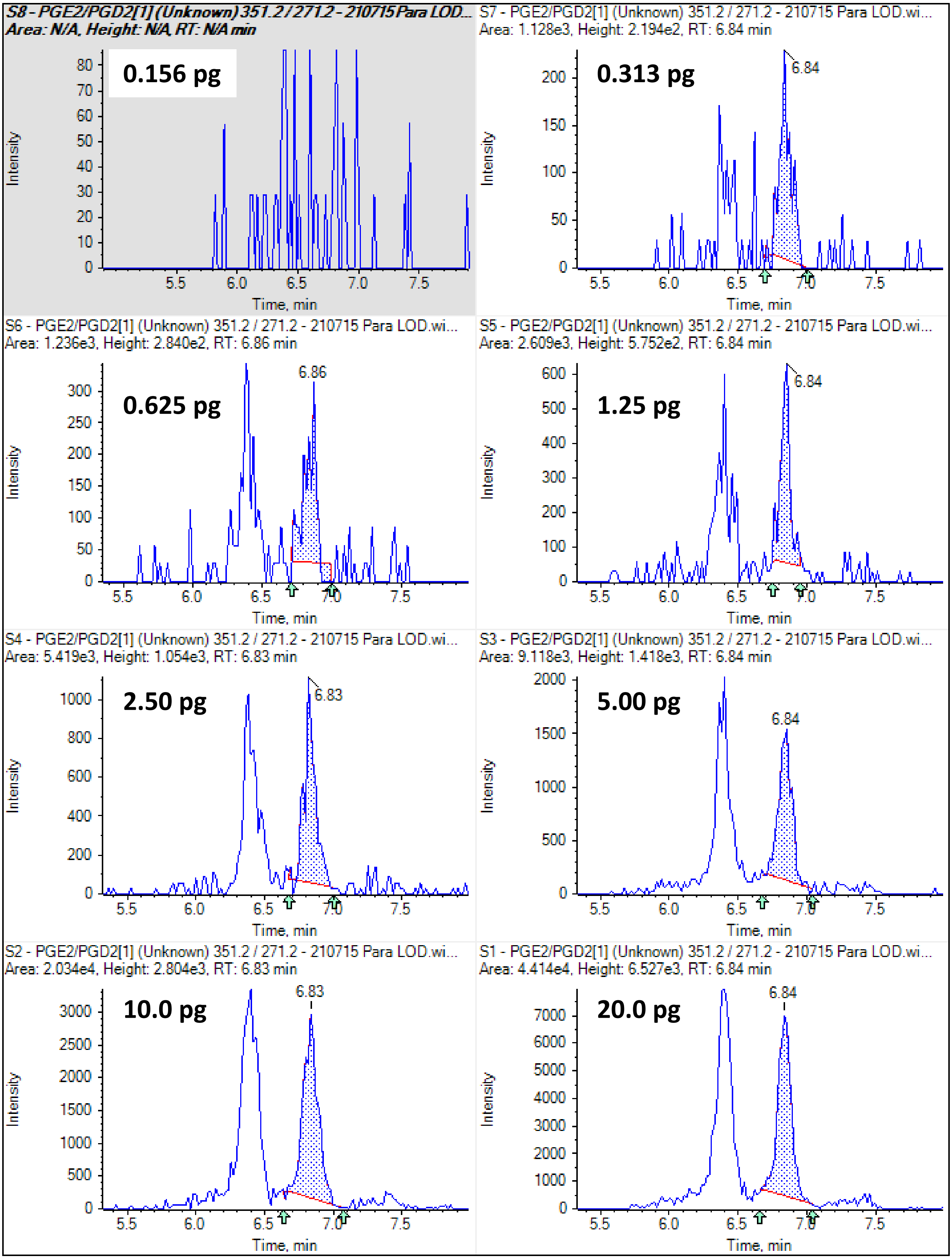

S8 - PGE2/PGD2[1] (Unknown) 351.2 / 271.2 - 210715 Para LOD...
Area: N/A, Height: N/A, RT: N/A min
0.156 pg
S7 - PGE2/PGD2[1] (Unknown) 351.2 / 271.2 - 210715 Para LOD.wi...
Area: 1.128e3, Height: 2.194e2, RT: 6.84 min
0.313 pg
6.84
S6 - PGE2/PGD2[1] (Unknown) 351.2 / 271.2 - 210715 Para LOD.wi...
Area: 1.236e3, Height: 2.840e2, RT: 6.86 min
0.625 pg
6.86
S5 - PGE2/PGD2[1] (Unknown) 351.2 / 271.2 - 210715 Para LOD.wi...
Area: 2.609e3, Height: 5.752e2, RT: 6.84 min
1.25 pg
6.84
S4 - PGE2/PGD2[1] (Unknown) 351.2 / 271.2 - 210715 Para LOD.wi...
Area: 5.419e3, Height: 1.054e3, RT: 6.83 min
2.50 pg
6.83
S3 - PGE2/PGD2[1] (Unknown) 351.2 / 271.2 - 210715 Para LOD.wi...
Area: 9.118e3, Height: 1.418e3, RT: 6.84 min
5.00 pg
6.84
S2 - PGE2/PGD2[1] (Unknown) 351.2 / 271.2 - 210715 Para LOD.wi...
Area: 2.034e4, Height: 2.804e3, RT: 6.83 min
10.0 pg
6.83
S1 - PGE2/PGD2[1] (Unknown) 351.2 / 271.2 - 210715 Para LOD.wi...
Area: 4.414e4, Height: 6.527e3, RT: 6.84 min
20.0 pg
6.84
Intensity
Time, min

LxB4

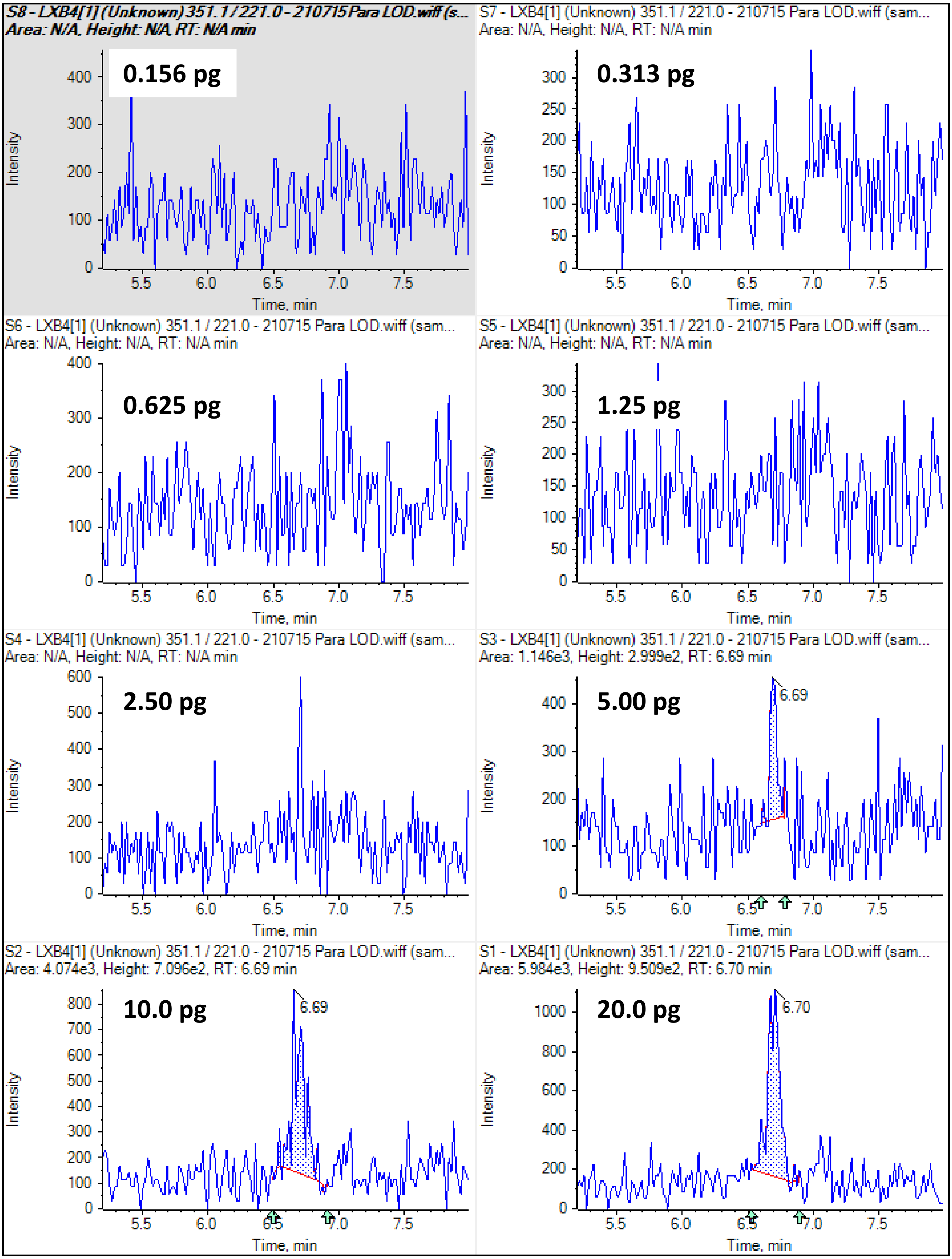

S8 - LXB4[1] (Unknown) 351.1 / 221.0 - 210715 Para LOD.wiff (s...
Area: N/A, Height: N/A, RT: N/A min
0.156 pg
S7 - LXB4[1] (Unknown) 351.1 / 221.0 - 210715 Para LOD.wiff (sam...
Area: N/A, Height: N/A, RT: N/A min
0.313 pg
S6 - LXB4[1] (Unknown) 351.1 / 221.0 - 210715 Para LOD.wiff (sam...
Area: N/A, Height: N/A, RT: N/A min
0.625 pg
S5 - LXB4[1] (Unknown) 351.1 / 221.0 - 210715 Para LOD.wiff (sam...
Area: N/A, Height: N/A, RT: N/A min
1.25 pg
S4 - LXB4[1] (Unknown) 351.1 / 221.0 - 210715 Para LOD.wiff (sam...
Area: N/A, Height: N/A, RT: N/A min
2.50 pg
S3 - LXB4[1] (Unknown) 351.1 / 221.0 - 210715 Para LOD.wiff (sam...
Area: 1.146e3, Height: 2.999e2, RT: 6.69 min
5.00 pg
6.69
S2 - LXB4[1] (Unknown) 351.1 / 221.0 - 210715 Para LOD.wiff (sam...
Area: 4.074e3, Height: 7.096e2, RT: 6.69 min
10.0 pg
6.69
S1 - LXB4[1] (Unknown) 351.1 / 221.0 - 210715 Para LOD.wiff (sam...
Area: 5.984e3, Height: 9.509e2, RT: 6.70 min
20.0 pg
6.70
Intensity
Time, min

LxA4

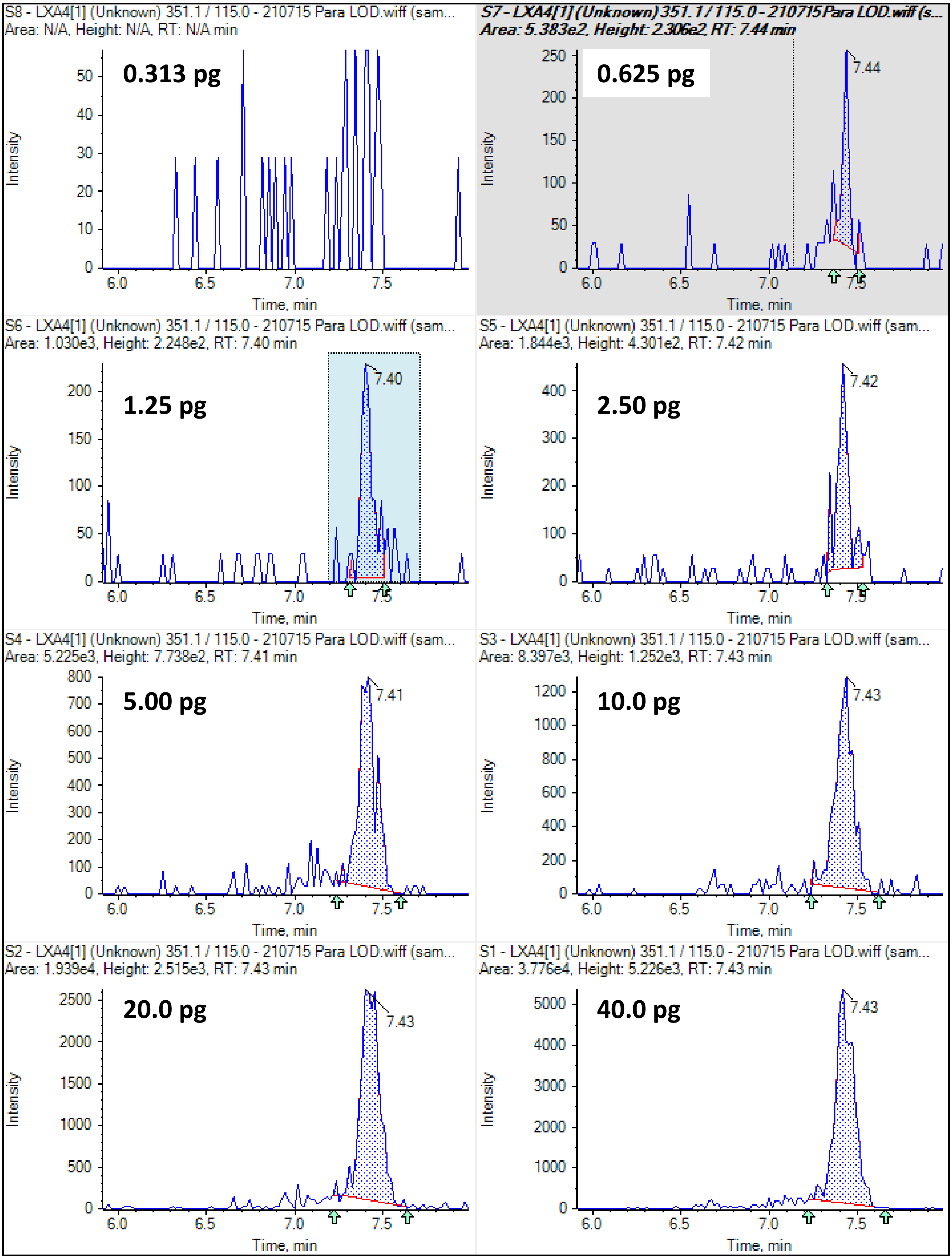
S8 - LXA4[1] (Unknown) 351.1 / 115.0 - 210715 Para LOD.wiff (sam...
Area: N/A, Height: N/A, RT: N/A min
0.313 pg
S7 - LXA4[1] (Unknown) 351.1 / 115.0 - 210715 Para LOD.wiff (s...
Area: 5.383e2, Height: 2.306e2, RT: 7.44 min
0.625 pg
7.44
S6 - LXA4[1] (Unknown) 351.1 / 115.0 - 210715 Para LOD.wiff (sam...
Area: 1.030e3, Height: 2.248e2, RT: 7.40 min
1.25 pg
7.40
S5 - LXA4[1] (Unknown) 351.1 / 115.0 - 210715 Para LOD.wiff (sam...
Area: 1.844e3, Height: 4.301e2, RT: 7.42 min
2.50 pg
7.42
S4 - LXA4[1] (Unknown) 351.1 / 115.0 - 210715 Para LOD.wiff (sam...
Area: 5.225e3, Height: 7.738e2, RT: 7.41 min
5.00 pg
7.41
S3 - LXA4[1] (Unknown) 351.1 / 115.0 - 210715 Para LOD.wiff (sam...
Area: 8.397e3, Height: 1.252e3, RT: 7.43 min
10.0 pg
7.43
S2 - LXA4[1] (Unknown) 351.1 / 115.0 - 210715 Para LOD.wiff (sam...
Area: 1.939e4, Height: 2.515e3, RT: 7.43 min
20.0 pg
7.43
S1 - LXA4[1] (Unknown) 351.1 / 115.0 - 210715 Para LOD.wiff (sam...
Area: 3.776e4, Height: 5.226e3, RT: 7.43 min
40.0 pg
7.43
Intensity
Time, min

RvD3

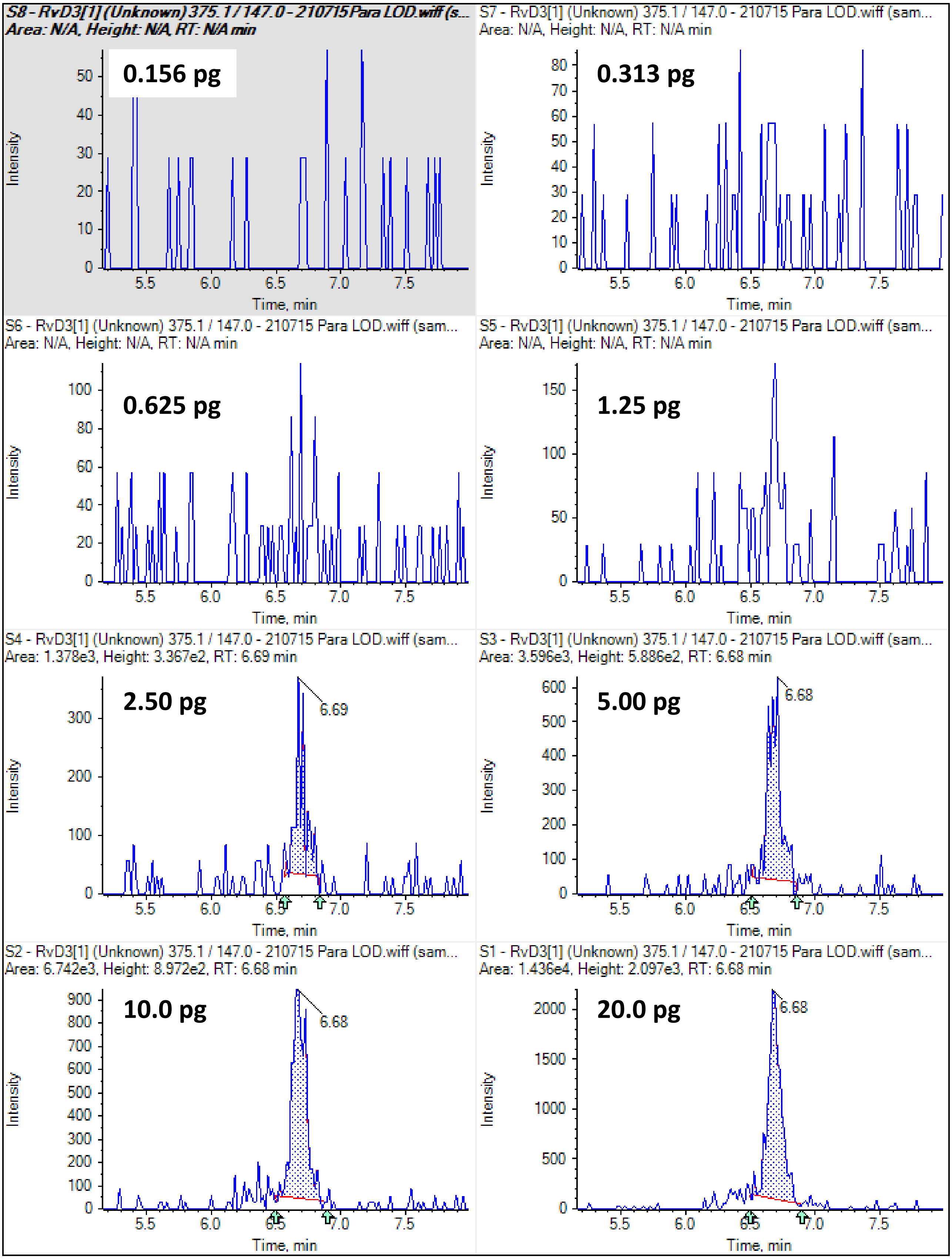
S8 - RvD3[1] (Unknown) 375.1 / 147.0 - 210715 Para LOD.wiff (s...
Area: N/A, Height: N/A, RT: N/A min
0.156 pg
S7 - RvD3[1] (Unknown) 375.1 / 147.0 - 210715 Para LOD.wiff (sam...
Area: N/A, Height: N/A, RT: N/A min
0.313 pg
S6 - RvD3[1] (Unknown) 375.1 / 147.0 - 210715 Para LOD.wiff (sam...
Area: N/A, Height: N/A, RT: N/A min
0.625 pg
S5 - RvD3[1] (Unknown) 375.1 / 147.0 - 210715 Para LOD.wiff (sam...
Area: N/A, Height: N/A, RT: N/A min
1.25 pg
S4 - RvD3[1] (Unknown) 375.1 / 147.0 - 210715 Para LOD.wiff (sam...
Area: 1.378e3, Height: 3.367e2, RT: 6.69 min
2.50 pg
6.69
S3 - RvD3[1] (Unknown) 375.1 / 147.0 - 210715 Para LOD.wiff (sam...
Area: 3.596e3, Height: 5.886e2, RT: 6.68 min
5.00 pg
6.68
S2 - RvD3[1] (Unknown) 375.1 / 147.0 - 210715 Para LOD.wiff (sam...
Area: 6.742e3, Height: 8.972e2, RT: 6.68 min
10.0 pg
6.68
S1 - RvD3[1] (Unknown) 375.1 / 147.0 - 210715 Para LOD.wiff (sam...
Area: 1.436e4, Height: 2.097e3, RT: 6.68 min
20.0 pg
6.68
Intensity
Time, min

RvD2

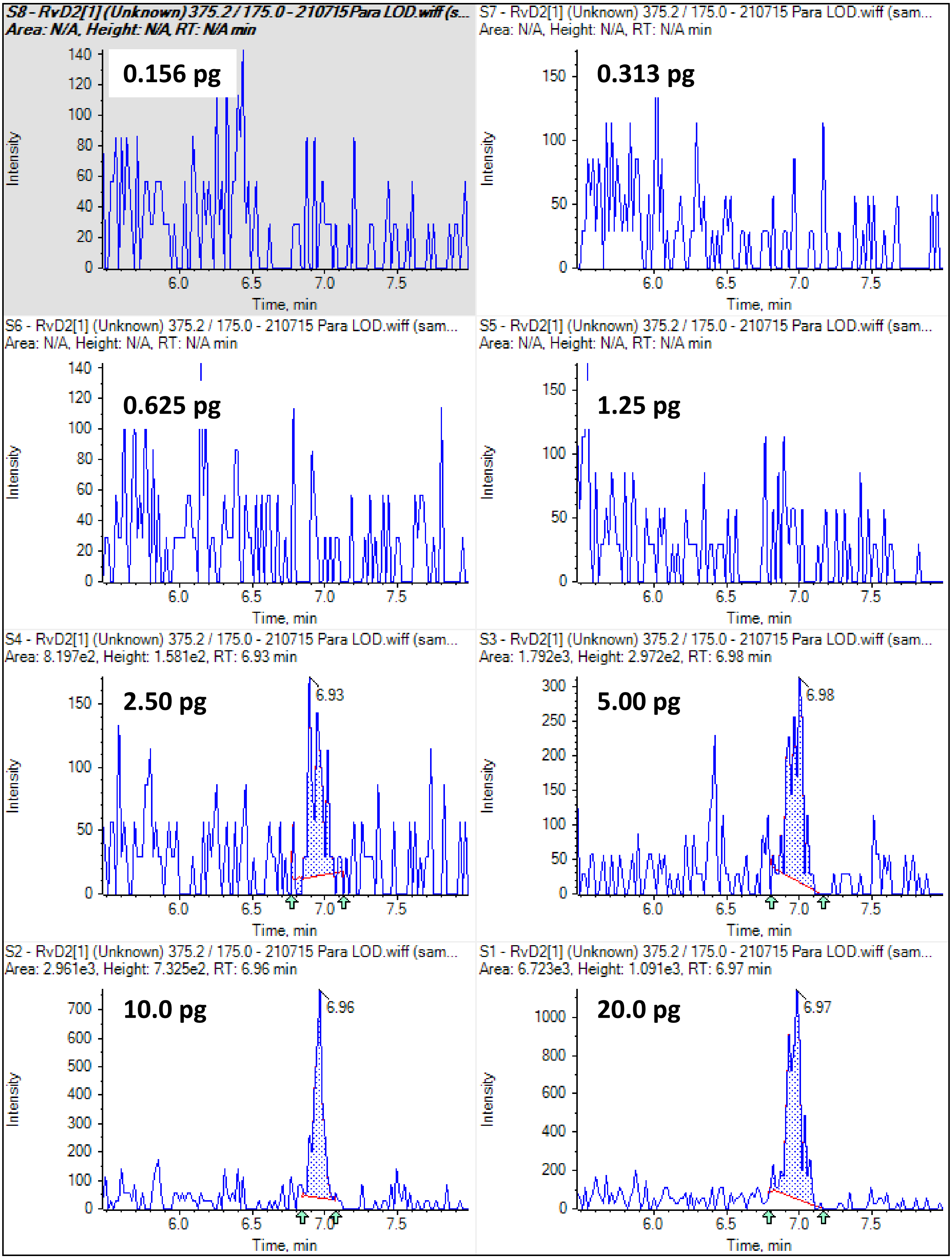
S8 - RvD2[1] (Unknown) 375.2 / 175.0 - 210715 Para LOD.wiff (s...
Area: N/A, Height: N/A, RT: N/A min
0.156 pg
S7 - RvD2[1] (Unknown) 375.2 / 175.0 - 210715 Para LOD.wiff (sam...
Area: N/A, Height: N/A, RT: N/A min
0.313 pg
S6 - RvD2[1] (Unknown) 375.2 / 175.0 - 210715 Para LOD.wiff (sam...
Area: N/A, Height: N/A, RT: N/A min
0.625 pg
S5 - RvD2[1] (Unknown) 375.2 / 175.0 - 210715 Para LOD.wiff (sam...
Area: N/A, Height: N/A, RT: N/A min
1.25 pg
S4 - RvD2[1] (Unknown) 375.2 / 175.0 - 210715 Para LOD.wiff (sam...
Area: 8.197e2, Height: 1.581e2, RT: 6.93 min
2.50 pg
6.93
S3 - RvD2[1] (Unknown) 375.2 / 175.0 - 210715 Para LOD.wiff (sam...
Area: 1.792e3, Height: 2.972e2, RT: 6.98 min
5.00 pg
6.98
S2 - RvD2[1] (Unknown) 375.2 / 175.0 - 210715 Para LOD.wiff (sam...
Area: 2.961e3, Height: 7.325e2, RT: 6.96 min
10.0 pg
6.96
S1 - RvD2[1] (Unknown) 375.2 / 175.0 - 210715 Para LOD.wiff (sam...
Area: 6.723e3, Height: 1.091e3, RT: 6.97 min
20.0 pg
6.97
Intensity
Time, min
6.0
6.5
7.0
7.5

RvD1- no válido

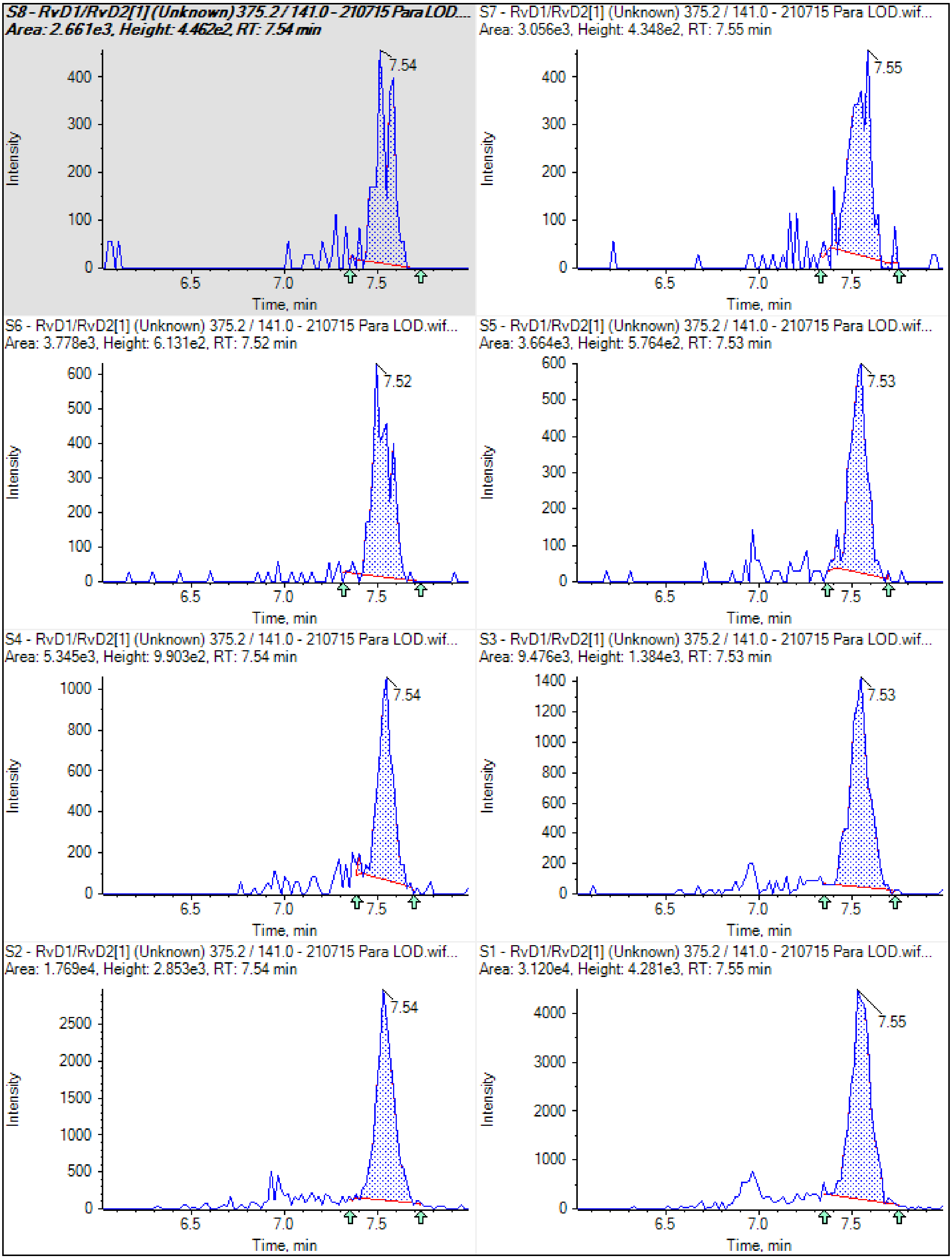

RvD5

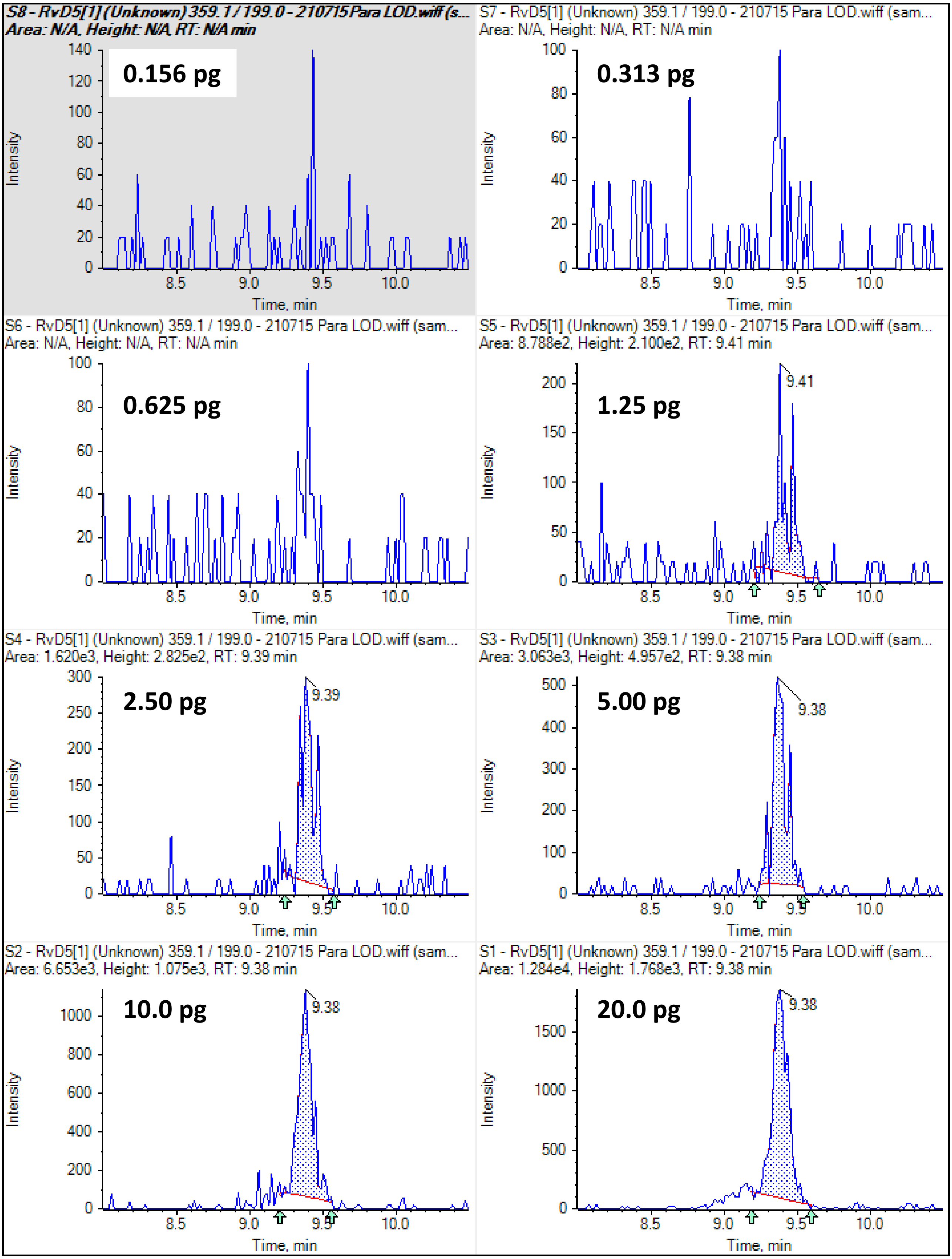
S8 - RvD5[1] (Unknown) 359.1 / 199.0 - 210715 Para LOD.wiff (s...
Area: N/A, Height: N/A, RT: N/A min
0.156 pg
S7 - RvD5[1] (Unknown) 359.1 / 199.0 - 210715 Para LOD.wiff (sam...
Area: N/A, Height: N/A, RT: N/A min
0.313 pg
S6 - RvD5[1] (Unknown) 359.1 / 199.0 - 210715 Para LOD.wiff (sam...
Area: N/A, Height: N/A, RT: N/A min
0.625 pg
S5 - RvD5[1] (Unknown) 359.1 / 199.0 - 210715 Para LOD.wiff (sam...
Area: 8.788e2, Height: 2.100e2, RT: 9.41 min
1.25 pg
9.41
S4 - RvD5[1] (Unknown) 359.1 / 199.0 - 210715 Para LOD.wiff (sam...
Area: 1.620e3, Height: 2.825e2, RT: 9.39 min
2.50 pg
9.39
S3 - RvD5[1] (Unknown) 359.1 / 199.0 - 210715 Para LOD.wiff (sam...
Area: 3.063e3, Height: 4.957e2, RT: 9.38 min
5.00 pg
9.38
S2 - RvD5[1] (Unknown) 359.1 / 199.0 - 210715 Para LOD.wiff (sam...
Area: 6.653e3, Height: 1.075e3, RT: 9.38 min
10.0 pg
9.38
S1 - RvD5[1] (Unknown) 359.1 / 199.0 - 210715 Para LOD.wiff (sam...
Area: 1.284e4, Height: 1.768e3, RT: 9.38 min
20.0 pg
9.38
Intensity
Time, min
8.5
9.0
9.5
10.0

MaR1

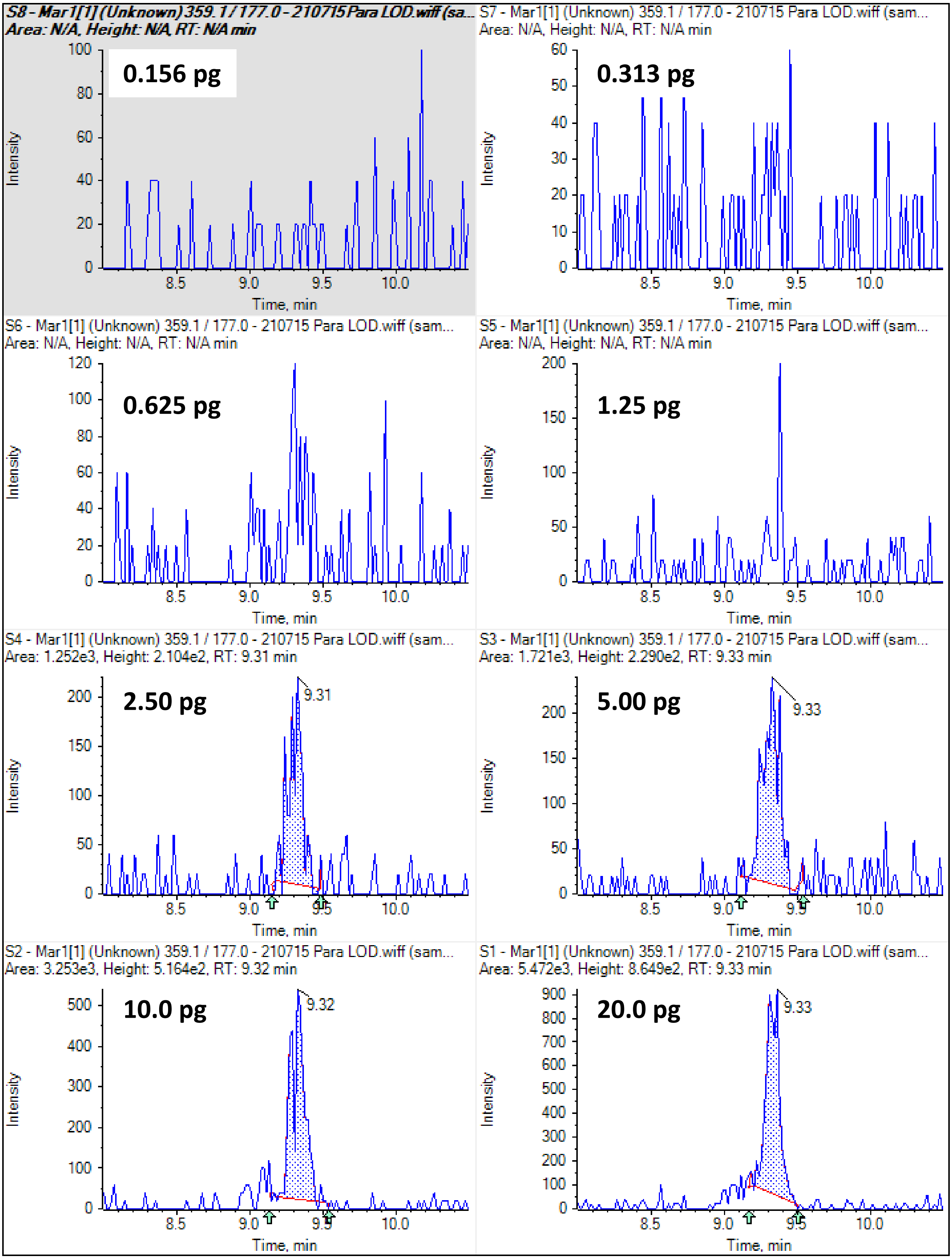

S8 - Mar1[1] (Unknown) 359.1 / 177.0 - 210715 Para LOD.wiff (sa...
Area: N/A, Height: N/A, RT: N/A min
0.156 pg
S7 - Mar1[1] (Unknown) 359.1 / 177.0 - 210715 Para LOD.wiff (sam...
Area: N/A, Height: N/A, RT: N/A min
0.313 pg
S6 - Mar1[1] (Unknown) 359.1 / 177.0 - 210715 Para LOD.wiff (sam...
Area: N/A, Height: N/A, RT: N/A min
0.625 pg
S5 - Mar1[1] (Unknown) 359.1 / 177.0 - 210715 Para LOD.wiff (sam...
Area: N/A, Height: N/A, RT: N/A min
1.25 pg
S4 - Mar1[1] (Unknown) 359.1 / 177.0 - 210715 Para LOD.wiff (sam...
Area: 1.252e3, Height: 2.104e2, RT: 9.31 min
2.50 pg
9.31
S3 - Mar1[1] (Unknown) 359.1 / 177.0 - 210715 Para LOD.wiff (sam...
Area: 1.721e3, Height: 2.290e2, RT: 9.33 min
5.00 pg
9.33
S2 - Mar1[1] (Unknown) 359.1 / 177.0 - 210715 Para LOD.wiff (sam...
Area: 3.253e3, Height: 5.164e2, RT: 9.32 min
10.0 pg
9.32
S1 - Mar1[1] (Unknown) 359.1 / 177.0 - 210715 Para LOD.wiff (sam...
Area: 5.472e3, Height: 8.649e2, RT: 9.33 min
20.0 pg
9.33
Intensity
Time, min

PD1

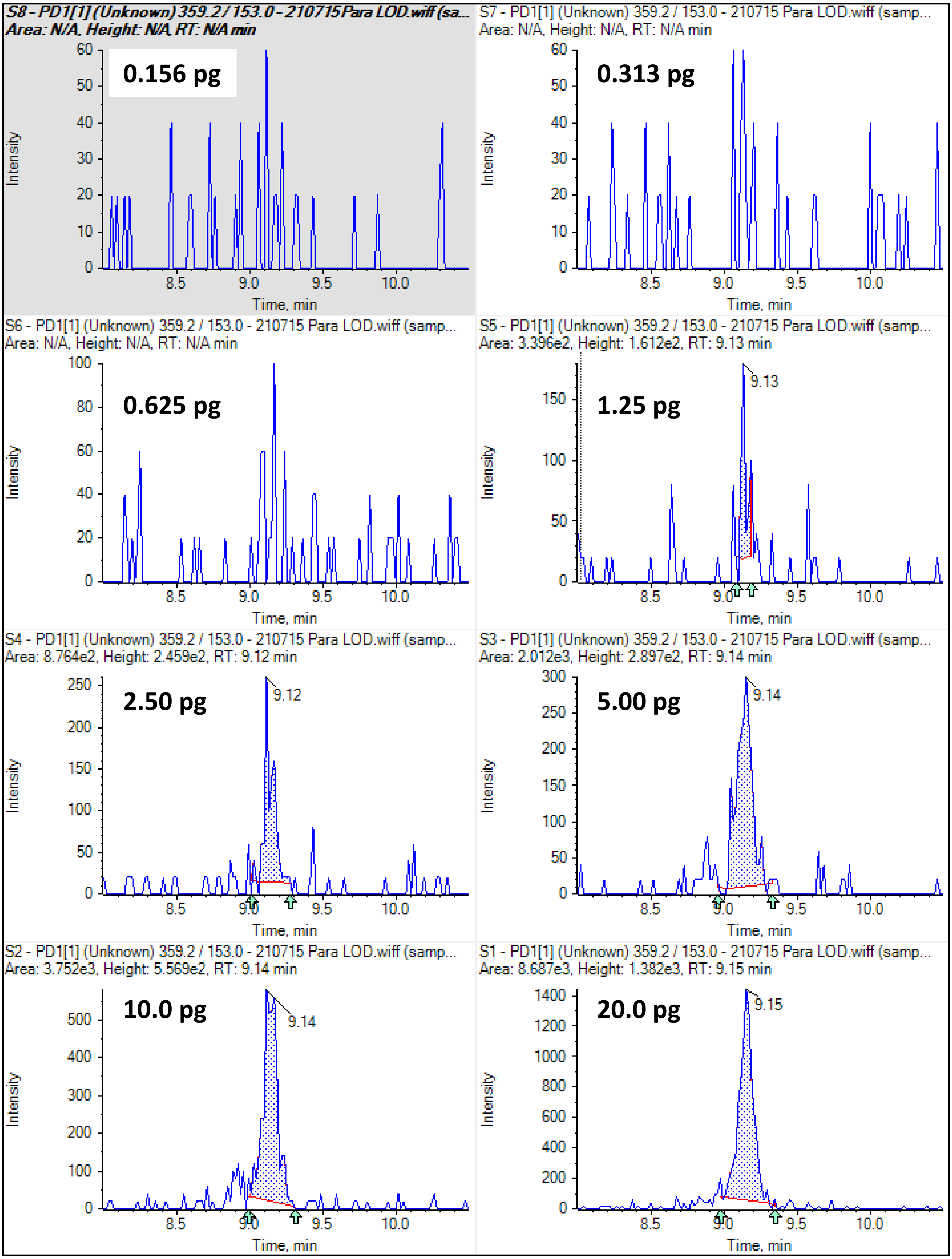

S8 - PD1[1] (Unknown) 359.2 / 153.0 - 210715 Para LOD.wiff (sa...
Area: N/A, Height: N/A, RT: N/A min
0.156 pg
S7 - PD1[1] (Unknown) 359.2 / 153.0 - 210715 Para LOD.wiff (samp...
Area: N/A, Height: N/A, RT: N/A min
0.313 pg
S6 - PD1[1] (Unknown) 359.2 / 153.0 - 210715 Para LOD.wiff (samp...
Area: N/A, Height: N/A, RT: N/A min
0.625 pg
S5 - PD1[1] (Unknown) 359.2 / 153.0 - 210715 Para LOD.wiff (samp...
Area: 3.396e2, Height: 1.612e2, RT: 9.13 min
1.25 pg
9.13
S4 - PD1[1] (Unknown) 359.2 / 153.0 - 210715 Para LOD.wiff (samp...
Area: 8.764e2, Height: 2.459e2, RT: 9.12 min
2.50 pg
9.12
S3 - PD1[1] (Unknown) 359.2 / 153.0 - 210715 Para LOD.wiff (samp...
Area: 2.012e3, Height: 2.897e2, RT: 9.14 min
5.00 pg
9.14
S2 - PD1[1] (Unknown) 359.2 / 153.0 - 210715 Para LOD.wiff (samp...
Area: 3.752e3, Height: 5.569e2, RT: 9.14 min
10.0 pg
9.14
S1 - PD1[1] (Unknown) 359.2 / 153.0 - 210715 Para LOD.wiff (samp...
Area: 8.687e3, Height: 1.382e3, RT: 9.15 min
20.0 pg
9.15
Intensity
Time, min
8.5
9.0
9.5
10.0

LTB4

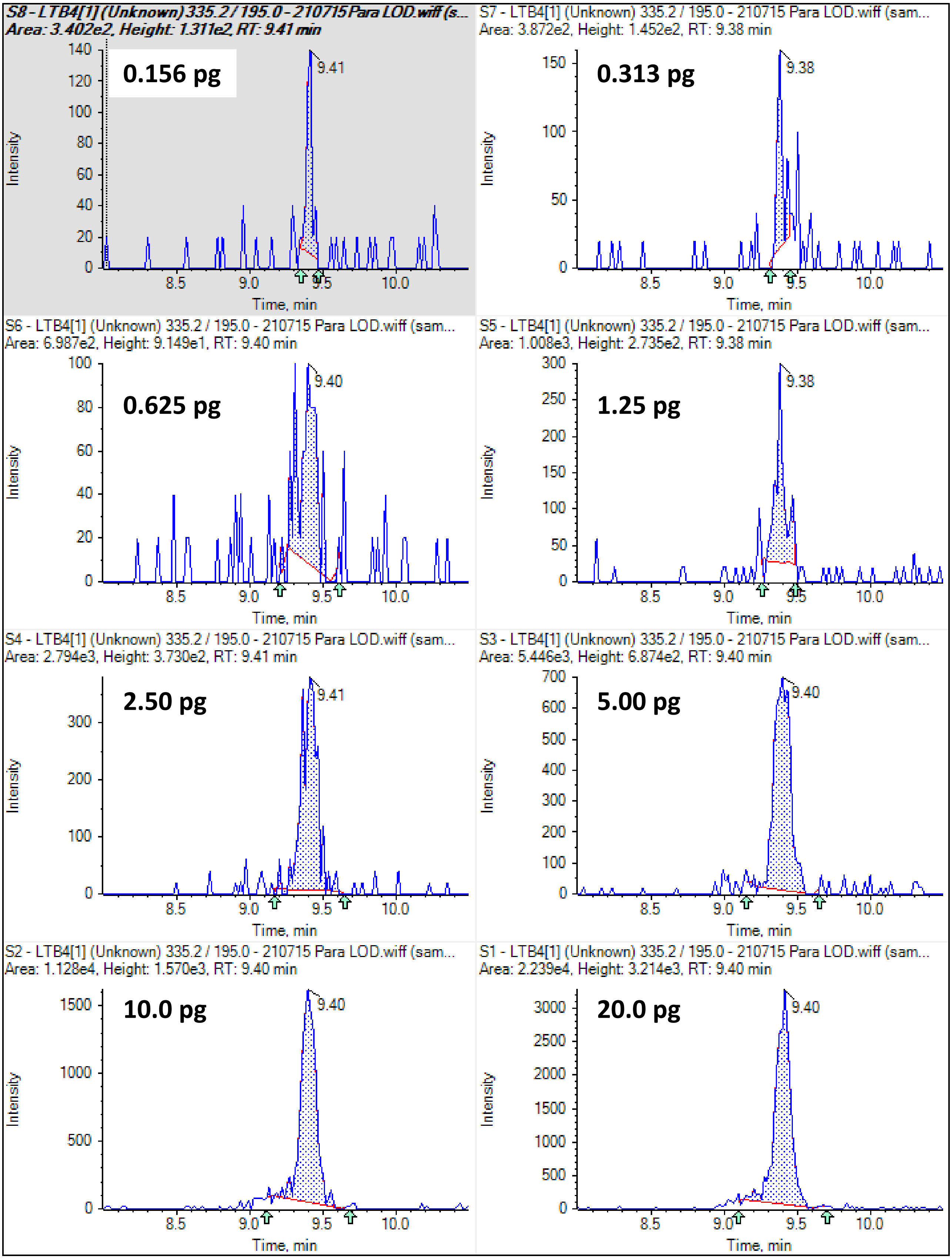
S8 - LTB4[1] (Unknown) 335.2 / 195.0 - 210715 Para LOD.wiff (s...
Area: 3.402e2, Height: 1.311e2, RT: 9.41 min
0.156 pg
9.41
S7 - LTB4[1] (Unknown) 335.2 / 195.0 - 210715 Para LOD.wiff (sam...
Area: 3.872e2, Height: 1.452e2, RT: 9.38 min
0.313 pg
9.38
S6 - LTB4[1] (Unknown) 335.2 / 195.0 - 210715 Para LOD.wiff (sam...
Area: 6.987e2, Height: 9.149e1, RT: 9.40 min
0.625 pg
9.40
S5 - LTB4[1] (Unknown) 335.2 / 195.0 - 210715 Para LOD.wiff (sam...
Area: 1.008e3, Height: 2.735e2, RT: 9.38 min
1.25 pg
9.38
S4 - LTB4[1] (Unknown) 335.2 / 195.0 - 210715 Para LOD.wiff (sam...
Area: 2.794e3, Height: 3.730e2, RT: 9.41 min
2.50 pg
9.41
S3 - LTB4[1] (Unknown) 335.2 / 195.0 - 210715 Para LOD.wiff (sam...
Area: 5.446e3, Height: 6.874e2, RT: 9.40 min
5.00 pg
9.40
S2 - LTB4[1] (Unknown) 335.2 / 195.0 - 210715 Para LOD.wiff (sam...
Area: 1.128e4, Height: 1.570e3, RT: 9.40 min
10.0 pg
9.40
S1 - LTB4[1] (Unknown) 335.2 / 195.0 - 210715 Para LOD.wiff (sam...
Area: 2.239e4, Height: 3.214e3, RT: 9.40 min
20.0 pg
9.40
Intensity
Time, min
8.5
9.0
9.5
10.0

12,13-diHOME

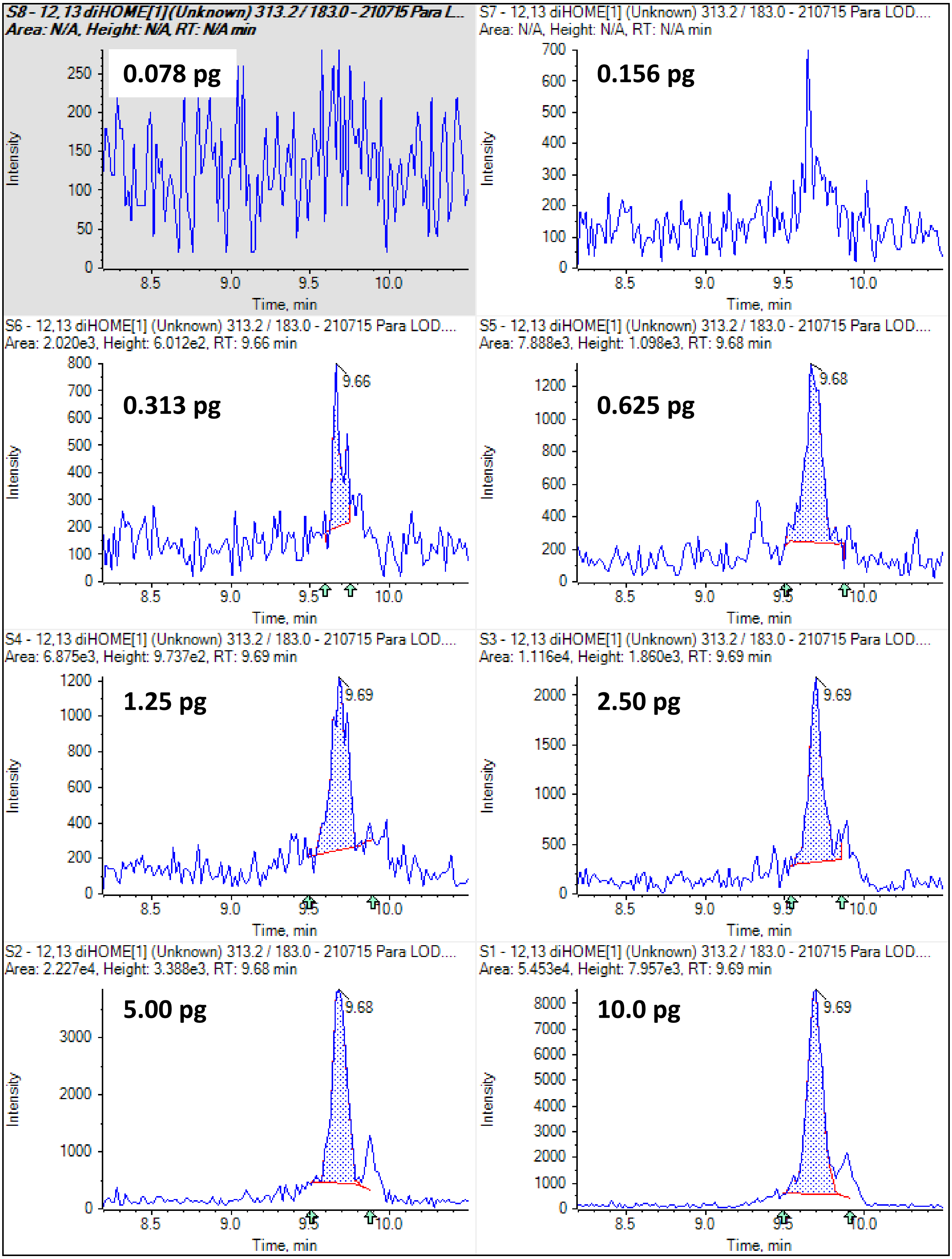
S8 - 12,13 diHOME[1] (Unknown) 313.2 / 183.0 - 210715 Para L...
Area: N/A, Height: N/A, RT: N/A min
0.078 pg
S7 - 12,13 diHOME[1] (Unknown) 313.2 / 183.0 - 210715 Para LOD....
Area: N/A, Height: N/A, RT: N/A min
0.156 pg
S6 - 12,13 diHOME[1] (Unknown) 313.2 / 183.0 - 210715 Para LOD....
Area: 2.020e3, Height: 6.012e2, RT: 9.66 min
0.313 pg
S5 - 12,13 diHOME[1] (Unknown) 313.2 / 183.0 - 210715 Para LOD....
Area: 7.888e3, Height: 1.098e3, RT: 9.68 min
0.625 pg
S4 - 12,13 diHOME[1] (Unknown) 313.2 / 183.0 - 210715 Para LOD....
Area: 6.875e3, Height: 9.737e2, RT: 9.69 min
1.25 pg
S3 - 12,13 diHOME[1] (Unknown) 313.2 / 183.0 - 210715 Para LOD....
Area: 1.116e4, Height: 1.860e3, RT: 9.69 min
2.50 pg
S2 - 12,13 diHOME[1] (Unknown) 313.2 / 183.0 - 210715 Para LOD....
Area: 2.227e4, Height: 3.388e3, RT: 9.68 min
5.00 pg
S1 - 12,13 diHOME[1] (Unknown) 313.2 / 183.0 - 210715 Para LOD....
Area: 5.453e4, Height: 7.957e3, RT: 9.69 min
10.0 pg
Intensity
Time, min

9,10-diHOME

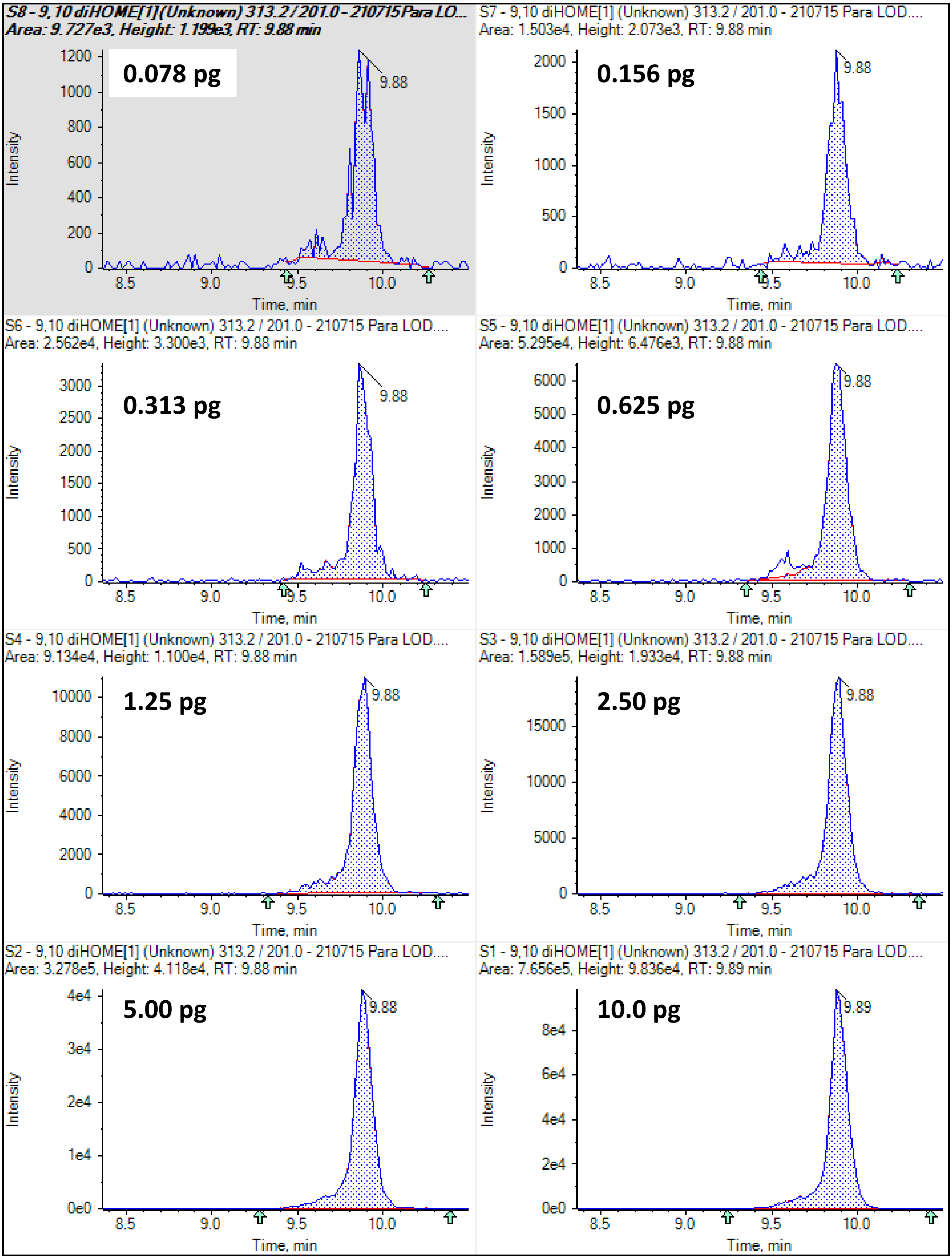
S8 - 9,10 diHOME[1] (Unknown) 313.2 / 201.0 - 210715 Para LO...
Area: 9.727e3, Height: 1.199e3, RT: 9.88 min
0.078 pg
S7 - 9,10 diHOME[1] (Unknown) 313.2 / 201.0 - 210715 Para LOD....
Area: 1.503e4, Height: 2.073e3, RT: 9.88 min
0.156 pg
S6 - 9,10 diHOME[1] (Unknown) 313.2 / 201.0 - 210715 Para LOD....
Area: 2.562e4, Height: 3.300e3, RT: 9.88 min
0.313 pg
S5 - 9,10 diHOME[1] (Unknown) 313.2 / 201.0 - 210715 Para LOD....
Area: 5.295e4, Height: 6.476e3, RT: 9.88 min
0.625 pg
S4 - 9,10 diHOME[1] (Unknown) 313.2 / 201.0 - 210715 Para LOD....
Area: 9.134e4, Height: 1.100e4, RT: 9.88 min
1.25 pg
S3 - 9,10 diHOME[1] (Unknown) 313.2 / 201.0 - 210715 Para LOD....
Area: 1.589e5, Height: 1.933e4, RT: 9.88 min
2.50 pg
S2 - 9,10 diHOME[1] (Unknown) 313.2 / 201.0 - 210715 Para LOD....
Area: 3.278e5, Height: 4.118e4, RT: 9.88 min
5.00 pg
S1 - 9,10 diHOME[1] (Unknown) 313.2 / 201.0 - 210715 Para LOD....
Area: 7.656e5, Height: 9.836e4, RT: 9.89 min
10.0 pg
Intensity
Time, min

18-HEPE

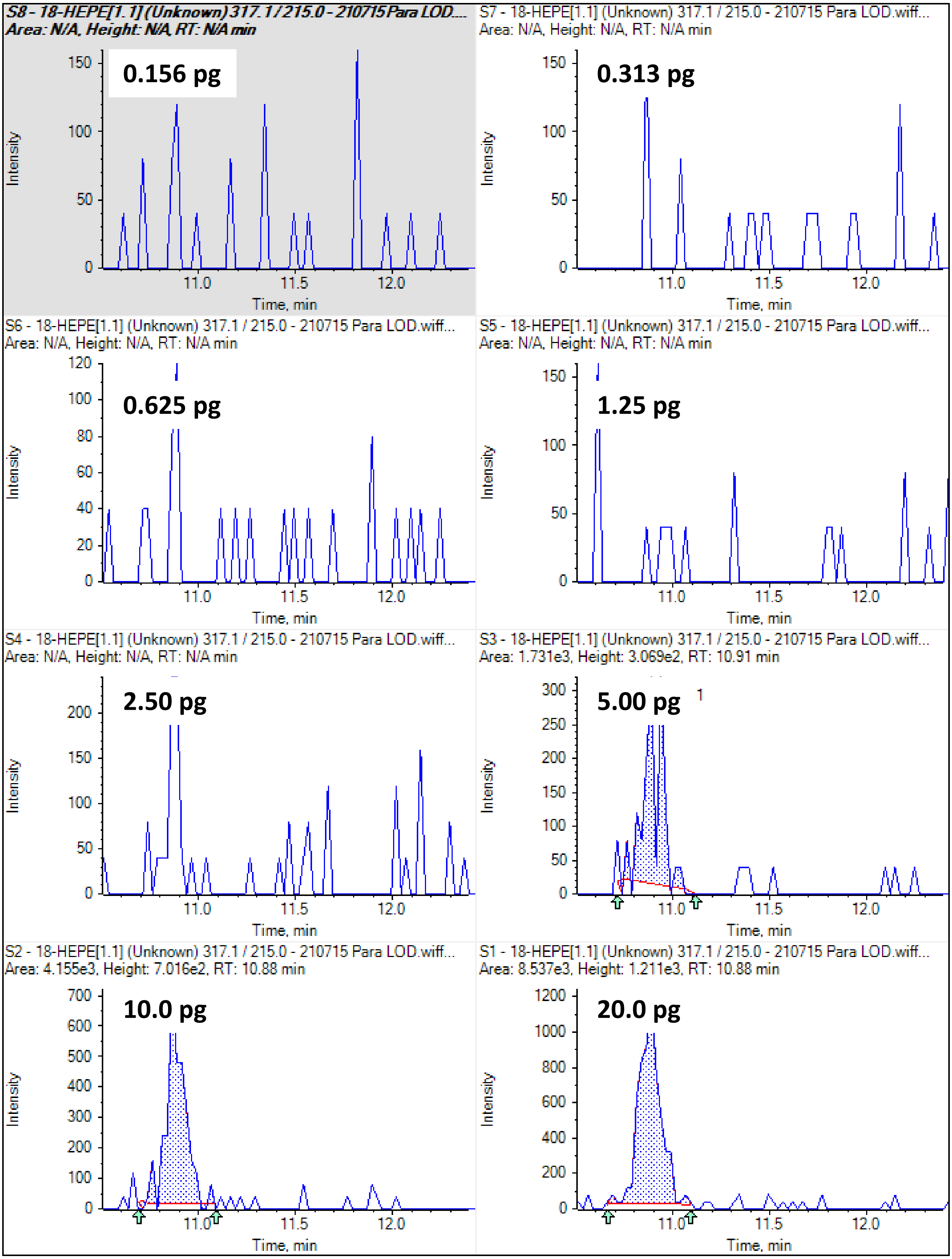

S8 - 18-HEPE[1.1] (Unknown) 317.1 / 215.0 - 210715 Para LOD....
Area: N/A, Height: N/A, RT: N/A min
0.156 pg
S7 - 18-HEPE[1.1] (Unknown) 317.1 / 215.0 - 210715 Para LOD.wiff...
Area: N/A, Height: N/A, RT: N/A min
0.313 pg
S6 - 18-HEPE[1.1] (Unknown) 317.1 / 215.0 - 210715 Para LOD.wiff...
Area: N/A, Height: N/A, RT: N/A min
0.625 pg
S5 - 18-HEPE[1.1] (Unknown) 317.1 / 215.0 - 210715 Para LOD.wiff...
Area: N/A, Height: N/A, RT: N/A min
1.25 pg
S4 - 18-HEPE[1.1] (Unknown) 317.1 / 215.0 - 210715 Para LOD.wiff...
Area: N/A, Height: N/A, RT: N/A min
2.50 pg
S3 - 18-HEPE[1.1] (Unknown) 317.1 / 215.0 - 210715 Para LOD.wiff...
Area: 1.731e3, Height: 3.069e2, RT: 10.91 min
5.00 pg
S2 - 18-HEPE[1.1] (Unknown) 317.1 / 215.0 - 210715 Para LOD.wiff...
Area: 4.155e3, Height: 7.016e2, RT: 10.88 min
10.0 pg
S1 - 18-HEPE[1.1] (Unknown) 317.1 / 215.0 - 210715 Para LOD.wiff...
Area: 8.537e3, Height: 1.211e3, RT: 10.88 min
20.0 pg
Intensity
Time, min

13-HODE

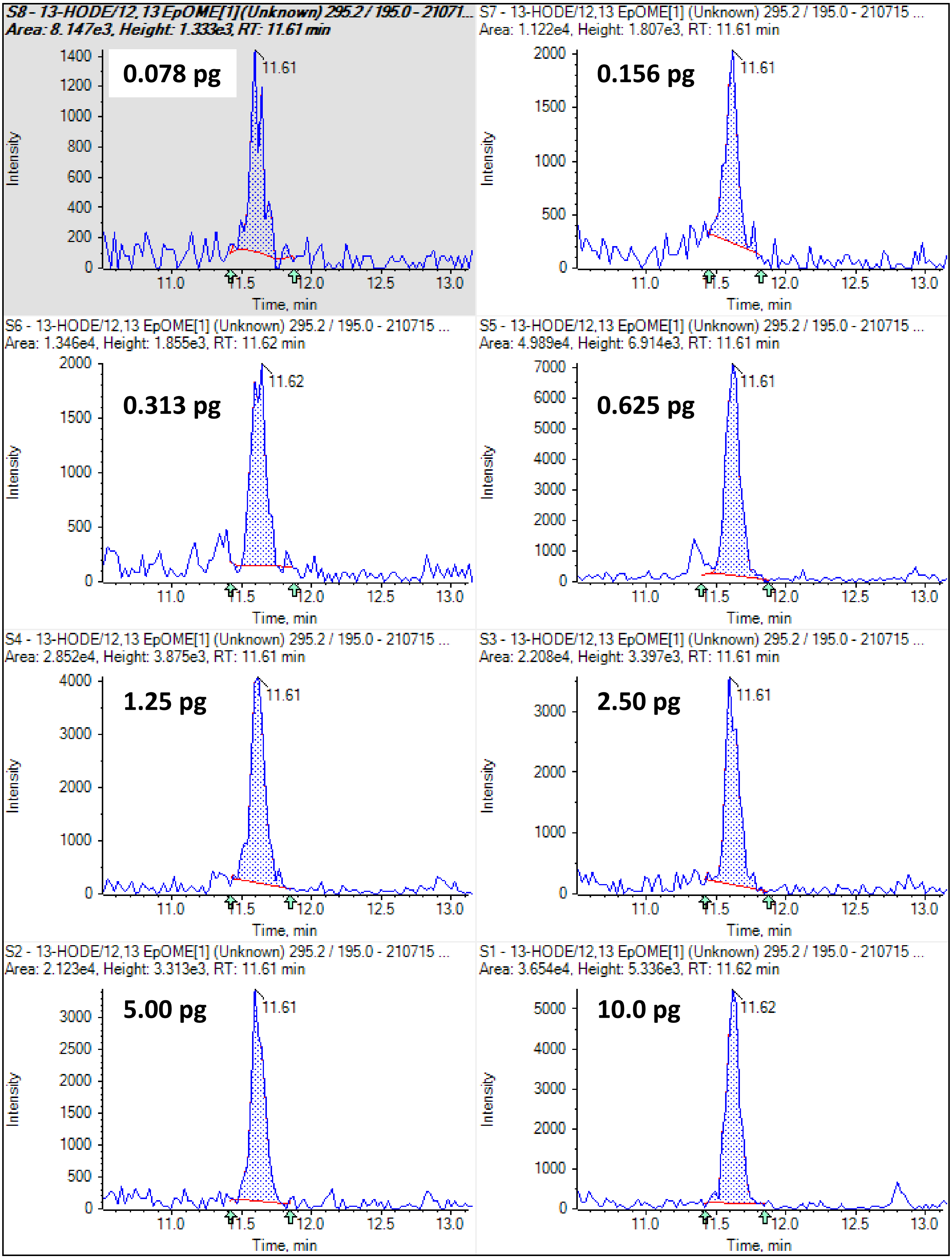
S8 - 13-HODE/12,13 EpOME[1](Unknown) 295.2 / 195.0 - 21071...
Area: 8.147e3, Height: 1.333e3, RT: 11.61 min
0.078 pg
S7 - 13-HODE/12,13 EpOME[1] (Unknown) 295.2 / 195.0 - 210715 ...
Area: 1.122e4, Height: 1.807e3, RT: 11.61 min
0.156 pg
S6 - 13-HODE/12,13 EpOME[1] (Unknown) 295.2 / 195.0 - 210715 ...
Area: 1.346e4, Height: 1.855e3, RT: 11.62 min
0.313 pg
S5 - 13-HODE/12,13 EpOME[1] (Unknown) 295.2 / 195.0 - 210715 ...
Area: 4.989e4, Height: 6.914e3, RT: 11.61 min
0.625 pg
S4 - 13-HODE/12,13 EpOME[1] (Unknown) 295.2 / 195.0 - 210715 ...
Area: 2.852e4, Height: 3.875e3, RT: 11.61 min
1.25 pg
S3 - 13-HODE/12,13 EpOME[1] (Unknown) 295.2 / 195.0 - 210715 ...
Area: 2.208e4, Height: 3.397e3, RT: 11.61 min
2.50 pg
S2 - 13-HODE/12,13 EpOME[1] (Unknown) 295.2 / 195.0 - 210715 ...
Area: 2.123e4, Height: 3.313e3, RT: 11.61 min
5.00 pg
S1 - 13-HODE/12,13 EpOME[1] (Unknown) 295.2 / 195.0 - 210715 ...
Area: 3.654e4, Height: 5.336e3, RT: 11.62 min
10.0 pg
Intensity
Time, min

9-HODE

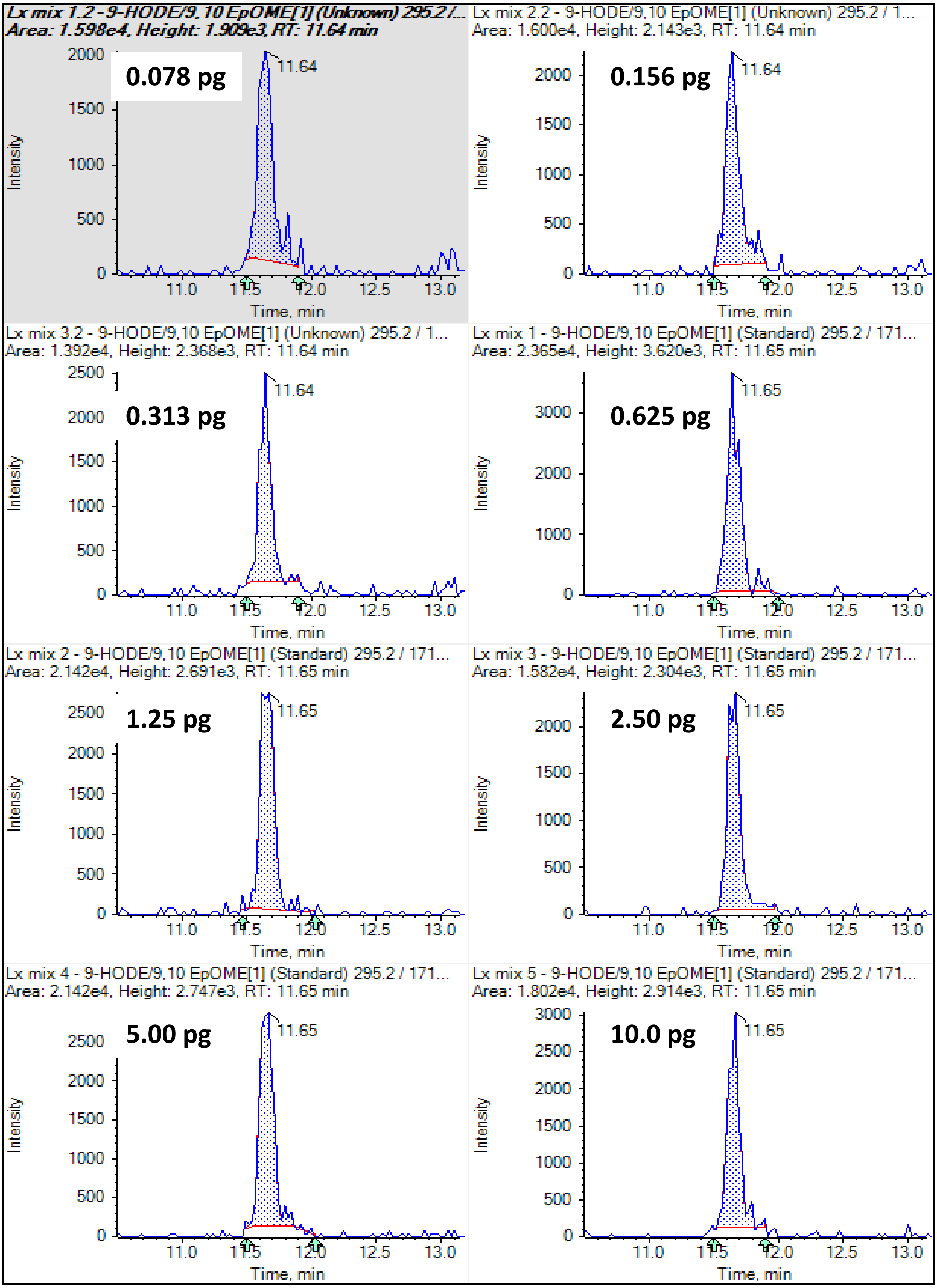

Lx mix 1.2 - 9-HODE/9,10 EpOME[1] (Unknown) 295.2 /...
Area: 1.598e4, Height: 1.909e3, RT: 11.64 min
0.078 pg
Lx mix 2.2 - 9-HODE/9,10 EpOME[1] (Unknown) 295.2 / 1...
Area: 1.600e4, Height: 2.143e3, RT: 11.64 min
0.156 pg
Lx mix 3.2 - 9-HODE/9,10 EpOME[1] (Unknown) 295.2 / 1...
Area: 1.392e4, Height: 2.368e3, RT: 11.64 min
0.313 pg
Lx mix 1 - 9-HODE/9,10 EpOME[1] (Standard) 295.2 / 171...
Area: 2.365e4, Height: 3.620e3, RT: 11.65 min
0.625 pg
Lx mix 2 - 9-HODE/9,10 EpOME[1] (Standard) 295.2 / 171...
Area: 2.142e4, Height: 2.691e3, RT: 11.65 min
1.25 pg
Lx mix 3 - 9-HODE/9,10 EpOME[1] (Standard) 295.2 / 171...
Area: 1.582e4, Height: 2.304e3, RT: 11.65 min
2.50 pg
Lx mix 4 - 9-HODE/9,10 EpOME[1] (Standard) 295.2 / 171...
Area: 2.142e4, Height: 2.747e3, RT: 11.65 min
5.00 pg
Lx mix 5 - 9-HODE/9,10 EpOME[1] (Standard) 295.2 / 171...
Area: 1.802e4, Height: 2.914e3, RT: 11.65 min
10.0 pg
Intensity
Time, min

15-HETE

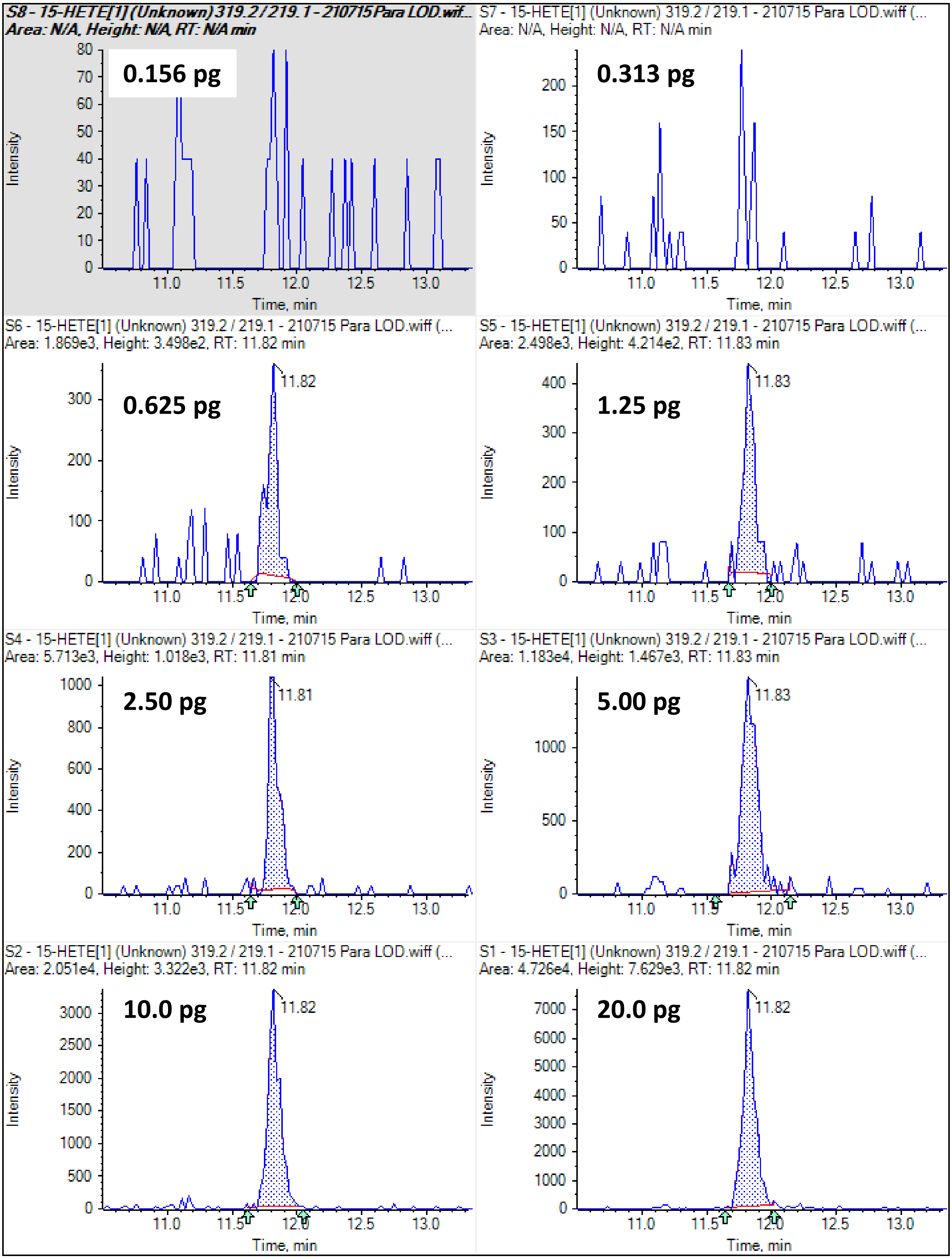

S8 - 15-HETE[1] (Unknown) 319.2 / 219.1 - 210715 Para LOD.wif...
Area: N/A, Height: N/A, RT: N/A min
0.156 pg
S7 - 15-HETE[1] (Unknown) 319.2 / 219.1 - 210715 Para LOD.wiff (...
Area: N/A, Height: N/A, RT: N/A min
0.313 pg
S6 - 15-HETE[1] (Unknown) 319.2 / 219.1 - 210715 Para LOD.wiff (...
Area: 1.869e3, Height: 3.498e2, RT: 11.82 min
0.625 pg
11.82
S5 - 15-HETE[1] (Unknown) 319.2 / 219.1 - 210715 Para LOD.wiff (...
Area: 2.498e3, Height: 4.214e2, RT: 11.83 min
1.25 pg
11.83
S4 - 15-HETE[1] (Unknown) 319.2 / 219.1 - 210715 Para LOD.wiff (...
Area: 5.713e3, Height: 1.018e3, RT: 11.81 min
2.50 pg
11.81
S3 - 15-HETE[1] (Unknown) 319.2 / 219.1 - 210715 Para LOD.wiff (...
Area: 1.183e4, Height: 1.467e3, RT: 11.83 min
5.00 pg
11.83
S2 - 15-HETE[1] (Unknown) 319.2 / 219.1 - 210715 Para LOD.wiff (...
Area: 2.051e4, Height: 3.322e3, RT: 11.82 min
10.0 pg
11.82
S1 - 15-HETE[1] (Unknown) 319.2 / 219.1 - 210715 Para LOD.wiff (...
Area: 4.726e4, Height: 7.629e3, RT: 11.82 min
20.0 pg
11.82
Intensity
Time, min

11-HETE

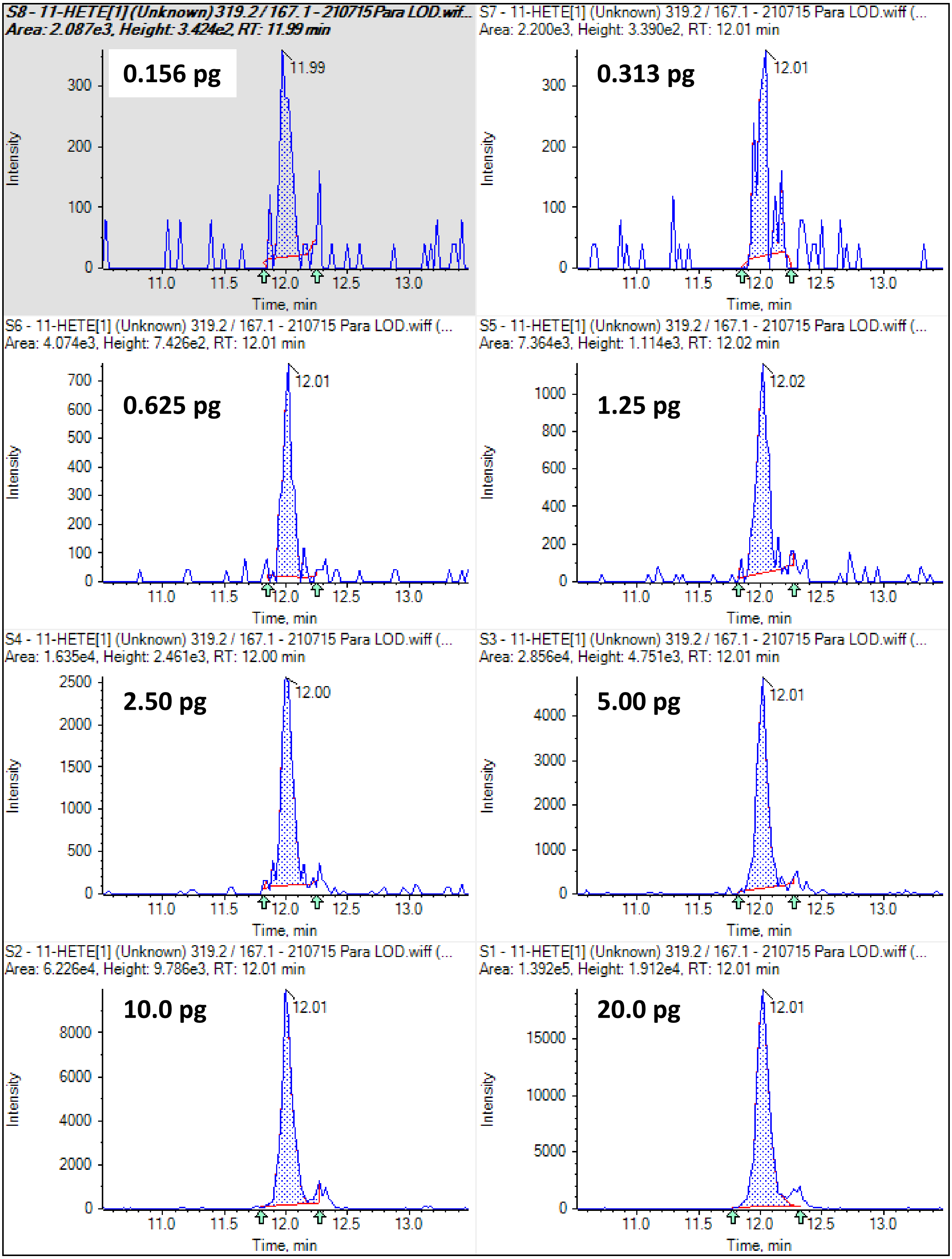
S8 - 11-HETE[1] (Unknown) 319.2 / 167.1 - 210715 Para LOD.wif...
Area: 2.087e3, Height: 3.424e2, RT: 11.99 min
0.156 pg
11.99
S7 - 11-HETE[1] (Unknown) 319.2 / 167.1 - 210715 Para LOD.wiff (...
Area: 2.200e3, Height: 3.390e2, RT: 12.01 min
0.313 pg
12.01
S6 - 11-HETE[1] (Unknown) 319.2 / 167.1 - 210715 Para LOD.wiff (...
Area: 4.074e3, Height: 7.426e2, RT: 12.01 min
0.625 pg
12.01
S5 - 11-HETE[1] (Unknown) 319.2 / 167.1 - 210715 Para LOD.wiff (...
Area: 7.364e3, Height: 1.114e3, RT: 12.02 min
1.25 pg
12.02
S4 - 11-HETE[1] (Unknown) 319.2 / 167.1 - 210715 Para LOD.wiff (...
Area: 1.635e4, Height: 2.461e3, RT: 12.00 min
2.50 pg
12.00
S3 - 11-HETE[1] (Unknown) 319.2 / 167.1 - 210715 Para LOD.wiff (...
Area: 2.856e4, Height: 4.751e3, RT: 12.01 min
5.00 pg
12.01
S2 - 11-HETE[1] (Unknown) 319.2 / 167.1 - 210715 Para LOD.wiff (...
Area: 6.226e4, Height: 9.786e3, RT: 12.01 min
10.0 pg
12.01
S1 - 11-HETE[1] (Unknown) 319.2 / 167.1 - 210715 Para LOD.wiff (...
Area: 1.392e5, Height: 1.912e4, RT: 12.01 min
20.0 pg
12.01
Intensity
Time, min
11.0
11.5
12.0
12.5
13.0

8-HETE

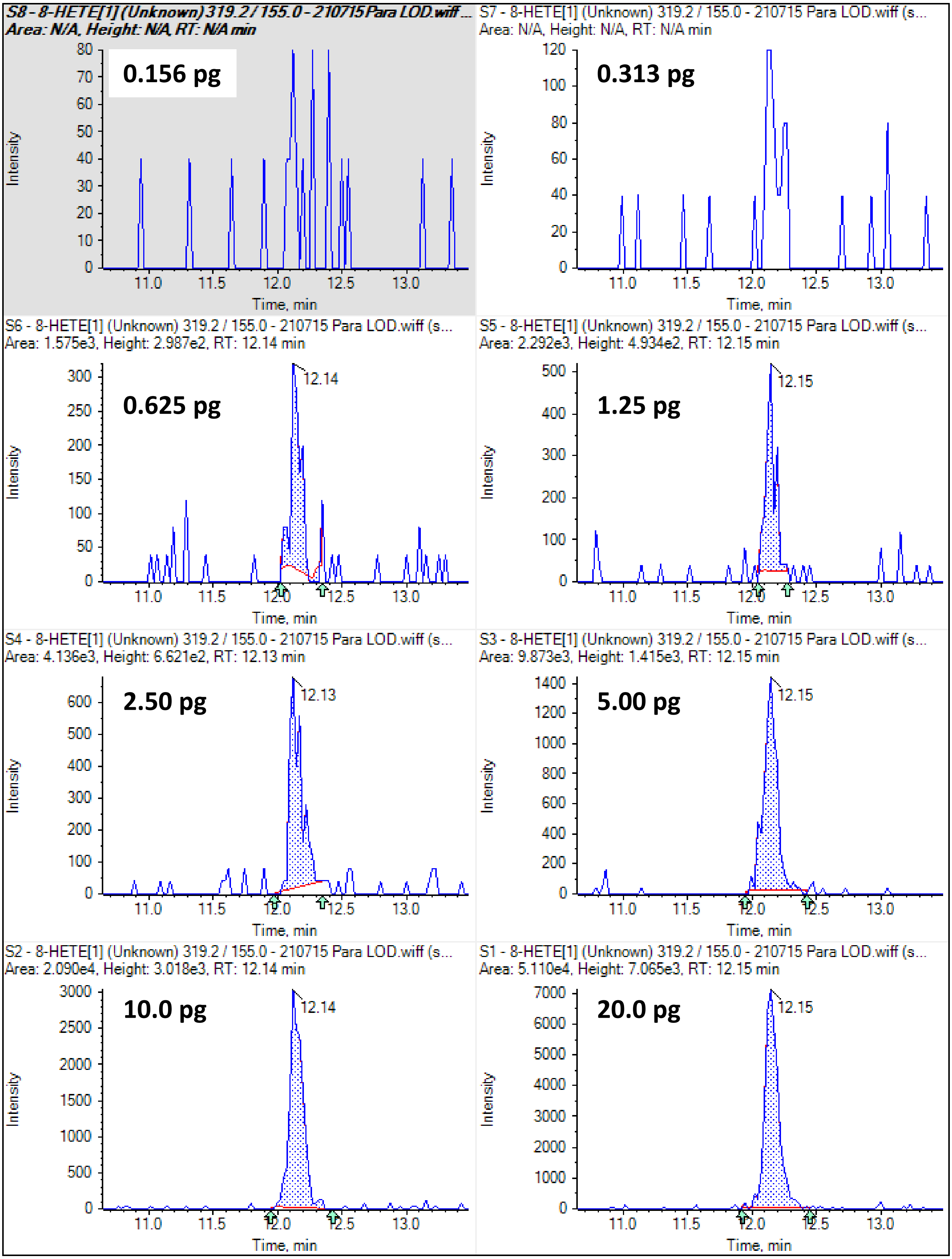

S8 - 8-HETE[1] (Unknown) 319.2 / 155.0 - 210715 Para LOD.wiff ...
Area: N/A, Height: N/A, RT: N/A min
0.156 pg
S7 - 8-HETE[1] (Unknown) 319.2 / 155.0 - 210715 Para LOD.wiff (s...
Area: N/A, Height: N/A, RT: N/A min
0.313 pg
S6 - 8-HETE[1] (Unknown) 319.2 / 155.0 - 210715 Para LOD.wiff (s...
Area: 1.575e3, Height: 2.987e2, RT: 12.14 min
0.625 pg
12.14
S5 - 8-HETE[1] (Unknown) 319.2 / 155.0 - 210715 Para LOD.wiff (s...
Area: 2.292e3, Height: 4.934e2, RT: 12.15 min
1.25 pg
12.15
S4 - 8-HETE[1] (Unknown) 319.2 / 155.0 - 210715 Para LOD.wiff (s...
Area: 4.136e3, Height: 6.621e2, RT: 12.13 min
2.50 pg
12.13
S3 - 8-HETE[1] (Unknown) 319.2 / 155.0 - 210715 Para LOD.wiff (s...
Area: 9.873e3, Height: 1.415e3, RT: 12.15 min
5.00 pg
12.15
S2 - 8-HETE[1] (Unknown) 319.2 / 155.0 - 210715 Para LOD.wiff (s...
Area: 2.090e4, Height: 3.018e3, RT: 12.14 min
10.0 pg
12.14
S1 - 8-HETE[1] (Unknown) 319.2 / 155.0 - 210715 Para LOD.wiff (s...
Area: 5.110e4, Height: 7.065e3, RT: 12.15 min
20.0 pg
12.15
Intensity
Time, min

9-HETE

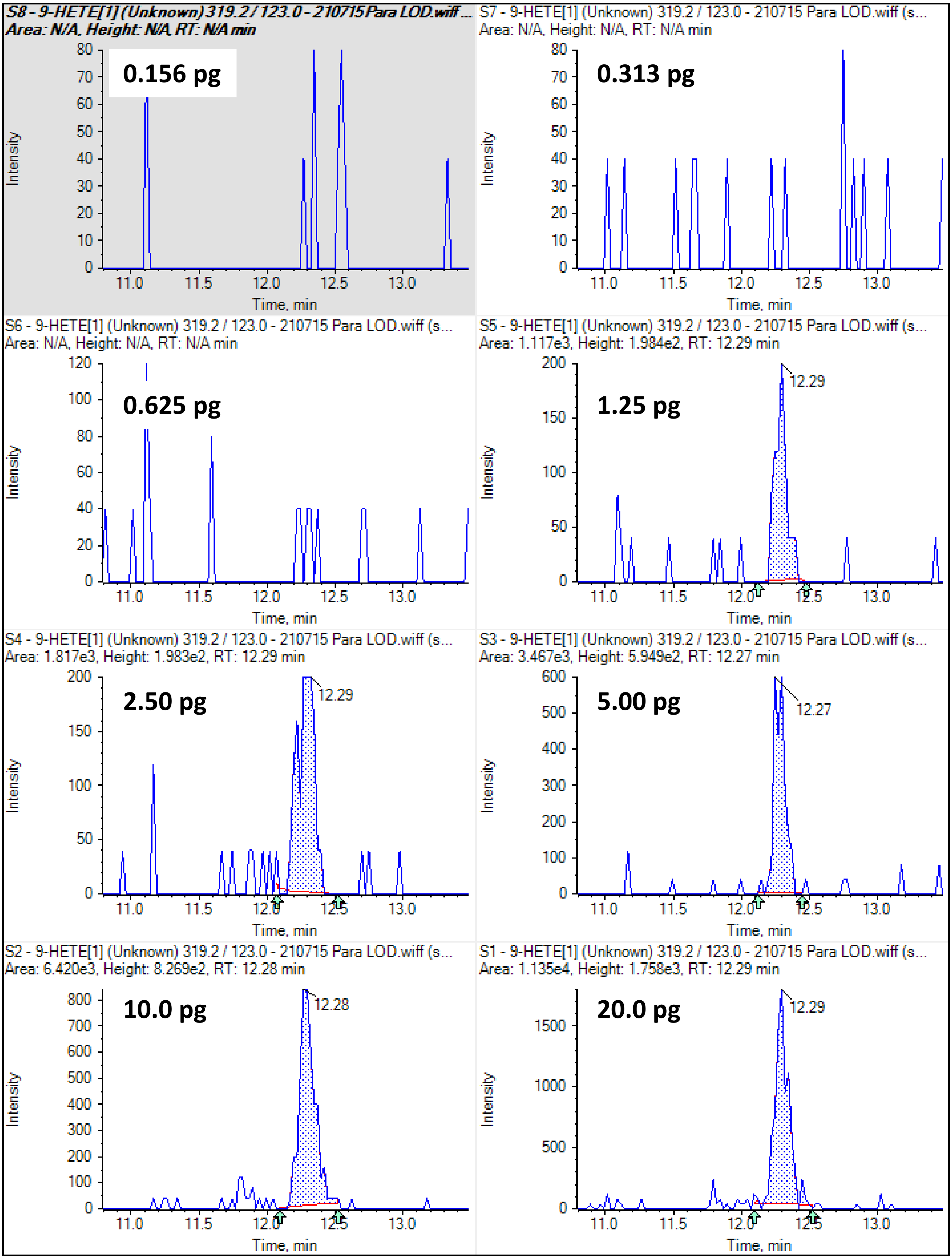
S8 - 9-HETE[1] (Unknown) 319.2 / 123.0 - 210715 Para LOD.wiff ...
Area: N/A, Height: N/A, RT: N/A min
0.156 pg
S7 - 9-HETE[1] (Unknown) 319.2 / 123.0 - 210715 Para LOD.wiff (s...
Area: N/A, Height: N/A, RT: N/A min
0.313 pg
S6 - 9-HETE[1] (Unknown) 319.2 / 123.0 - 210715 Para LOD.wiff (s...
Area: N/A, Height: N/A, RT: N/A min
0.625 pg
S5 - 9-HETE[1] (Unknown) 319.2 / 123.0 - 210715 Para LOD.wiff (s...
Area: 1.117e3, Height: 1.984e2, RT: 12.29 min
1.25 pg
12.29
S4 - 9-HETE[1] (Unknown) 319.2 / 123.0 - 210715 Para LOD.wiff (s...
Area: 1.817e3, Height: 1.983e2, RT: 12.29 min
2.50 pg
12.29
S3 - 9-HETE[1] (Unknown) 319.2 / 123.0 - 210715 Para LOD.wiff (s...
Area: 3.467e3, Height: 5.949e2, RT: 12.27 min
5.00 pg
12.27
S2 - 9-HETE[1] (Unknown) 319.2 / 123.0 - 210715 Para LOD.wiff (s...
Area: 6.420e3, Height: 8.269e2, RT: 12.28 min
10.0 pg
12.28
S1 - 9-HETE[1] (Unknown) 319.2 / 123.0 - 210715 Para LOD.wiff (s...
Area: 1.135e4, Height: 1.758e3, RT: 12.29 min
20.0 pg
12.29
Intensity
Time, min
11.0
11.5
12.0
12.5
13.0

12-HETE

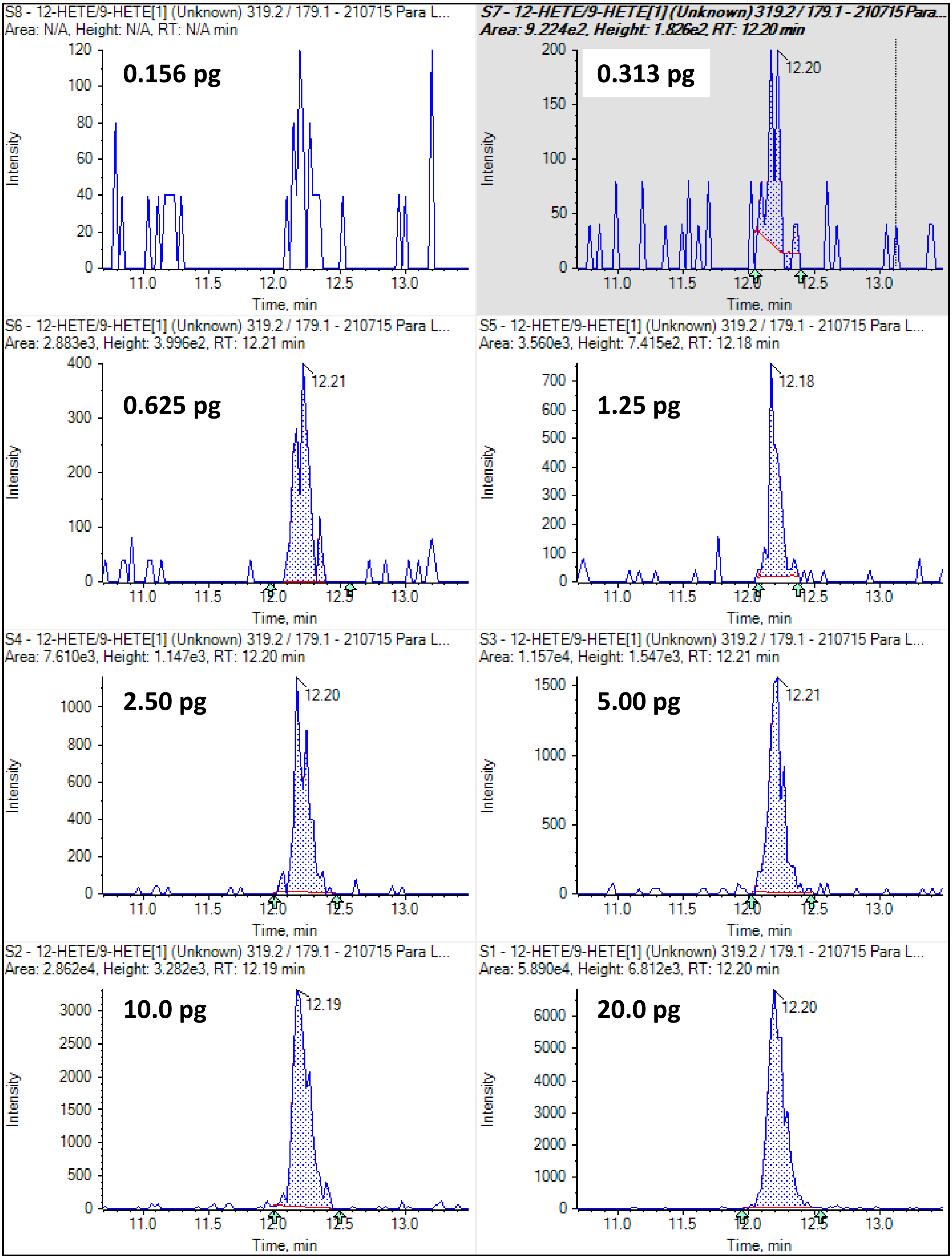

S8 - 12-HETE/9-HETE[1] (Unknown) 319.2 / 179.1 - 210715 Para L...
Area: N/A, Height: N/A, RT: N/A min
0.156 pg
S7 - 12-HETE/9-HETE[1] (Unknown) 319.2 / 179.1 - 210715 Para...
Area: 9.224e2, Height: 1.826e2, RT: 12.20 min
0.313 pg
S6 - 12-HETE/9-HETE[1] (Unknown) 319.2 / 179.1 - 210715 Para L...
Area: 2.883e3, Height: 3.996e2, RT: 12.21 min
0.625 pg
S5 - 12-HETE/9-HETE[1] (Unknown) 319.2 / 179.1 - 210715 Para L...
Area: 3.560e3, Height: 7.415e2, RT: 12.18 min
1.25 pg
S4 - 12-HETE/9-HETE[1] (Unknown) 319.2 / 179.1 - 210715 Para L...
Area: 7.610e3, Height: 1.147e3, RT: 12.20 min
2.50 pg
S3 - 12-HETE/9-HETE[1] (Unknown) 319.2 / 179.1 - 210715 Para L...
Area: 1.157e4, Height: 1.547e3, RT: 12.21 min
5.00 pg
S2 - 12-HETE/9-HETE[1] (Unknown) 319.2 / 179.1 - 210715 Para L...
Area: 2.862e4, Height: 3.282e3, RT: 12.19 min
10.0 pg
S1 - 12-HETE/9-HETE[1] (Unknown) 319.2 / 179.1 - 210715 Para L...
Area: 5.890e4, Height: 6.812e3, RT: 12.20 min
20.0 pg
Intensity
Time, min

5-HETE

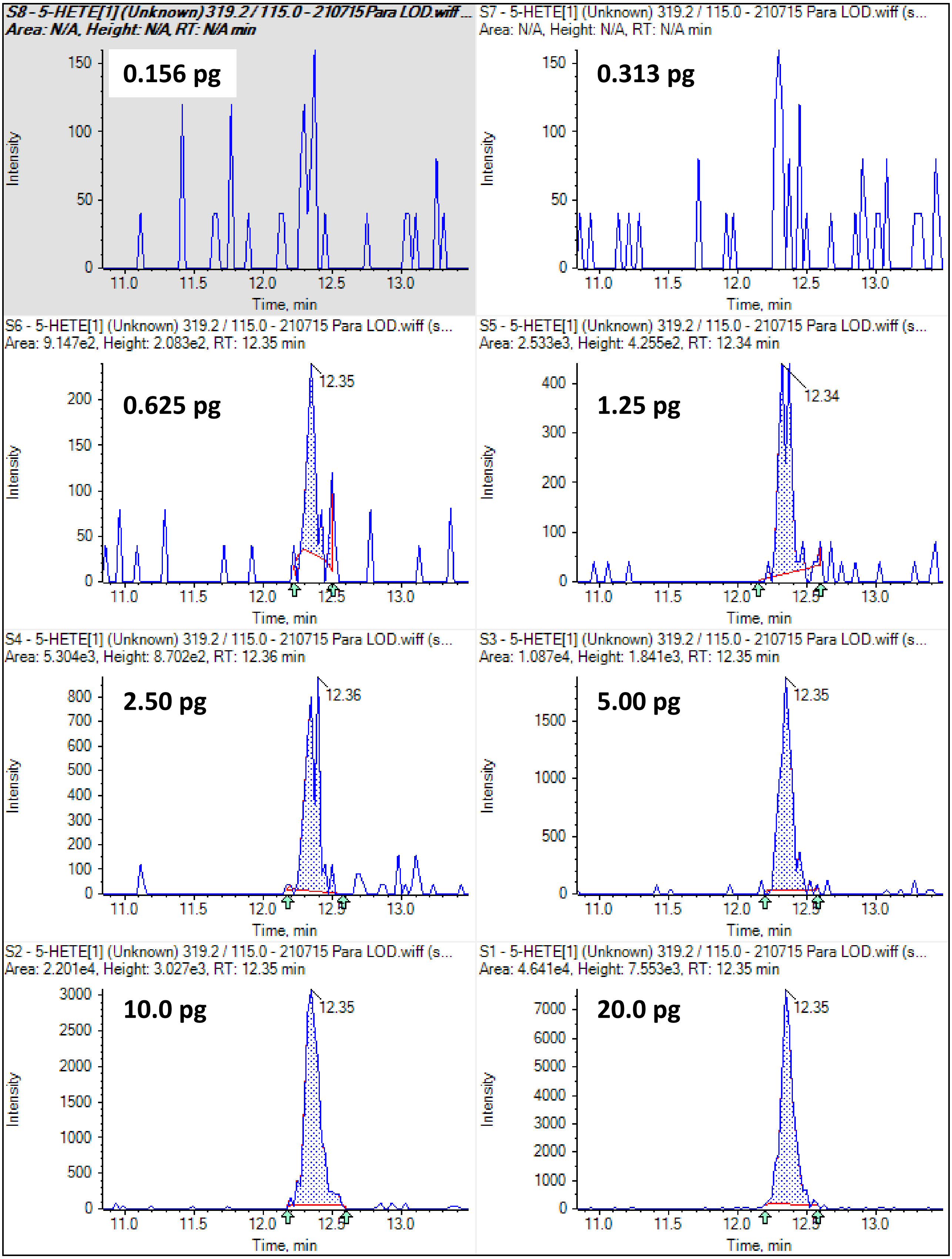
S8 - 5-HETE[1] (Unknown) 319.2 / 115.0 - 210715 Para LOD.wiff ...
Area: N/A, Height: N/A, RT: N/A min
0.156 pg
S7 - 5-HETE[1] (Unknown) 319.2 / 115.0 - 210715 Para LOD.wiff (s...
Area: N/A, Height: N/A, RT: N/A min
0.313 pg
S6 - 5-HETE[1] (Unknown) 319.2 / 115.0 - 210715 Para LOD.wiff (s...
Area: 9.147e2, Height: 2.083e2, RT: 12.35 min
0.625 pg
S5 - 5-HETE[1] (Unknown) 319.2 / 115.0 - 210715 Para LOD.wiff (s...
Area: 2.533e3, Height: 4.255e2, RT: 12.34 min
1.25 pg
S4 - 5-HETE[1] (Unknown) 319.2 / 115.0 - 210715 Para LOD.wiff (s...
Area: 5.304e3, Height: 8.702e2, RT: 12.36 min
2.50 pg
S3 - 5-HETE[1] (Unknown) 319.2 / 115.0 - 210715 Para LOD.wiff (s...
Area: 1.087e4, Height: 1.841e3, RT: 12.35 min
5.00 pg
S2 - 5-HETE[1] (Unknown) 319.2 / 115.0 - 210715 Para LOD.wiff (s...
Area: 2.201e4, Height: 3.027e3, RT: 12.35 min
10.0 pg
S1 - 5-HETE[1] (Unknown) 319.2 / 115.0 - 210715 Para LOD.wiff (s...
Area: 4.641e4, Height: 7.553e3, RT: 12.35 min
20.0 pg
Intensity
Time, min

16-HDoHE

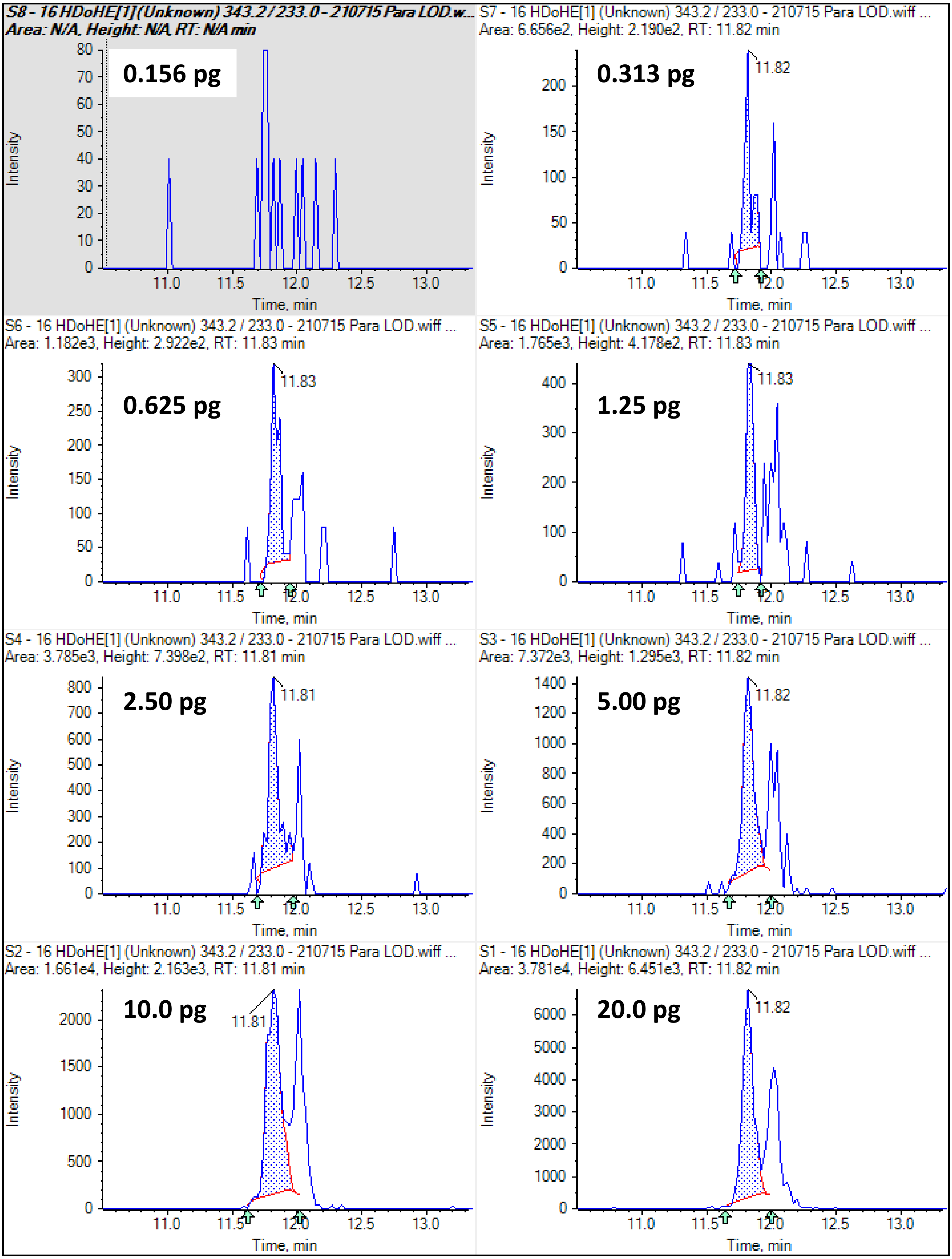

S8 - 16 HDoHE[1](Unknown) 343.2 / 233.0 - 210715 Para LOD.w...
Area: N/A, Height: N/A, RT: N/A min
0.156 pg
S7 - 16 HDoHE[1] (Unknown) 343.2 / 233.0 - 210715 Para LOD.wiff ...
Area: 6.656e2, Height: 2.190e2, RT: 11.82 min
0.313 pg
S6 - 16 HDoHE[1] (Unknown) 343.2 / 233.0 - 210715 Para LOD.wiff ...
Area: 1.182e3, Height: 2.922e2, RT: 11.83 min
0.625 pg
S5 - 16 HDoHE[1] (Unknown) 343.2 / 233.0 - 210715 Para LOD.wiff ...
Area: 1.765e3, Height: 4.178e2, RT: 11.83 min
1.25 pg
S4 - 16 HDoHE[1] (Unknown) 343.2 / 233.0 - 210715 Para LOD.wiff ...
Area: 3.785e3, Height: 7.398e2, RT: 11.81 min
2.50 pg
S3 - 16 HDoHE[1] (Unknown) 343.2 / 233.0 - 210715 Para LOD.wiff ...
Area: 7.372e3, Height: 1.295e3, RT: 11.82 min
5.00 pg
S2 - 16 HDoHE[1] (Unknown) 343.2 / 233.0 - 210715 Para LOD.wiff ...
Area: 1.661e4, Height: 2.163e3, RT: 11.81 min
10.0 pg
S1 - 16 HDoHE[1] (Unknown) 343.2 / 233.0 - 210715 Para LOD.wiff ...
Area: 3.781e4, Height: 6.451e3, RT: 11.82 min
20.0 pg
Intensity
Time, min

14-HDoHE

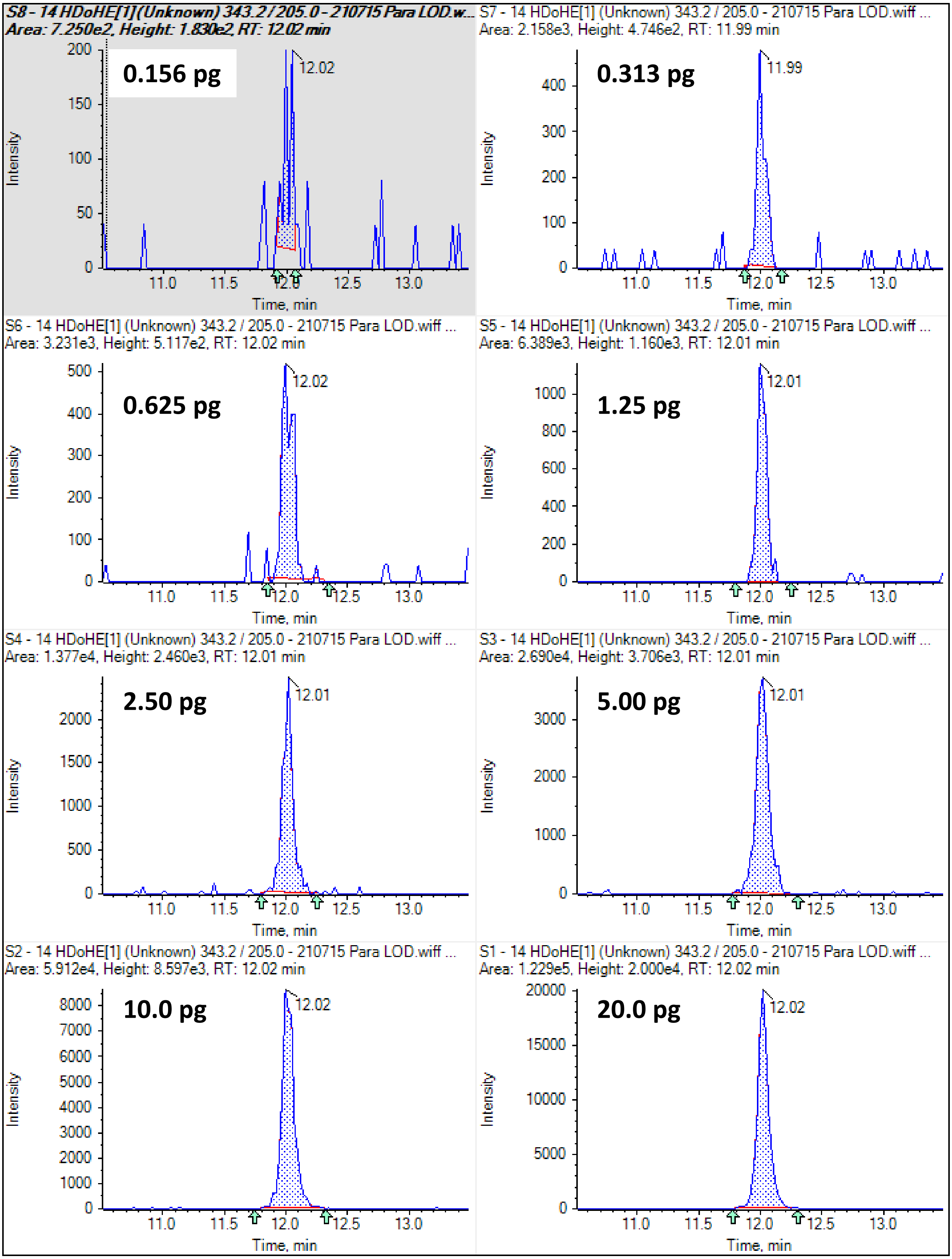

S8 - 14 HDoHE[1](Unknown) 343.2 / 205.0 - 210715 Para LOD.w...
Area: 7.250e2, Height: 1.830e2, RT: 12.02 min
0.156 pg
12.02
S7 - 14 HDoHE[1] (Unknown) 343.2 / 205.0 - 210715 Para LOD.wiff ...
Area: 2.158e3, Height: 4.746e2, RT: 11.99 min
0.313 pg
11.99
S6 - 14 HDoHE[1] (Unknown) 343.2 / 205.0 - 210715 Para LOD.wiff ...
Area: 3.231e3, Height: 5.117e2, RT: 12.02 min
0.625 pg
12.02
S5 - 14 HDoHE[1] (Unknown) 343.2 / 205.0 - 210715 Para LOD.wiff ...
Area: 6.389e3, Height: 1.160e3, RT: 12.01 min
1.25 pg
12.01
S4 - 14 HDoHE[1] (Unknown) 343.2 / 205.0 - 210715 Para LOD.wiff ...
Area: 1.377e4, Height: 2.460e3, RT: 12.01 min
2.50 pg
12.01
S3 - 14 HDoHE[1] (Unknown) 343.2 / 205.0 - 210715 Para LOD.wiff ...
Area: 2.690e4, Height: 3.706e3, RT: 12.01 min
5.00 pg
12.01
S2 - 14 HDoHE[1] (Unknown) 343.2 / 205.0 - 210715 Para LOD.wiff ...
Area: 5.912e4, Height: 8.597e3, RT: 12.02 min
10.0 pg
12.02
S1 - 14 HDoHE[1] (Unknown) 343.2 / 205.0 - 210715 Para LOD.wiff ...
Area: 1.229e5, Height: 2.000e4, RT: 12.02 min
20.0 pg
12.02
Intensity
Time, min
11.0
11.5
12.0
12.5
13.0

7-HDoHE

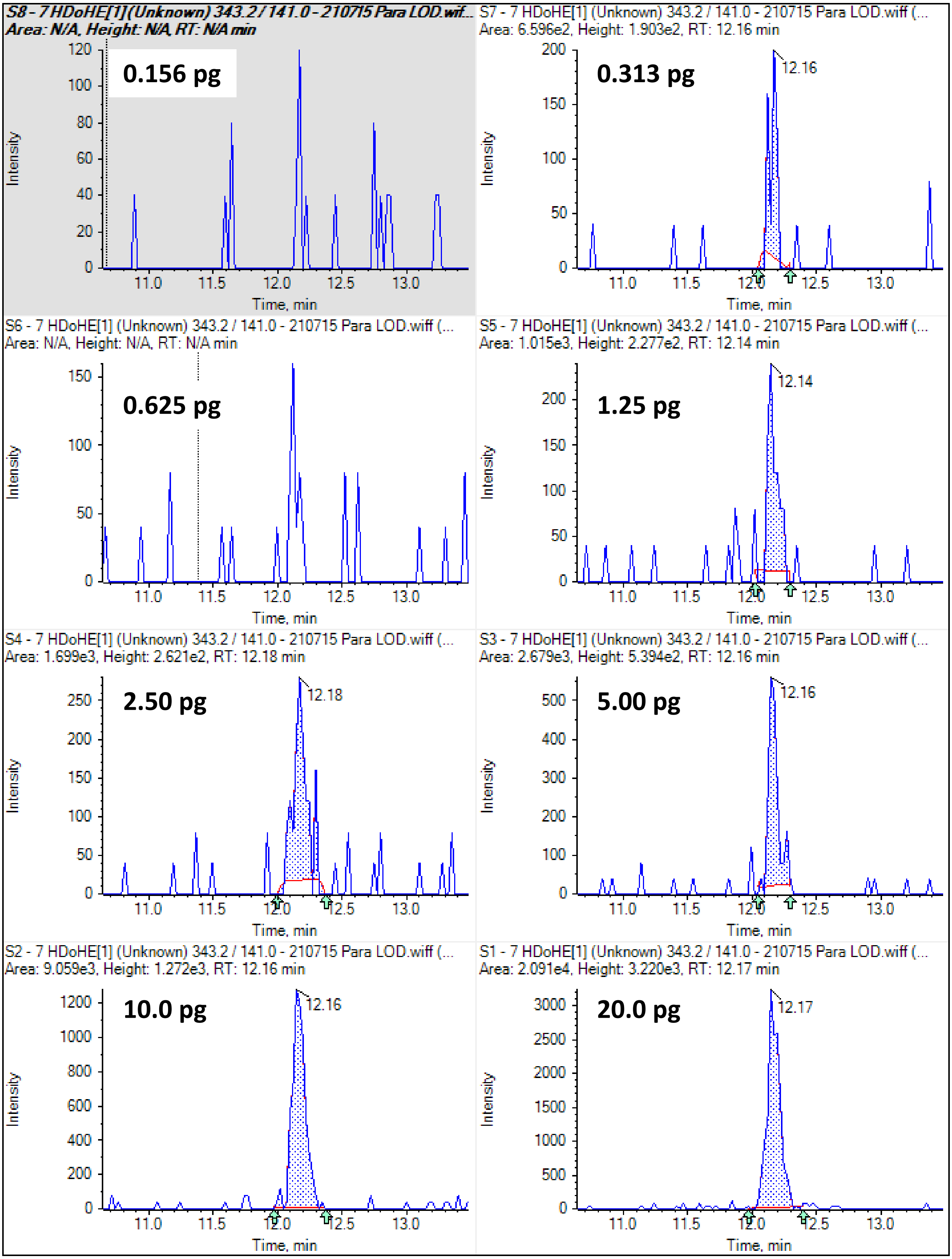

S8 - 7 HDoHE[1](Unknown) 343.2 / 141.0 - 210715 Para LOD.wif...
Area: N/A, Height: N/A, RT: N/A min
0.156 pg
S7 - 7 HDoHE[1] (Unknown) 343.2 / 141.0 - 210715 Para LOD.wiff (...
Area: 6.596e2, Height: 1.903e2, RT: 12.16 min
0.313 pg
12.16
S6 - 7 HDoHE[1] (Unknown) 343.2 / 141.0 - 210715 Para LOD.wiff (...
Area: N/A, Height: N/A, RT: N/A min
0.625 pg
S5 - 7 HDoHE[1] (Unknown) 343.2 / 141.0 - 210715 Para LOD.wiff (...
Area: 1.015e3, Height: 2.277e2, RT: 12.14 min
1.25 pg
12.14
S4 - 7 HDoHE[1] (Unknown) 343.2 / 141.0 - 210715 Para LOD.wiff (...
Area: 1.699e3, Height: 2.621e2, RT: 12.18 min
2.50 pg
12.18
S3 - 7 HDoHE[1] (Unknown) 343.2 / 141.0 - 210715 Para LOD.wiff (...
Area: 2.679e3, Height: 5.394e2, RT: 12.16 min
5.00 pg
12.16
S2 - 7 HDoHE[1] (Unknown) 343.2 / 141.0 - 210715 Para LOD.wiff (...
Area: 9.059e3, Height: 1.272e3, RT: 12.16 min
10.0 pg
12.16
S1 - 7 HDoHE[1] (Unknown) 343.2 / 141.0 - 210715 Para LOD.wiff (...
Area: 2.091e4, Height: 3.220e3, RT: 12.17 min
20.0 pg
12.17
Intensity
Time, min
11.0
11.5
12.0
12.5
13.0
