# Supplementary material for: HPLC-MS/MS Oxylipin Analysis of Plasma from Amyotrophic Lateral Sclerosis Patients
Source: Biomedicines. 2022 Mar 15;10(3):674. doi: 10.3390/biomedicines10030674 (PMC8945419; doi:10.3390/biomedicines10030674)

Figure S4. Supplementation experiment. Plasma samples were spiked with an SPM mix containing LXA4, LXB4, RvE1, RvD1, RvD2, RvD3, RvD5, MaR1, and PD1, and then regular analysis was performed. Chromatograms are shown in each of the following figures for all the SPM. The experiment was performed in duplicate at four different concentrations as indicated in each panel.

Plasmatic concentration

RvE1

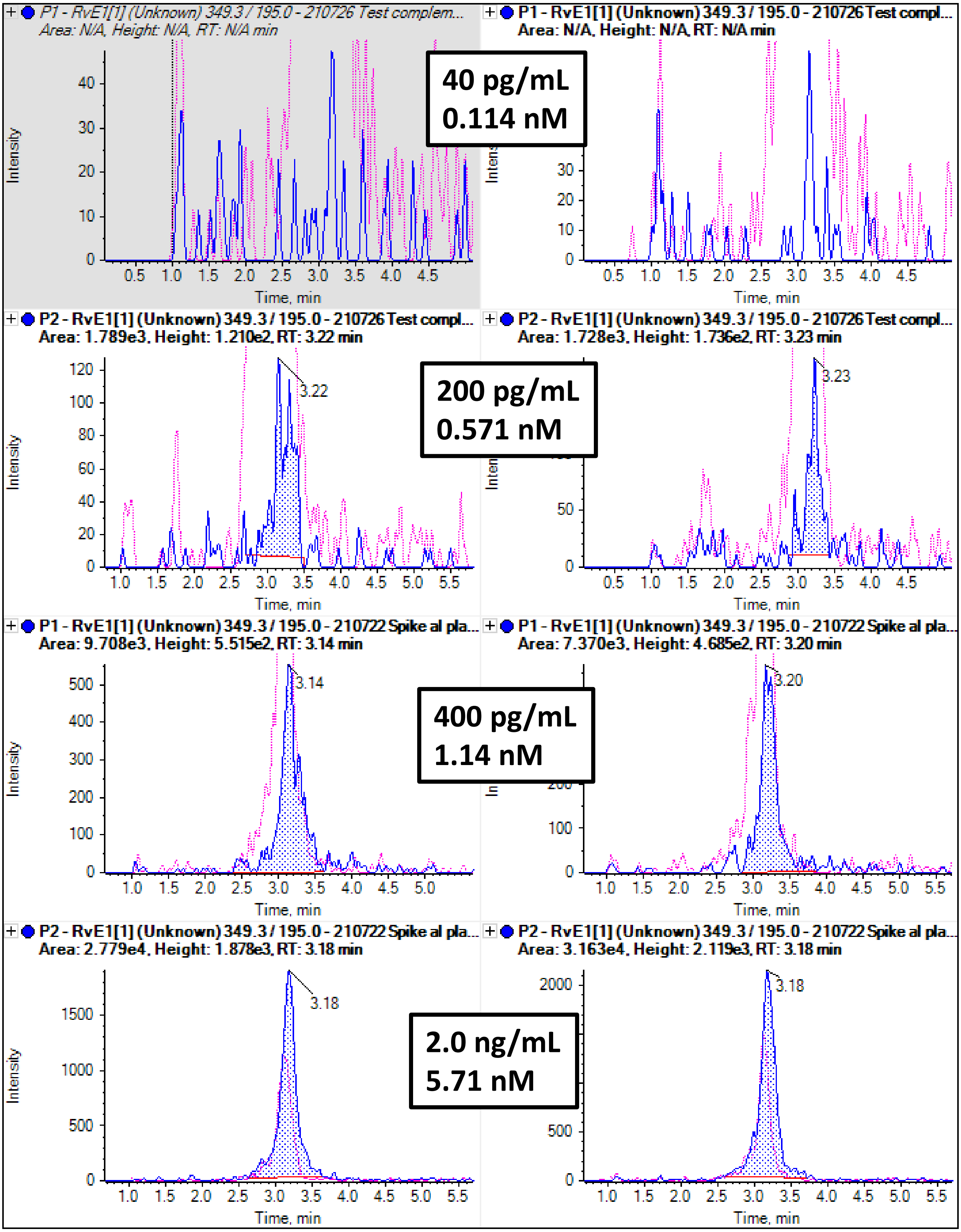

Plasmatic concentration

Mar1

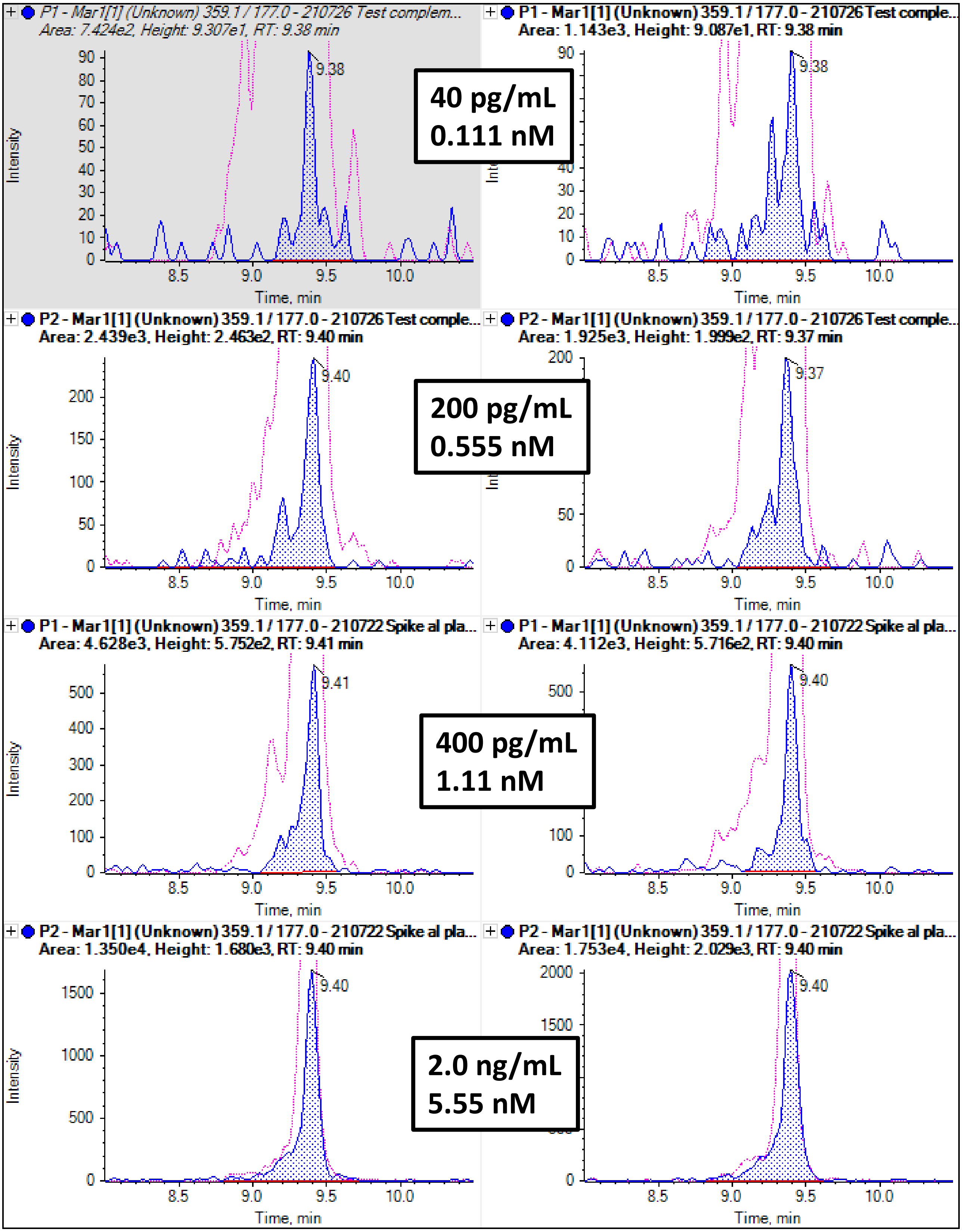

Plasmatic concentration

PD1

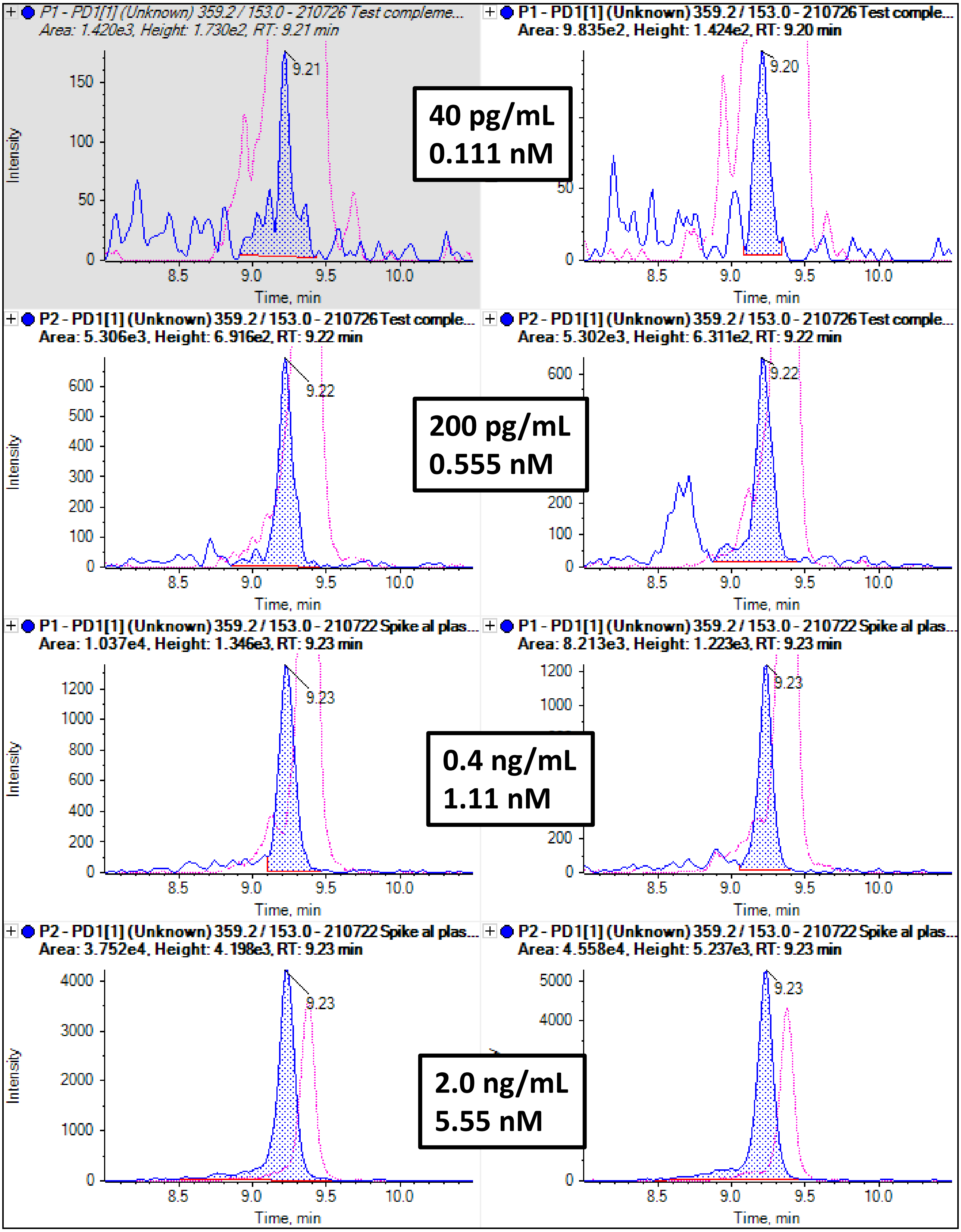

Plasmatic concentration

RvD1

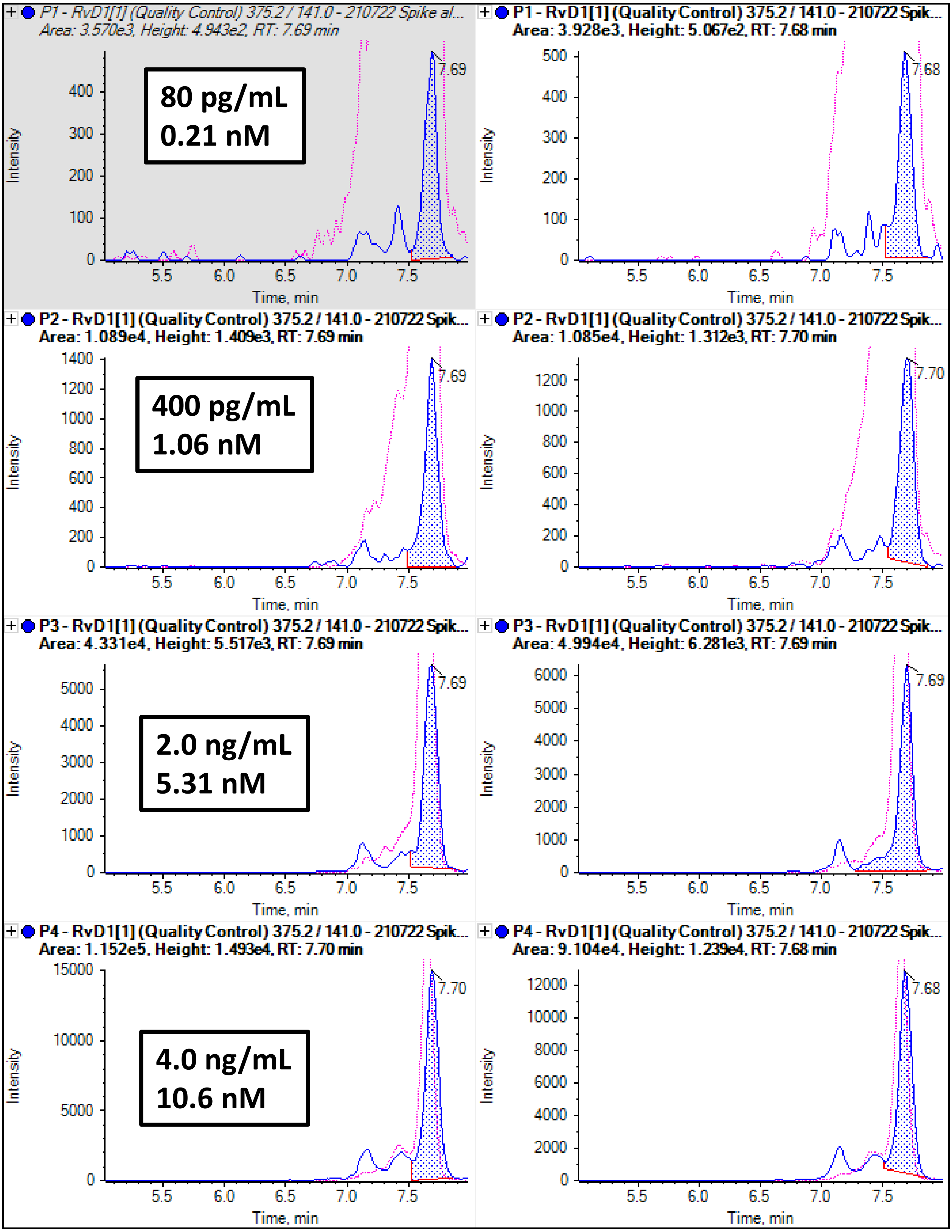

Plasmatic concentration

RvD2

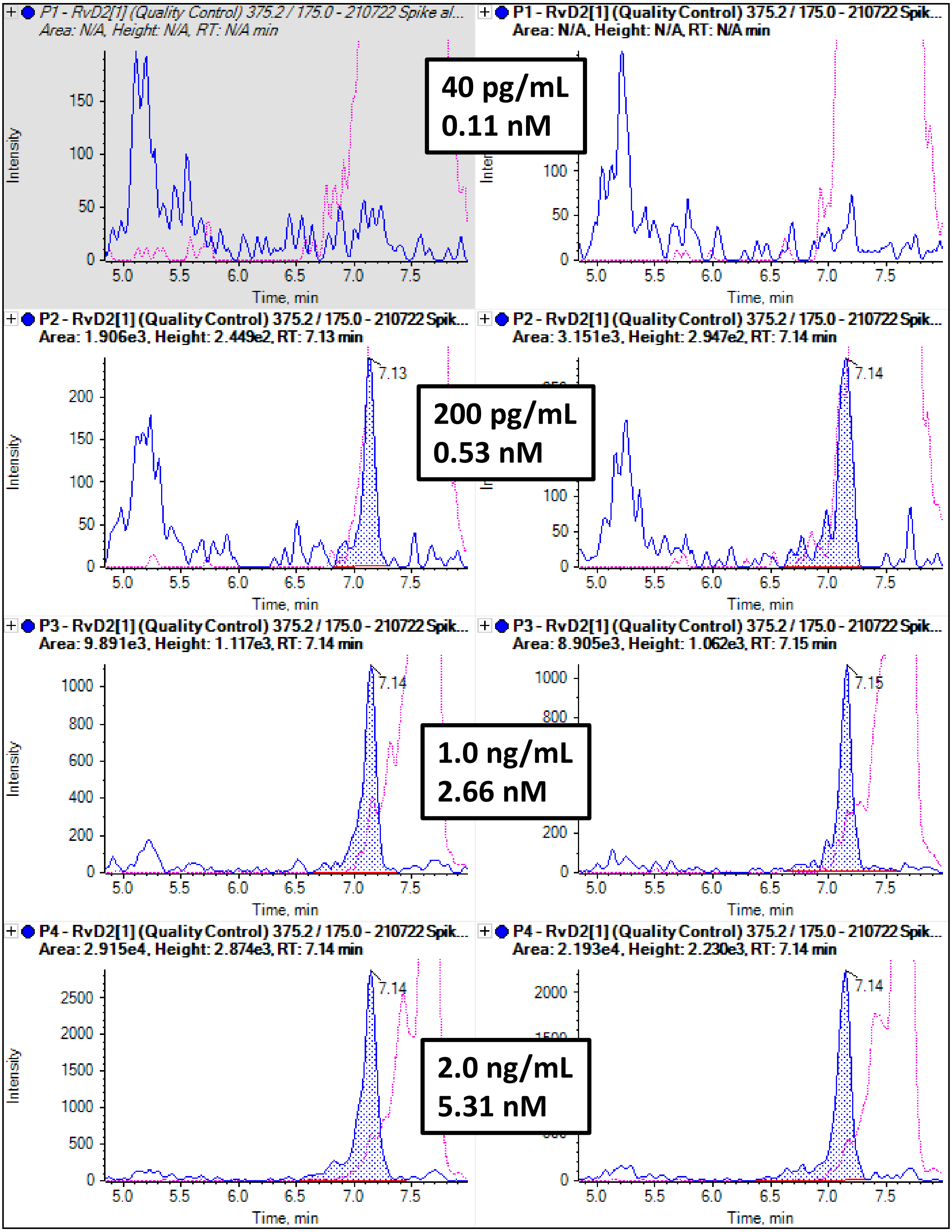

Plasmatic concentration

RvD3

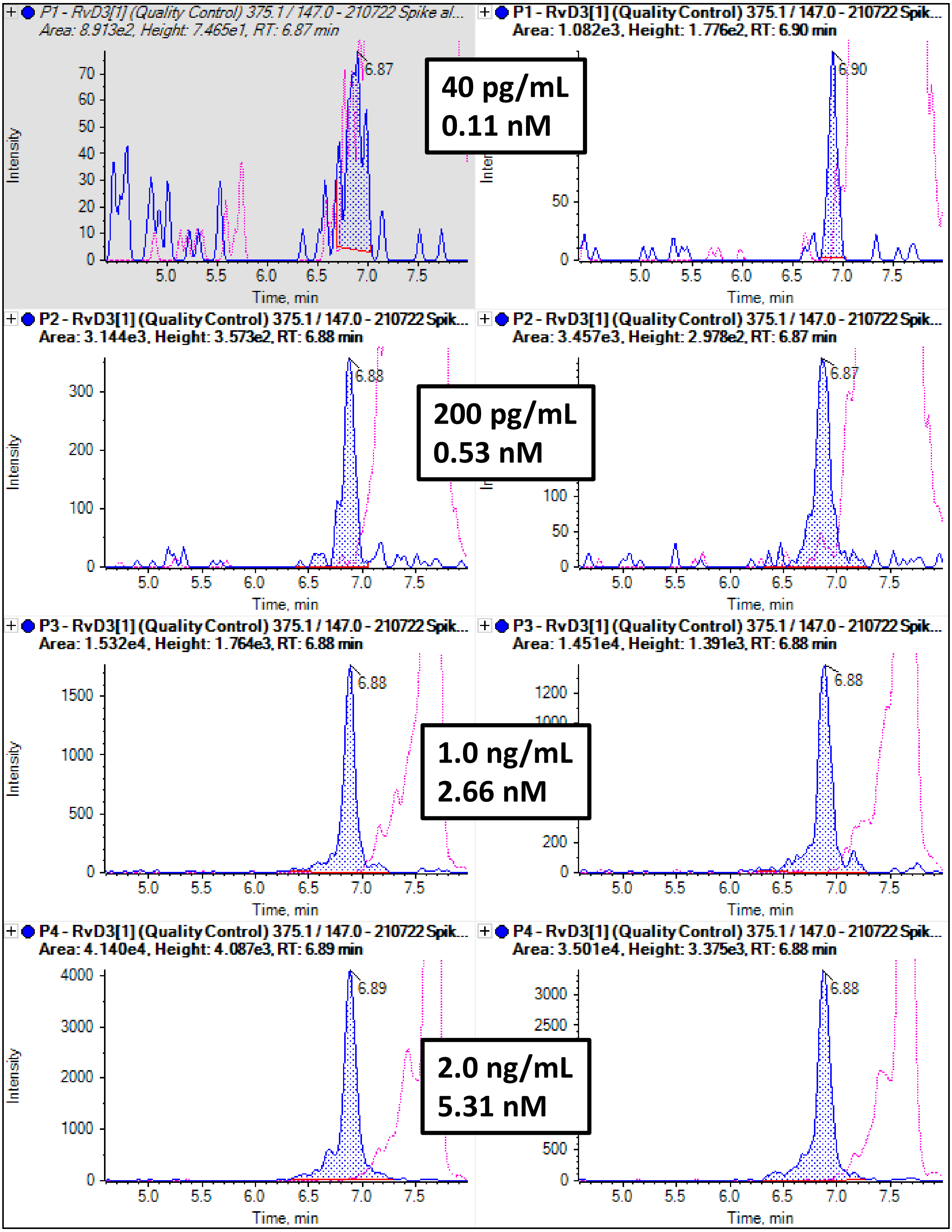

Plasmatic concentration

RvD5

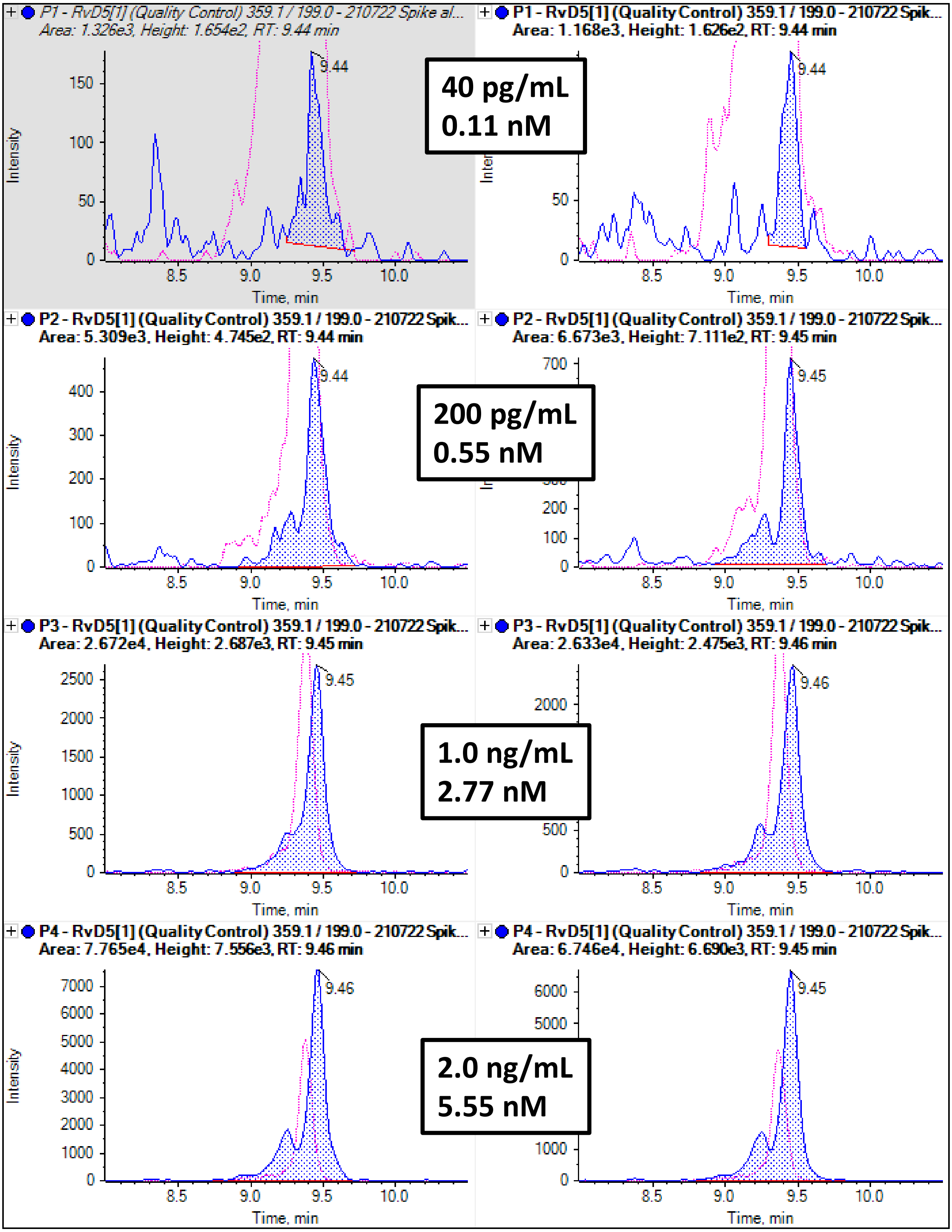

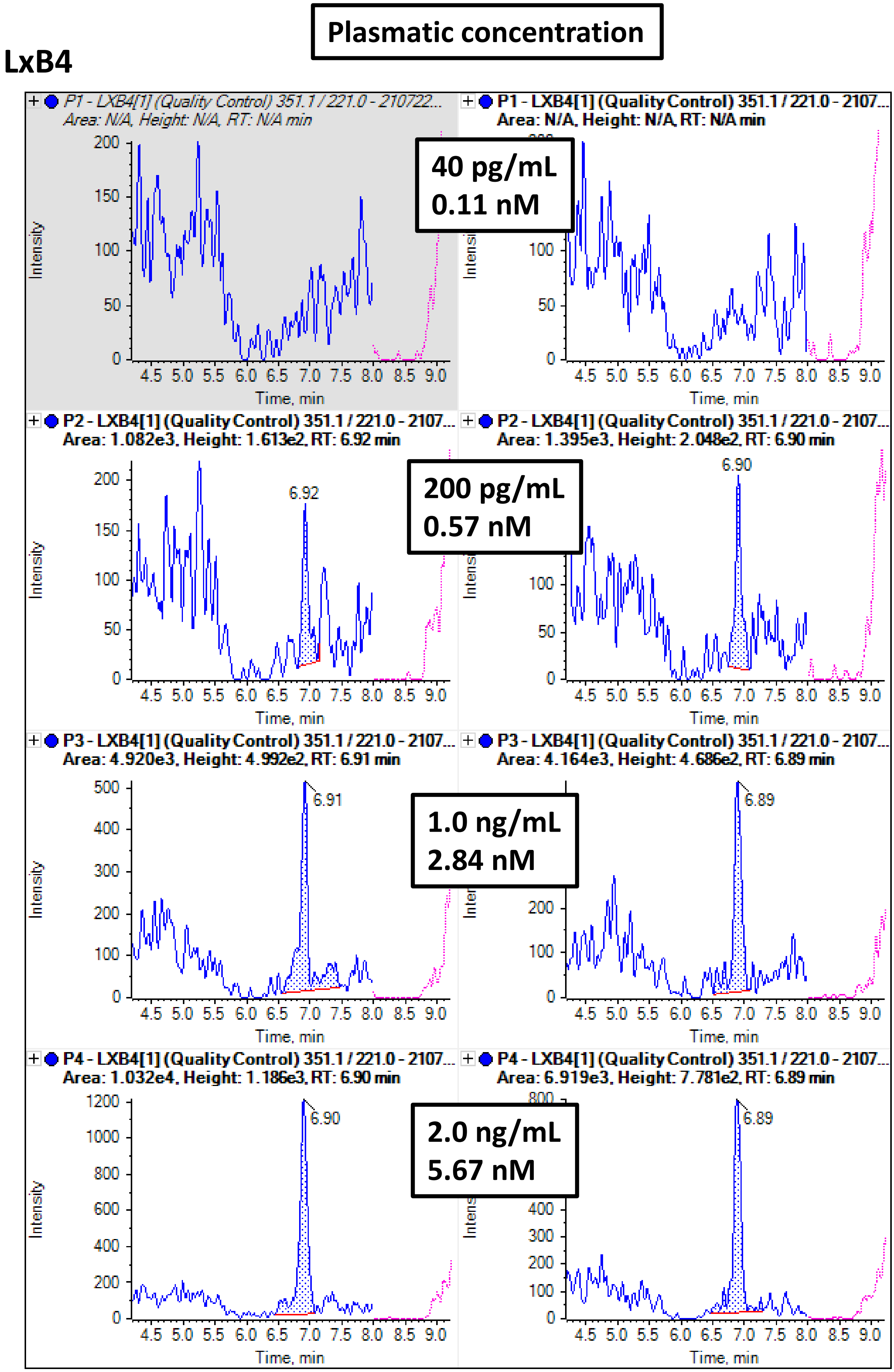

Plasmatic concentration
LxB4
P1 - LXB4[1] (Quality Control) 351.1 / 221.0 - 210722...
Area: N/A, Height: N/A, RT: N/A min
P1 - LXB4[1] (Quality Control) 351.1 / 221.0 - 2107...
Area: N/A, Height: N/A, RT: N/A min
40 pg/mL
0.11 nM
P2 - LXB4[1] (Quality Control) 351.1 / 221.0 - 2107...
Area: 1.082e3, Height: 1.613e2, RT: 6.92 min
P2 - LXB4[1] (Quality Control) 351.1 / 221.0 - 2107...
Area: 1.395e3, Height: 2.048e2, RT: 6.90 min
200 pg/mL
0.57 nM
P3 - LXB4[1] (Quality Control) 351.1 / 221.0 - 2107...
Area: 4.920e3, Height: 4.992e2, RT: 6.91 min
P3 - LXB4[1] (Quality Control) 351.1 / 221.0 - 2107...
Area: 4.164e3, Height: 4.686e2, RT: 6.89 min
1.0 ng/mL
2.84 nM
P4 - LXB4[1] (Quality Control) 351.1 / 221.0 - 2107...
Area: 1.032e4, Height: 1.186e3, RT: 6.90 min
P4 - LXB4[1] (Quality Control) 351.1 / 221.0 - 2107...
Area: 6.919e3, Height: 7.781e2, RT: 6.89 min
2.0 ng/mL
5.67 nM
Intensity
Time, min

LxA4

Plasmatic concentration

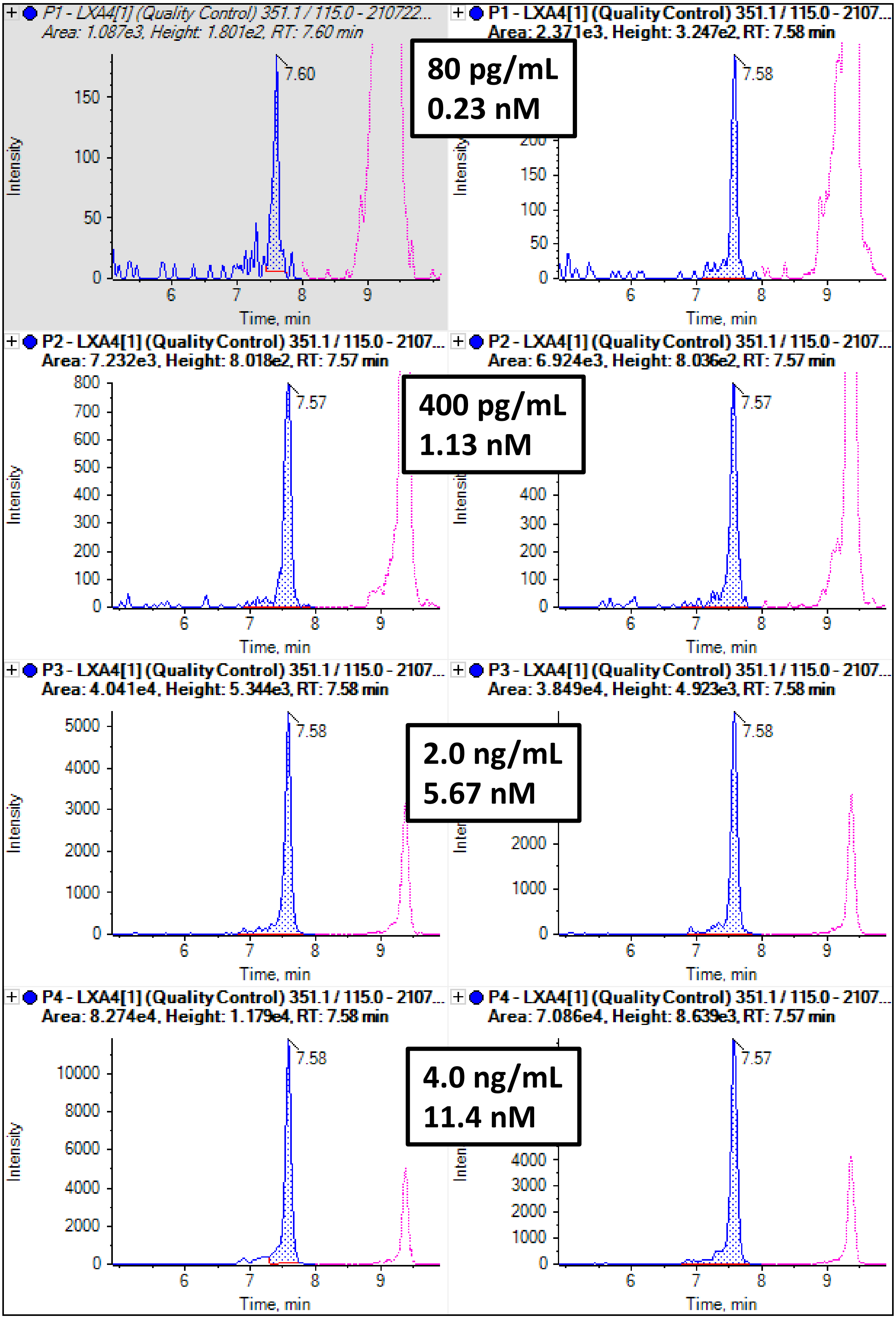

Supplement: Supplementary file 1 [file biomedicines-10-00674-s001.zip › Figure S4 - Supplementation experiment.pdf]
