# Supplementary material for: HPLC-MS/MS Oxylipin Analysis of Plasma from Amyotrophic Lateral Sclerosis Patients
Source: Biomedicines. 2022 Mar 15;10(3):674. doi: 10.3390/biomedicines10030674 (PMC8945419; doi:10.3390/biomedicines10030674)
Supplement: Supplementary file 1 [file biomedicines-10-00674-s001.zip › Table S1- Analyzed oxylipins.pdf]

Table S1. List of analyzed oxilipins in plasma samples by HPLC-MS/MS.

Abbreviation	Common name	Class	Fatty acid precursor	Pathway
**12,13-DiHOME**	12,13-dihydroxy-octadecenoic acid	diol	LA	CYP
**12,13-EpOME**	12(13)-epoxy-octadecenoic acid	epoxide	LA	CYP
**9,10-DiHOME**	9,10-dihydroxy-octadecenoic acid	diol	LA	CYP
**9,10-EpOME**	9(10)-epoxy-octadecenoic acid	epoxide	LA	CYP
**13-HODE**	13-hydroxy-octadecadienoic acid	alcohol	LA	LOX
**13-oxoODE**	13-oxo-octadecatrienoic acid	ketone	LA	LOX
**9-HODE**	9-hydroxy-octadecadienoic acid	alcohol	LA	LOX
**9-oxoODE**	9-oxo-octadecadienoic acid	ketone	LA	LOX
**13-HOTrE**	13-hydroxy-octadecatrienoic acid	alcohol	gLA	LOX
**9-HOTrE**	9-hydroxy-octadecatrienoic acid	alcohol	aLA	LOX
**AA**	Arachidonic acid	-	-	-
**6k PGF1α**	6-keto-Prostaglandin $F_{1\alpha}$	triol/ketone	AA	COX
**PGF2α**	Prostaglandin $F_{2\alpha}$	triol	AA	COX
**PGD2**	Prostaglandin D_2	diol/ketone	AA	COX
**PGE2**	Prostaglandin E_2	diol/ketone	AA	COX
**TxB2**	Thromboxane B_2	triol	AA	COX
**LTB4**	Leukotriene B4	diol	AA	LOX
**LXA4**	Lipoxin A_4	triol	AA	LOX
**LXB4**	Lipoxin B_4	triol	AA	LOX
**12-HETE**	12-hydroxy-eicosatetraenoic acid	alcohol	AA	LOX
**12-oxoETE**	12-oxo-eicosatetraenoic acid	ketone	AA	LOX
**15-HETE**	15-hydroxy-eicosatetraenoic acid	alcohol	AA	LOX
**15-oxoETE**	15-oxo-eicosatetraenoic acid	ketone	AA	LOX
**5-HETE**	5-hydroxy-eicosatetraenoic acid	alcohol	AA	LOX
**5-oxoETE**	5-oxo-eicosatetraenoic acid	ketone	AA	LOX
**8-HETE**	8-hydroxy-eicosatetraenoic acid	alcohol	AA	non-enz
**9-HETE**	9-hydroxy-eicosatetraenoic acid	alcohol	AA	non-enz
**11-HETE**	11-hydroxy-eicosatetraenoic acid	alcohol	AA	non-enz
**17-HETE**	17-hydroxy-eicosatetraenoic acid	alcohol	AA	CYP
**5-iso PGF2αVI**	Isoprostane $F_{2\alpha}$-IV	triol/ketone	AA	non-enz
**8-iso PGF2αIII**	Isoprostane $F_{2\alpha}$-III	triol/ketone	AA	non-enz
**14,15-diHETrE**	14,15-dihydroxy-eicosatrienoic acid	diol	AA	CYP
**11,12-diHETrE**	11,12-dihydroxy-eicosatrienoic acid	diol	AA	CYP
**8,9-diHETrE**	8,9-dihydroxy-eicosatrienoic acid	diol	AA	CYP
**5,6-diHETrE**	5,6-dihydroxy-eicosatrienoic acid	diol	AA	CYP
**EPA**	Eicosapentaenoic acid	-	-	-
**18-HEPE**	18-hydroxy-eicosapentaenoic acid	alcohol	EPA	CYP
**12-HEPE**	12-hydroxy-eicosapentaenoic acid	alcohol	EPA	LOX
**RvE1**	Resolvin E1	triol	EPA	LOX
**DHA**	Docohexaenoic acid	-	-	-
**PD1**	Protectin D1	diol	DHA	LOX
**MaR1**	Maresin 1	diol	DHA	LOX
**RvD1**	Resolvin D1	triol	DHA	LOX
**RvD2**	Resolvin D2	triol	DHA	LOX

**RvD3**	Resolvin D3	triol	DHA	LOX
**RvD5**	Resolvin D5	diol	DHA	LOX
**4-HDoHE**	4-hydroxy-docosahexaenoic acid	alcohol	DHA	LOX/non-enz
**7-HDoHE**	7-hydroxy-docosahexaenoic acid	alcohol	DHA	LOX/non-enz
**13-HDoHE**	13-hydroxy-docosahexaenoic Acid	alcohol	DHA	LOX/non-enz
**14-HDoHE**	14-hydroxy-docosahexaenoic acid	alcohol	DHA	LOX/non-enz
**16-HDoHE**	16-hydroxy-docosahexaenoic Acid	alcohol	DHA	non-enz
**20-HDoHE**	20-hydroxy-docosahexaenoic Acid	alcohol	DHA	non-enz
**19,20-DiHDPA**	19,20-dihydroxy-docosapentaenoic acid	diol	DHA	CYP
