# Supplementary material for: HPLC-MS/MS Oxylipin Analysis of Plasma from Amyotrophic Lateral Sclerosis Patients
Source: Biomedicines. 2022 Mar 15;10(3):674. doi: 10.3390/biomedicines10030674 (PMC8945419; doi:10.3390/biomedicines10030674)
Supplement: Supplementary file 1 [file biomedicines-10-00674-s001.zip › Table S2 - MRM list.pdf]

Table S2. Optimized parameters of the ESI-MS/MS method for the detection and quantification of oxylipins. This table shows all analytes and internal standards specific parameters; Precursor and Product ions (Q1 and Q3, respectively), dwell time, declustering potential (DP) and collision energy (CE). Ion source and other compound parameters were set as follows: CUR: 50; CAD: medium; IS: -4500 V; TEM: 400 °C; GS1: 40; GS2: 30; EP: -10; CXP: -13.

Q1	Q3	dwell	ID	DP	CE
373.5	249.1	35	(d4) 6k PGF1α[1]	-80	-39
373.5	211.0	5	(d4) 6k PGF1α[2]	-80	-34
373.5	293.1	5	(d4) 6k PGF1α[3]	-80	-29
373.5	167.0	5	(d4) 6k PGF1α[4]	-80	-34
369.2	245.0	35	6k PGF1α[1]	-80	-39
369.2	351.0	5	6k PGF1α[2]	-80	-39
369.2	315.0	5	6k PGF1α[3]	-80	-39
369.2	163.0	5	6k PGF1α[4]	-80	-39
353.3	197.0	35	(d4) RvE1[1]	-80	-25
349.3	195.0	35	RvE1[1]	-80	-25
349.3	161.0	5	RvE1[2]	-80	-30
349.3	205.1	5	RvE1[3]	-80	-22
349.3	143.0	5	RvE1[4]	-80	-24
373.2	173.0	35	(d4) TXB2[1]	-80	-26
373.2	199.1	5	(d4) TXB2[2]	-80	-22
373.2	293.1	5	(d4) TXB2[3]	-80	-21
373.2	211.0	5	(d4) TXB2[4]	-80	-27
369.2	169.1	35	TxB2[1]	-80	-26
369.2	325.0	5	TxB2[2]	-80	-11
369.2	195.1	5	TxB2[3]	-80	-22
369.2	177.1	5	TxB2[4]	-80	-30
357.3	197.0	35	(d4) PGF2α/(d4) 8-iso PGF2α VI[1]	-80	-35
357.3	313.2	5	(d4) PGF2α/(d4) 8-iso PGF2α VI[2]	-80	-28
357.3	295.0	5	(d4) PGF2α/(d4) 8-iso PGF2α VI[3]	-80	-28
364.4	115.0	35	(d11) 5-iso PGF2αVI[1]	-80	-30
364.4	346.3	5	(d11) 5-iso PGF2αVI[2]	-80	-25
364.4	320.2	5	(d11) 5-iso PGF2αVI[3]	-100	-30
353.2	193.1	35	PGF2α/8-iso PGF2α VI[1]	-80	-35
353.2	291.2	5	PGF2α/8-iso PGF2α VI[3]	-80	-28
353.2	182.9	5	PGF2α/8-iso PGF2α VI[4]	-80	-35
353.2	115.0	35	5-iso PGF2αVI[1]	-80	-30
353.2	335.3	5	5-iso PGF2αVI[2]	-80	-25
353.2	317.0	5	5-iso PGF2αVI[4]	-80	-20
355.5	275.2	35	(d4) PGD2/E2[1]	-80	-19
355.5	193.1	5	(d4) PGD2/E2[2]	-80	-23
355.5	239.1	5	(d4) PGD2/E2[3]	-80	-28
351.2	271.2	35	PGE2/PGD2[1]	-80	-19
351.2	315.2	5	PGE2/PGD2[2]	-80	-14
351.2	333.2	5	PGE2/PGD2[3]	-80	-13
351.2	189.1	5	PGE2/PGD2[4]	-80	-23
351.1	221.0	35	LXB4[1]	-50	-22
351.1	115.0	35	LXA4[1]	-50	-22
351.1	217.0	5	LXA4[2]	-50	-28
351.1	235.0	5	LXA4[3]	-50	-20
351.1	135.0	5	LXA4[4]	-50	-22

380.2	141.0	35	(d5) RvD1[1]	-80	-20
380.2	121.0	5	(d5) RvD1[2]	-80	-30
375.1	147.0	35	RvD3[1]	-80	-25
375.1	181.0	5	RvD3[2]	-80	-23
375.2	137.0	5	RvD3[3]	-80	-22
375.2	175.0	35	RvD2[1]	-80	-30
375.2	215.0	5	RvD1/RvD2[2]	-80	-28
375.2	141.0	35	RvD1/RvD2[1]	-80	-20
375.2	233.0	5	RvD1[2]	-80	-20
375.2	121.0	5	RvD1[3]	-80	-35
359.1	199.0	50	RvD5[1]	-80	-20
359.1	141.0	10	RvD5[2]	-80	-19
359.1	261.0	10	RvD5[3]	-80	-21
364.5	177.0	50	(d5) MaR1[1]	-80	-22
364.5	157.0	10	(d5) MaR1[2]	-80	-20
359.1	177.0	50	Mar1[1]	-80	-22
359.1	297.0	10	Mar1[2]	-80	-21
359.1	250.0	10	Mar1[3]	-80	-21
359.1	341.0	10	Mar1[4]	-80	-20
359.1	221.0	10	Mar1[5]	-80	-24
359.2	153.0	50	PD1[1]	-100	-21
359.2	206.0	10	PD1[2]	-100	-21
359.2	123.0	10	PD1[3]	-100	-20
335.2	195.0	50	LTB4[1]	-100	-23
335.2	317.1	10	LTB4[2]	-100	-19
335.2	129.0	10	LTB4[3]	-100	-28
335.2	151.0	10	LTB4[4]	-100	-27
313.2	183.0	50	12,13 diHOME[1]	-100	-29
313.2	195.0	10	12,13 diHOME[2]	-100	-29
313.2	201.0	50	9,10 diHOME[1]	-100	-29
313.2	171.0	10	9,10 diHOME[2]	-100	-30
313.2	295.0	10	12,13 diHOME / 9,10 diHOME[3]	-100	-26
313.2	277.0	10	12,13 diHOME / 9,10 diHOME[4]	-100	-29
293.2	275.0	10	HOTrEs[1]	-90	-22
293.2	231.0	10	HOTrEs[3]	-90	-26
337.5	207.0	50	14,15-diHETrE[1]	-90	-24
337.3	319.0	10	diHETrEs[1]	-90	-24
361.5	229	50	19,20 DiHDPA[1]	-90	-22
337.5	167.0	50	11,12-diHETrE[1]	-90	-25
337.5	127.0	50	8,9-diHETrE[1]	-90	-27
293.2	171.0	50	9-HOTrE[1]	-90	-22
361.5	343	10	19,20 DiHDPA[2]	-90	-22
361.5	281	10	19,20 DiHDPA[3]	-90	-22
317.1	299.1	25	HEPE[1]	-100	-19
293.2	275.0	25	HOTrEs[2]	-90	-22
293.2	195.0	25	13-HOTrE[1]	-90	-28
337.3	319.0	25	diHETrEs[1]	-90	-24
337.3	127.0	25	8,9-diHETrE[1]	-90	-27
337.3	145.1	25	5,6-diHETrE[1]	-90	-22
293.2	171.0	25	9-HOTrE[1]	-90	-22
317.1	299.1	25	HEPE[1]	-100	-19

317.1	215.0	25	18-HEPE[1]	-100	-20
317.1	259.0	5	18-HEPE[2]	-100	-20
317.1	179.0	25	12-HEPE[1]	-100	-19
317.1	135.0	5	12-HEPE[2]	-100	-20
299.2	172.0	25	(d4) 9-HODE[1]	-100	-22
299.2	198.2	25	(d4) 13-HODE[1]	-100	-25
299.2	281.3	2	(d4) 9-HODE/(d4) 13-HODE[2]	-100	-23
295.2	195.0	25	13-HODE/12,13 EpOME[1]	-100	-25
295.2	171.0	25	9-HODE/9,10 EpOME[1]	-100	-22
295.2	277.1	5	HODEs/EpOMEs[2]	-100	-23
293.0	195.0	25	13-oxoODE[1]	-100	-29
293.0	167.0	5	13-oxoODE[2]	-100	-27
293.0	185.0	25	9-oxoODE[1]	-100	-28
293.0	249.0	5	13-oxoODE/9-oxoODE[2]	-100	-26
327.3	309.2	25	(d8) HETEs[1]	-95	-19
327.3	226.0	25	(d8) 15-HETE[1]	-95	-18
327.3	182.0	2	(d8) 15-HETE[2]	-95	-24
327.3	139.1	2	(d8) 12-HETE[2]	-95	-22
327.3	184.0	25	(d8) 12-HETE[1]	-95	-20
327.3	116.0	25	(d8) 5-HETE[1]	-95	-20
327.3	210.0	2	(d8) 5-HETE[2]	-95	-24
319.2	301.1	25	HETEs[1]	-95	-19
319.2	257.1	5	HETEs[2]	-95	-20
319.2	247.0	25	17-HETE[1]	-95	-20
319.2	219.1	25	15-HETE[1]	-95	-18
319.2	175.2	5	15-HETE[2]	-95	-24
319.2	167.1	25	11-HETE[1]	-95	-23
319.2	195.0	5	11-HETE[2]	-95	-23
319.2	155.0	25	8-HETE[1]	-95	-22
319.2	163.0	5	8-HETE[2]	-95	-25
319.2	135.1	5	12-HETE[2]	-95	-22
319.2	123.0	25	9-HETE[1]	-95	-22
319.2	179.1	25	12-HETE/9-HETE[1]	-95	-20
319.2	115.0	25	5-HETE[1]	-95	-20
319.2	203.1	5	5-HETE/17-HETE[2]	-95	-24
317.5	299.4	25	oxoETEs[1]	-95	-22
317.5	273.1	5	oxoETEs[2]	-95	-20
317.5	113.0	25	15-oxoETE[1]	-95	-22
317.5	139.0	5	15-oxoETE[2]	-95	-28
317.5	153.0	25	12-oxoETE[1]	-95	-22
317.5	203.3	25	5-oxoETE[1]	-95	-24
317.5	245.4	5	5-oxoETE[2]	-95	-24
343.2	281.1	25	HDoHE[1]	-100	-19
343.2	325.1	5	HDoHE[2]	-100	-15
343.2	241	25	20 HDoHE[1]	-100	-18
343.2	233.0	25	16 HDoHE[1]	-100	-19
343.2	189.0	5	16 HDoHE[2]	-100	-21
343.2	193.0	25	13 HDoHE[1]	-100	-18
343.2	221.0	5	13 HDoHE[2]	-100	-18
343.2	205.0	25	14 HDoHE[1]	-100	-18
343.2	161.0	5	14 HDoHE[2]	-100	-19

343.2	153	25	10 HDoHE[1]	-100	-19
343.2	149	25	11 HDoHE[1]	-100	-19
343.2	141.0	25	7 HDoHE[1]	-100	-19
343.2	201.1	5	7 HDoHE[2]	-100	-20
343.2	101.0	25	4 HDoHE[1]	-100	-20
343.2	299.1	5	4 HDoHE[2]	-100	-18
317.5	203.3	50	5-oxoETE[1]	-95	-24
317.5	245.4	20	5-oxoETE[2]	-95	-24
311.3	267.0	50	(d8) AA[1]	-100	-20
311.3	183.0	20	(d8) AA[2]	-100	-19
303.1	259.0	50	AA[1]	-100	-20
303.1	205.0	20	AA[2]	-100	-20
327.1	283.2	50	DHA[1]	-95	-20
327.1	229.3	20	DHA[2]	-95	-20
327.1	309.6	20	DHA[3]	-95	-20
327.1	185.0	20	DHA[4]	-95	-50
301.3	257.2	50	EPA[1]	-95	-20
301.3	203.1	20	EPA[2]	-95	-20
301.3	283.1	20	EPA[3]	-95	-16
301.3	223.0	20	EPA[4]	-95	-16
