# Supplementary material for: HPLC-MS/MS Oxylipin Analysis of Plasma from Amyotrophic Lateral Sclerosis Patients
Source: Biomedicines. 2022 Mar 15;10(3):674. doi: 10.3390/biomedicines10030674 (PMC8945419; doi:10.3390/biomedicines10030674)
Supplement: Supplementary file 1 [file biomedicines-10-00674-s001.zip › Table S3 - Calibration curves.pdf]

Table S3. Calibration curves obtained for oxylipins analyzed by HPLC-MS/MS.

		Calibration curves				
**Analyte**	**Internal Standard**	**slope**	**offset**	**r**	**Range (pg)**	
12,13 diHOME	(d5) MaR1	31.28735	0.00461	0.99999	25	2500
12,13 EpOME	(d4)13-HODE	0.19826	1.08E-04	0.99974	25	2500
9,10 diHOME	(d5) MaR1	225.14566	1.06863	0.99886	25	1250
9,10 EpOME	(d4)9-HODE	0.26935	1.87E-04	0.99943	25	2500
13-HODE	(d4)13-HODE	2.09516	0.00354	0.99976	25	2500
13-oxoODE	(d4)13-HODE	0.24426	0.0012	0.99976	25	2500
9-HODE	(d4)9-HODE	1.08524	0.05323	0.99758	25	2500
9-oxoODE	(d4)9-HODE	0.56833	0.00417	0.99741	25	2500
AA	(d8) 5-HETE	0.04221	0.00541			
6k PGF1α	(d4) 6k PGF1α	2.37925	0.01705	0.99581	25	2500
PGF2α	(d4) PGF2α	2.22926	0.03296	0.99522	25	2500
PGD2	(d4) PGD2	6.92117	0.04975	0.99453	25	2500
PGE2	(d4) PGE2	4.15027	0.0201	0.99817	25	2500
TxB2	(d4) TxB2	1.95717	0.0109	0.99575	25	2500
LTB4	(d5) MaR1	7.53503	-0.02112	0.99970	25	2500
LXA4	(d5) MaR1	5.62113	-0.0023	0.99813	50	1000
LXB4	(d5) MaR1	1.95314	0.00523	0.99763	25	500
12-HETE	(d8) 12-HETE	1.88272	0.00637	0.99992	25	2500
12-oxoETE	(d8) 12-HETE	0.52842	-0.00159	0.99978	25	2500
15-HETE	(d8) 15-HETE	1.5209	0.00216	0.99998	25	2500
15-oxoETE	(d8) 15-HETE	0.6503	0.00305	0.99994	25	2500
5-HETE	(d8) 5-HETE	1.25232	0.00505	0.99990	25	2500
5-oxoETE	(d8) 5-HETE	0.22993	-2.72E-04	0.99989	25	2500
8-HETE	(d8) 12-HETE	1.35855	0.00645	0.99980	25	2500
9-HETE	(d8) 12-HETE	0.38928	0.00131	0.99995	25	2500
11-HETE	(d8) 15-HETE	4.16814	0.01445	0.99990	25	2500
18-HEPE	(d8) 15-HETE	0.46928	-0.00117	0.99953	25	2500
RvE1	(d4) RvE1	1.7233	0.00245	0.99944	25	250
DHA	(d8) 5-HETE	0.26642	0.01722			
PD1	(d5) MaR1	2.81574	-0.00558	0.99989	25	2500
MaR1	(d5) MaR1	1.40761	0.00367	0.99998	25	2500
RvD1	(d5) RvD1	0.58949	0.01048	0.99885	50	1000
RvD2	(d5) RvD1	0.30054	3.00E-04	0.99769	25	500
RvD3	(d5) RvD1	0.6528	0.00114	0.99851	25	500
RvD5	(d5) MaR1	4.17593	0.00295	0.99971	25	500
7 HDoHE	(d8) 5-HETE	0.67953	3.73E-04	0.99990	25	2500
14 HDoHE	(d8) 12-HETE	4.62304	-0.00544	0.99977	25	2500
16 HDoHE	(d8) 15-HETE	2.03503	-0.0075	0.99956	25	2500
