# Supplementary material for: HPLC-MS/MS Oxylipin Analysis of Plasma from Amyotrophic Lateral Sclerosis Patients
Source: Biomedicines. 2022 Mar 15;10(3):674. doi: 10.3390/biomedicines10030674 (PMC8945419; doi:10.3390/biomedicines10030674)
Supplement: Supplementary file 1 [file biomedicines-10-00674-s001.zip › Table S4 - Calibrants.pdf]

Table S4. Calibration curves used for analytes with no standard. Those compounds for which standards were not available were quantified employing calibration curves of the most similar molecules as calibrants.

Analyte	Calibrant
**13-HOTrE**	13-HODE
**9-HOTrE**	9-HODE
**17-HETE**	15-HETE
**5-iso PGF2αVI**	PGF2α
**8-iso PGF2αIII**	PGF2α
**14,15-diHETrE**	LTB4
**11,12-diHETrE**	MaR1
**8,9-diHETrE**	MaR1
**5,6-diHETrE**	MaR1
**EPA**	AA
**12-HEPE**	18-HEPE
**4 HDoHE**	7 HDoHE
**13 HDoHE**	14 HDoHE
**20 HDoHE**	16 HDoHE
**19,20 DiHDPA**	MaR1
