# Supplementary material for: HPLC-MS/MS Oxylipin Analysis of Plasma from Amyotrophic Lateral Sclerosis Patients
Source: Biomedicines. 2022 Mar 15;10(3):674. doi: 10.3390/biomedicines10030674 (PMC8945419; doi:10.3390/biomedicines10030674)
Supplement: Supplementary file 1 [file biomedicines-10-00674-s001.zip › Table S5 - General LOD & LOQ.pdf]

Table S5. Limits of detection and quantitation (LOD and LOQ, respectively) expressed as pg/mL in plasma determined from calibration curves and recovery rates from plasma sample processing.

Oxylipin	LOD (pg/mL plasma)	LOQ10 (pg/mL plasma)
6k PGF1α	1,10E+00	3,68E+00
RvE1	6,88E-01	2,29E+00
TxB2	1,03E-01	3,44E-01
PGF2α	2,74E+01	9,13E+01
8-iso PGF2α VI	2,89E+01	9,62E+01
5-iso PGF2αVI	5,17E+00	1,72E+01
PGE2	3,53E+00	1,18E+01
PGD2	9,75E-01	3,25E+00
LXB4	2,92E+00	9,72E+00
LXA4	5,77E-01	1,92E+00
RvD3	2,31E+00	7,69E+00
RvD2	1,36E+00	4,55E+00
RvD1	1,07E+01	3,56E+01
RvD5	6,21E-01	2,07E+00
MaR1	2,15E+00	7,16E+00
PD1	4,84E-01	1,61E+00
LTB4	5,88E-01	1,96E+00
12,13-DiHOME	3,73E-01	1,24E+00
9,10-DiHOME	1,94E+00	6,48E+00
14,15-DiHETrE	9,34E-02	3,11E-01
19,20 DiHDPA	1,45E+00	4,85E+00
11,12-DiHETrE	1,19E+00	3,98E+00
8,9-DiHETrE	1,62E+00	5,38E+00
9-HOTrE	4,36E+01	1,45E+02
13-HOTrE	2,51E+00	8,38E+00
18-HEPE	1,35E+00	4,51E+00
12-HEPE	1,22E+00	4,05E+00
13-HODE	5,11E+00	1,70E+01
12,13-EpOME	1,05E+01	3,50E+01
9-HODE	5,34E+01	1,78E+02
9,10-EpOME	6,60E+00	2,20E+01
13-oxoODE	2,00E+01	6,67E+01
9-oxoODE	1,16E+01	3,86E+01
17-HETE	1,08E+00	3,61E+00
15-HETE	1,09E+00	3,64E+00
11-HETE	1,82E+00	6,06E+00
8-HETE	1,90E+00	6,33E+00
12-HETE	1,30E+00	4,33E+00
9-HETE	1,62E+00	5,41E+00
5-HETE	3,56E+00	1,19E+01
15-oxoETE	3,96E+00	1,32E+01
12-oxoETE	3,60E-01	1,20E+00
5-oxoETE	6,61E-01	2,20E+00
20-HDoHE	2,21E-01	7,36E-01
16-HDoHE	2,56E-01	8,54E-01
13-HDoHE	7,92E-02	2,64E-01
14-HDoHE	5,65E-02	1,88E-01
10-HDoHE	1,29E-01	4,32E-01
11-HDoHE	7,31E-02	2,44E-01
7-HDoHE	1,80E+00	6,01E+00
4-HDoHE	1,68E+00	5,62E+00
AA	2,90E+02	9,68E+02
DHA	8,29E+01	2,76E+02
EPA	5,75E+01	1,92E+02
