# Supplementary material for: HPLC-MS/MS Oxylipin Analysis of Plasma from Amyotrophic Lateral Sclerosis Patients
Source: Biomedicines. 2022 Mar 15;10(3):674. doi: 10.3390/biomedicines10030674 (PMC8945419; doi:10.3390/biomedicines10030674)
Supplement: Supplementary file 1 [file biomedicines-10-00674-s001.zip › Table S6 - SPM LOD & LOQ.pdf]

Table S6. Calibration curves and LOD and LOQ values for all SPM and related metabolites analyzed by HPLC-MS/MS.

SPM	Internal Standard	slope	offset	r	LOD (pg)	LOQ (pg)
LXA4	(d5) MaR1	5.62113	-0.0023	0.99813	1.25	2.5
LXB4	(d5) MaR1	1.95314	0.00523	0.99763	10	20
RvE1	(d4) RvE1	1.7233	0.00245	0.99944	10	20
18-HEPE	(d8) 15-HETE	0.46928	-0.00117	0.99953	5	5
PD1	(d5) MaR1	2.81574	-0.00558	0.99989	2.5	5
MaR1	(d5) MaR1	1.40761	0.00367	0.99998	2.5	5
RvD1	(d5) RvD1	0.58949	0.01048	0.99885	1.25	2.5
RvD2	(d5) RvD1	0.30054	3.00E-04	0.99769	2.5	10
RvD3	(d5) RvD1	0.6528	0.00114	0.99851	2.5	5
RvD5	(d5) MaR1	4.17593	0.00295	0.99971	1.25	2.5
7-HDoHE	(d8) 5-HETE	0.67953	3.73E-04	0.9999	1.25	2.5
14-HDoHE	(d8) 12-HETE	4.62304	-0.00544	0.99977	0.313	0.625
16-HDoHE	(d8) 15-HETE	2.03503	-0.0075	0.99956	0.625	1.25
